# Novel total syntheses of oxoaporphine alkaloids enabled by mild Cu-catalyzed tandem oxidation/aromatization of 1-Bn-DHIQs†

**DOI:** 10.1039/c8ra05338c

**Published:** 2018-08-14

**Authors:** Bo Zheng, Hui-Ya Qu, Tian-Zhuo Meng, Xia Lu, Jie Zheng, Yun-Gang He, Qi-Qi Fan, Xiao-Xin Shi

**Affiliations:** Shanghai Key Laboratory of Chemical Biology and Department of Pharmaceutical Engineering, School of Pharmacy, East China University of Science and Technology 130 Mei-Long Road Shanghai 200237 P. R. China xxshi@ecust.edu.cn

## Abstract

Novel total syntheses of oxoaporphine alkaloids such as liriodenine, dicentrinone, cassameridine, lysicamine, oxoglaucine and *O*-methylmoschatoline were developed. The key step of these total syntheses is Cu-catalyzed conversion of 1-benzyl-3,4-dihydro-isoquinolines (1-Bn-DHIQs) to 1-benzoyl-isoquinolines (1-Bz-IQs) *via* tandem oxidation/aromatization. This novel Cu-catalyzed conversion has been studied in detail, and was successfully used for constructing the 1-Bz-IQ core.

## Abstract 2

Novel total syntheses of several oxoaporphine alkaloids were developed. The Cu-catalyzed conversion of 1-benzyl-3,4-dihydro-isoquinolines (1-Bn-DHIQs) to 1-benzoyl-isoquinolines (1-Bz-IQs) as the key step of these total syntheses has also been investigated in detail.

## Graphical abstract

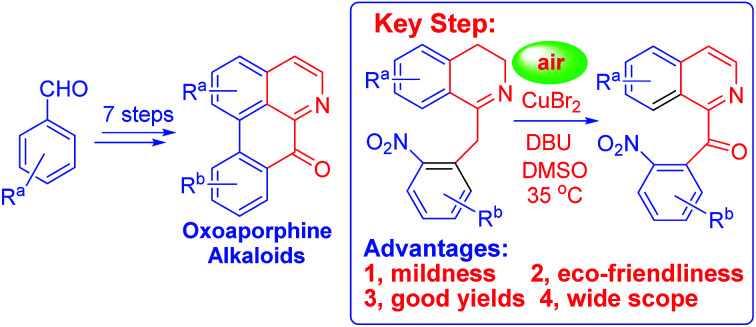

## Introduction

**Fig. 1:**
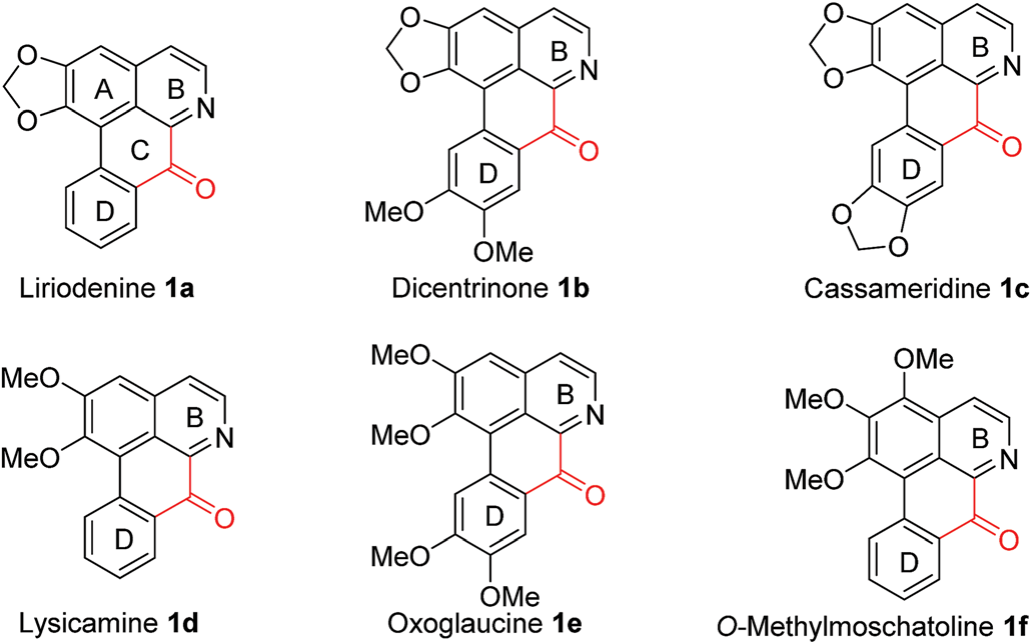
The targeted oxoaporphine alkaloids 1a–f.

Oxoaporphines, which have a characteristic carbonyl junction between aromatic B and D rings (see Fig. 1), are a sub-class of aporphinoid alkaloids.^1^ Oxoaporphine alkaloids are widespread in the kingdom of plants.^1,2^ For example, liriodenine 1a,^3^ dicentrinone 1b,^4^ cassameridine 1c,^5^ lysicamine 1d,^6^ oxoglaucine 1e^7^ and *O*-methylmoschatoline 1f^8^ (also referred to as liridine^9^ and homomoschatoline^10^) have been isolated from various botanic natural resources.^3–10^ Since oxoaporphine alkaloids 1a–f have shown a broad spectrum of interesting biological activities,^11^ so the total syntheses of them have aroused much interest from chemists.^12^ However, efficient, benign and practical total syntheses of these alkaloids remained highly desirable. Therefore, we herein report novel total syntheses of oxoaporphine alkaloids 1a–f*via* a key Cu-catalyzed conversion of 1-benzyl-3,4-dihydro-isoquinolines (1-Bn-DHIQs) to 1-benzoyl-isoquinolines (1-Bz-IQs).

**Scheme 1:**
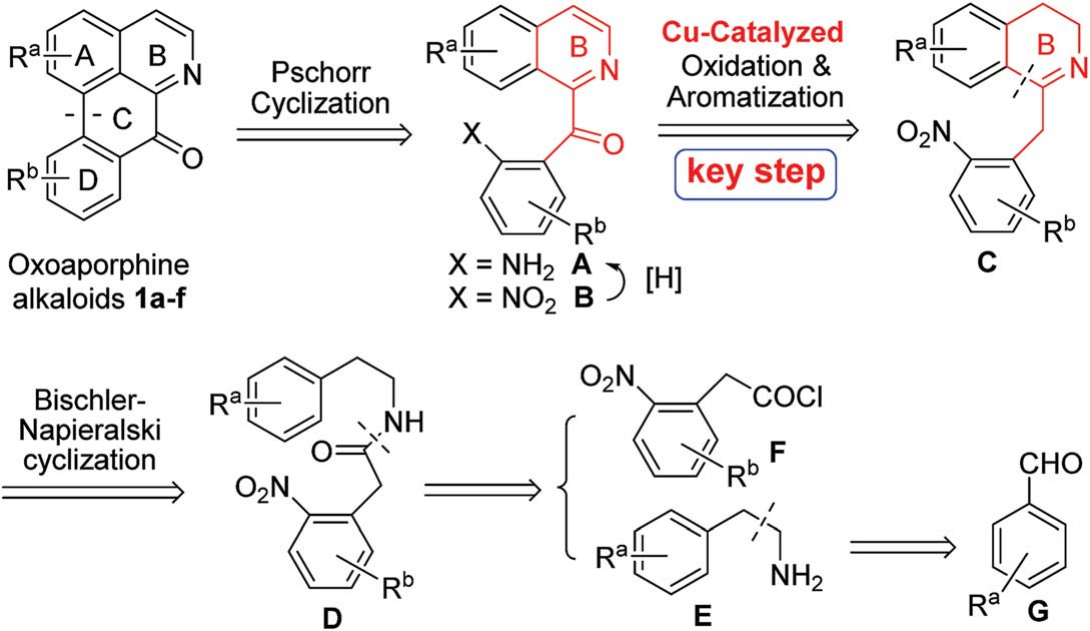
A novel general retrosynthetic analysis of oxoaporphine alkaloids 1a–f.

A general retrosynthetic analysis of the above several oxoaporphine alkaloids 1a–f is depicted in Scheme 1. As can be seen from the Scheme 1, C-ring could be constructed *via* Pschorr cyclization of anilines A, which could be obtained from the reduction of nitro groups of compounds B. The 1-Bz-IQ core of compounds B could be constructed *via* Cu-catalyzed tandem oxidation/aromatization of 1-Bn-DHIQs C. B-ring of compounds C could be constructed *via* Bischler–Napieralski cyclization of amides D, which could be derived from amines E and acyl chlorides F. Amines E could be prepared from aryl aldehydes G.

## Results and discussion

**Scheme 2:**
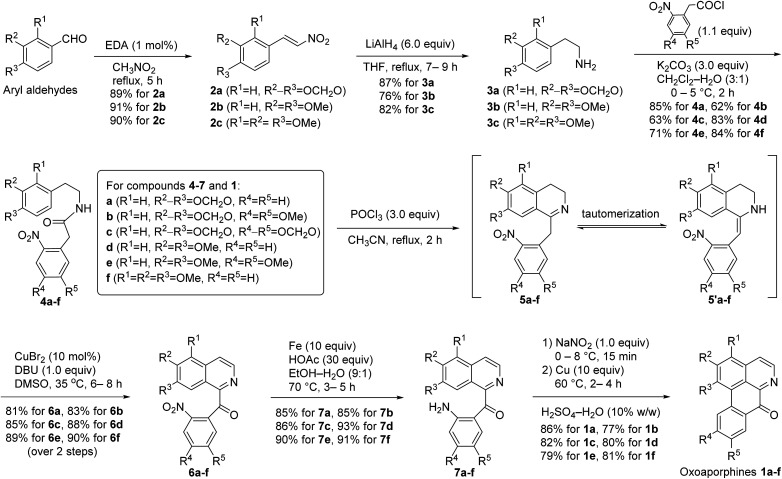
Total syntheses of the targeted oxoaporphine alkaloids 1a–f starting from aryl aldehydes.

According to the above general retrosynthetic analysis, novel total syntheses of the six targeted oxoaporphine alkaloids 1a–f are depicted in Scheme 2. As can be seen from the Scheme 2, EDA (ethylenediamine)-catalyzed condensation of aryl aldehydes with nitromethane^13^ first produced nitroalkenes 2a–c in high yields. Next, when compounds 2a–c were treated with 6.0 equiv. of LiAlH_4_ in tetrahydrofuran at reflux, simultaneous reduction of both nitro group and double bond occurred in one-pot to furnish 2-aryl ethanamines 3a–c, which then immediately exposed to 1.1 equiv. of 2-arylacetyl chlorides and 3.0 equiv. of K_2_CO_3_ at 0 °C in a mixed solvent of dichloromethane and water (3 : 1) to afford amides 4a–f. Subsequently, treatment of compounds 4a–f with 3.0 equiv. of POCl_3_ in anhydrous acetonitrile at reflux gave 1-Bn-DHIQs 5a–f*via* Bischler–Napieralski cyclization.^14^ It was observed that compounds 5a–f and compounds 5′a–f were interchangeable at room temperature, we failed to separate them by chromatography due to rapid tautomerization between imines (5a–f) and enamines (5′a–f), so they were used as such for the next step without separation; when the tautomeric mixture of 5a–f and 5′a–f were treated with 0.1 equiv. (10 mol%) of anhydrous CuBr_2_ and 1.0 equiv. of 1,8-diazabicyclo[5.4.0]undec-7-ene (DBU) in dimethyl sulfoxide (DMSO) at 35 °C under an open air (O_2_) atmosphere, Cu-catalyzed tandem benzylic oxidation and aromatization occurred smoothly in one-pot to afford 1-Bz-IQs 6a–f in good yields over two steps (from 4a–f). Nitro (NO_2_) groups of compounds 6a–f could be rapidly reduced by acid-activated iron;^15^ when compounds 6a–f were treated with 10 equiv. of iron powder and 30 equiv. of HOAc at 70 °C under N_2_ atmosphere in aqueous ethanol (EtOH/H_2_O = 9 : 1), reduction of nitro groups took place smoothly to produce anilines 7a–f in high yields. Finally, compounds 7a–f were first treated with 1.0 equiv. of NaNO_2_ at 0–8 °C to produce diazonium intermediates, which were then immediately treated with 10 equiv. of copper powder at 60 °C in an aqueous H_2_SO_4_ (10% w/w) solution, Pschorr cyclization^16^ happened rapidly to afford oxoaporphines 1a–f in good yields. Thus, the targeted oxoaporphine alkaloids liriodenine 1a, dicentrinone 1b, cassameridine 1c, lysicamine 1d, oxoglaucine 1e and *O*-methyl-moschatoline 1f were synthesized from aryl aldehydes *via* 7 steps in 39%, 26%, 29%, 38%, 31% and 41% overall yields, respectively.

In the above-described total syntheses of oxoaporphine alkaloids 1a–f, the key step is Cu-catalyzed conversion of 1-Bn-THIQs 5a–f to 1-Bz-IQs 6a–f. This key conversion can be achieved by some known methods.^17–21^ However, these known methods often suffered from drawbacks such as use of poisonous and hazardous strong oxidants including SeO_2_,^17^ Pb(OAc)_4_,^18^ and CAN,^19^ inconvenient use of the photoactivated singlet O_2_,^20^ or need of high reaction temperature^21^ (120 °C). Therefore, development of an efficient and benign method for this particular conversion might be very helpful for total syntheses of oxoaporphine alkaloids.

Copper is a cheap transition metal with low toxicity; and air (O_2_) is an eco-friendly clean oxidant. Hence, an increasing amount of copper-catalyzed aerobic oxidations of various compounds have been recently developed.^22^ For the above conversion of 1-Bn-THIQs 5a–f to 1-Bz-IQs 6a–f, CuBr_2_ was used as the catalyst, and air (O_2_) was used as the clean oxidant. So this Cu-catalyzed method might be much more benign, eco-friendly and practical than the previous known methods.^17–21^ Advantages such as mildness (at 35 °C), high efficiency and eco-friendliness prompted us to investigate the details of this potential green chemical method.

In order to know the scope and limitation of the method, more variously substituted 1-Bn-DHIQs 5 were prepared *via* Bischler–Napieralski cyclization, and a total of twenty substrates 5a–t have been tested for the CuBr_2_-catalyzed aerobic oxidation under the standard reaction conditions (see footnote of Table 1); the results are summarized in Table 1 (20 examples). As can be seen from the Table 1, the scope of the reaction is wide, it could be applicable to all the tested substrates, and afforded the desired 1-Bz-IQs 6a–t in good to high yields. We have found that 1-benzyl-3,4-dihydro-β-carbolines could undergo aerobic oxidation in DMSO to afford 1-benzoyl-β-carbolines without a copper catalyst,^23^ but herein we found that a copper catalyst is necessary for this one-pot aerobic oxidation, it was sluggish in the absence of copper catalyst. Several copper salts have been tested as the catalyst, CuBr_2_ obviously worked better than CuCl_2_, Cu(OAc)_2_, CuSO_4_, Cu_2_(OH)_2_CO_3_, CuO and CuI. Moreover, a base is also necessary for the reaction, DBU worked obviously better than Et_3_N, diisopropylethylamine, pyridine, *N*,*N*-dimethylaminopyridine (DMAP), 1,5-diazabicyclo[4,3,0]non-5-ene (DBN), Na_2_CO_3_ and K_2_CO_3_; DMSO as the solvent is better than DMF, CH_3_CN, THF, EtOH, Me_2_CO, CH_2_Cl_2_ and 1,4-dioxane.

**Table 1:** CuBr_2_-catalyzed conversion of variously substituted 1-Bn-DHIQs 5 to 1-Bz-IQs 6^a^

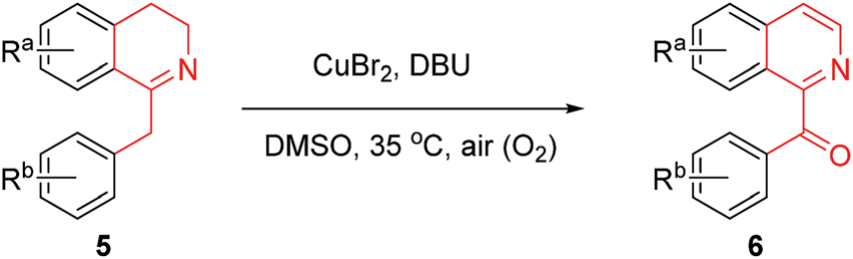
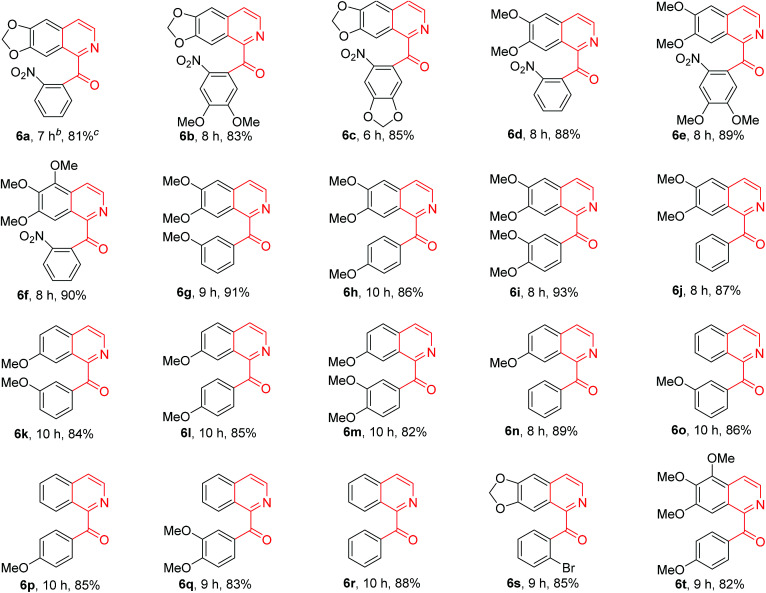

a Standard reaction conditions: 1-Bn-DHIQs 5 (2 mmol), CuBr_2_ (0.2 mmol), 1,8-diazabicyclo[5.4.0]undec-7-ene (DBU) (2 mmol), DMSO (6 mL), 35 °C (inner temperature), air (O_2_).

b Reaction time.

c Isolated yields.

**Scheme 3:**
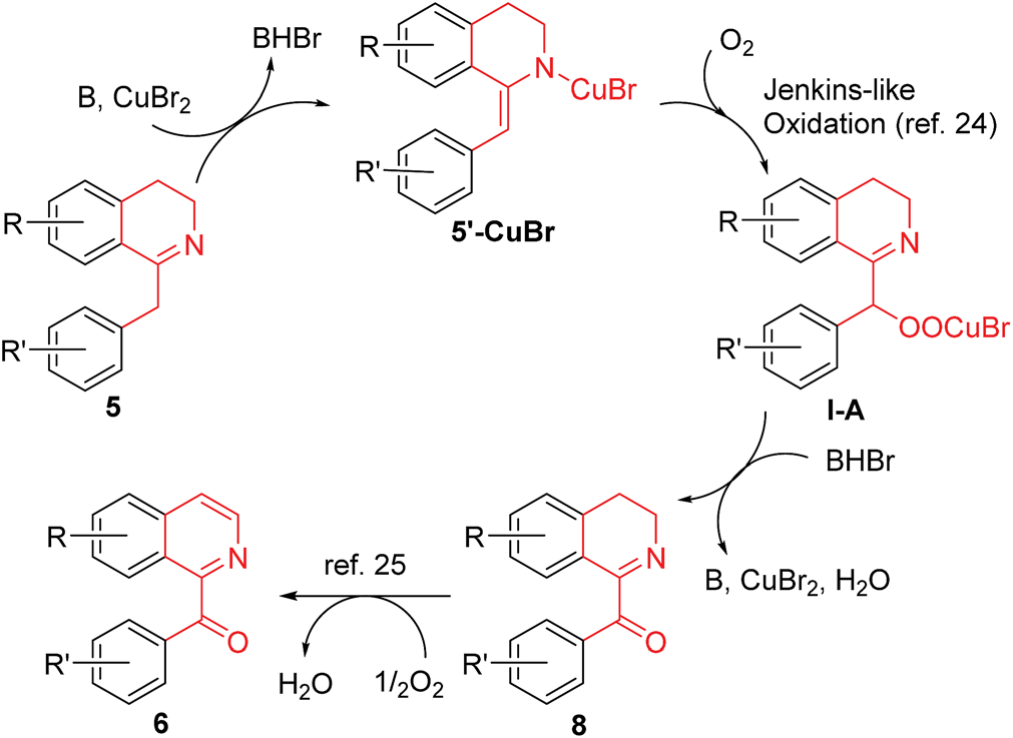
A possible mechanism for the CuBr_2_-catalyzed conversion of 1-Bn-DHIQs 5 to 1-Bz-IQs 6.

A possible mechanism for the CuBr_2_-catalyzed conversion of 1-Bn-DHIQs 5 to 1-Bz-IQs 6 was proposed in Scheme 3. 1-Bn-DHIQs 5 would first react with CuBr_2_ and a base (B = DBU, *etc.*) to form enamine-CuBr complexes 5′-CuBr, which would then undergo Jenkins-like aerobic oxidation^24^ to produce peroxide-CuBr complexes I-A. The intermediate Cu-complexes I-A would be unstable enough to decompose immediately to furnish 1-Bz-DHIQs 8. Compounds 8 would finally undergo DBU-promoted oxidation by O_2_ to produce 1-Bz-IQs 6 according to Kumar's reports.^25^ The intermediate compounds 8 could be detected by TLC during the reaction, and could also be isolated if the reaction was stopped at a middle point. For example, when CuBr_2_-catalyzed conversion of compound 5a to compound 6a was stopped at 3 h, the intermediate compound 8a was isolated in 38% yield.

## Conclusions

In conclusion, novel total syntheses of oxoaporphine alkaloids including liriodenine 1a, dicentrinone 1b, cassameridine 1c, lysicamine 1d, oxoglaucine 1e and *O*-methyl-moschatoline 1e were achieved *via* a benign and ecofriendly Cu-catalyzed general approach. They were synthesized from the cheap and commercially available aryl aldehydes *via* seven steps in 39%, 26%, 29%, 38%, 31% and 41% overall yields, respectively.

In addition, a novel practical Cu-catalyzed conversion of 1-Bn-DHIQs 5 to 1-Bz-IQs 6, which was used as the key step in the above total syntheses, has also been investigated in detail; this Cu-catalyzed method has some advantages such as mildness, eco-friendliness, wide scope, ease of experimental procedure and high yields. It would provide a new general basic approach towards the total syntheses of oxoaporphine alkaloids and their derivatives.

## Experimental

### General

^1^H NMR and ^13^C NMR spectra were acquired on a Bruker AM 400 instrument, chemical shifts are given on the *δ* scale as parts per million (ppm) with tetramethylsilane (TMS) as the internal standard. IR spectra were recorded on a Nicolet Magna IR-550 instrument. Mass spectra were performed with a HP1100 LC-MS spectrometer. Melting points were measured on a Mei-TEMP II melting point apparatus. Column chromatography was performed on silica gel (Qingdao Chemical Factory). All reagents and solvents were analytically pure, and were used as such as received from the chemical suppliers.

### Preparation of nitroalkenes 2a–c

An aryl aldehyde (∼40 mmol) was dissolved in nitromethane (30 mL), and then ethylenediamine (0.025 g, 0.416 mmol) was added. The resulting solution was then heated and stirred at reflux for 5 h. After the reaction was complete (checked by TLC, eluent: CH_2_Cl_2_/hexane = 3 : 2), the solution was concentrated under vacuum to give crude product as yellow solid, which was triturated in aqueous methanol (MeOH/H_2_O = 9 : 1), and was then filtered by suction. Compounds 2a–c were thus obtained in 89%, 90% and 91% yields, respectively. Characterization data of compounds 2a–c are as follows:

#### (*E*)-5-(2-Nitrovinyl)benzo[*d*][1,3]dioxole (2a)

Yellow crystals, mp 158–159 °C. ^1^H NMR (400 MHz, CDCl_3_) *δ* 7.93 (d, *J* = 13.5 Hz, 1H), 7.48 (d, *J* = 13.5 Hz, 1H), 7.09 (d, *J* = 7.8 Hz, 1H), 7.00 (s, 1H), 6.88 (d, *J* = 7.8 Hz, 1H), 6.07 (s, 2H); ^13^C NMR (100 MHz, CDCl_3_) *δ* 151.39, 148.77, 139.13, 135.37, 126.69, 124.18, 109.08, 106.98, 102.10.

#### (*E*)-1,2-Dimethoxy-4-(2-nitrovinyl)benzene (2b)

Pale yellow crystals, mp 141–142 °C. ^1^H NMR (400 MHz, CDCl_3_) *δ* 7.97 (d, *J* = 13.6 Hz, 1H), 7.56 (d, *J* = 13.6 Hz, 1H), 7.19 (d, *J* = 8.2 Hz, 1H), 7.02 (s, 1H), 6.92 (d, *J* = 8.2 Hz, 1H), 3.95 (s, 3H), 3.93 (s, 3H); ^13^C NMR (100 MHz, CDCl_3_) *δ* 152.78, 149.51, 139.39, 135.13, 124.71, 122.78, 111.30, 110.18, 56.10, 56.02.

#### (*E*)-1,2,3-Trimethoxy-4-(2-nitrovinyl)benzene (2c)

Pale yellow crystals, mp 192–193 °C. ^1^H NMR (400 MHz, CDCl_3_) *δ* 8.09 (d, *J* = 13.6 Hz, 1H), 7.77 (d, *J* = 13.6 Hz, 1H), 7.21 (d, *J* = 8.8 Hz, 1H), 6.73 (d, *J* = 8.8 Hz, 1H), 4.00 (s, 3H), 3.93 (s, 3H), 3.88 (s, 3H); ^13^C NMR (100 MHz, CDCl_3_) *δ* 157.36, 154.27, 142.39, 136.52, 135.37, 126.65, 117.02, 107.68, 61.19, 60.94, 56.20.

### Preparation of 2-aryl ethanamines 3a–c

A solution of compound 2 (∼18 mmol) in THF (40 mL) was dropwise added into a stirred suspension of LiAlH_4_ (4.102 g, 108.1 mmol) in THF (100 mL) at 0 °C over 20 min. After the addition was finished, the mixture was then heated and stirred at reflux for 7–9 h. The mixture was cooled to 0 °C by an ice-bath. While the mixture was vigorously stirred, water (5 mL) was dropwise added into the reaction mixture over 30 min, and an aqueous solution of NaOH (28 mL, 15% w/w) was then slowly added into the mixture at 0 °C. The ice-bath was removed, and the mixture was further stirred at room temperature for 3 h. The mixture was filtered through a thin layer of celite, and the filter cake was washed twice with THF (2 × 30 mL). The filtrates were combined and dried over anhydrous MgSO_4_. The solution was concentrated under vacuum to give crude product as yellow oil, which was purified by formation of hydrochloride ammonium salt. Pure 2-aryl ethanamines 3a–c were obtained after neutralization in 87%, 76% and 82% yields, respectively. Characterization data of compounds 3a–c are as follows:

#### 2-(Benzo[*d*][1,3]dioxol-5-yl)ethanamine (3a)

^1^H NMR (400 MHz, CDCl_3_) *δ* 6.74 (d, *J* = 7.8 Hz, 1H), 6.69 (s, 1H), 6.64 (d, *J* = 7.8 Hz, 1H), 5.92 (s, 2H), 2.91 (t, *J* = 6.9 Hz, 2H), 2.67 (t, *J* = 6.9 Hz, 2H), 1.56 (br. s, 3H, N*H*_2_); ^13^C NMR (100 MHz, CDCl_3_) *δ* 147.57, 145.80, 133.46, 121.55, 109.02, 108.09, 100.71, 43.49, 39.49.

#### 2-(3,4-Dimethoxyphenyl)ethanamine (3b)

^1^H NMR (400 MHz, CDCl_3_) *δ* 6.79 (d, *J* = 7.9 Hz, 1H), 6.76 (d, *J* = 7.9 Hz, 1H), 6.73 (s, 1H), 3.87 (s, 3H), 3.85 (s, 3H), 2.93 (t, *J* = 6.9 Hz, 2H), 2.68 (t, *J* = 6.9 Hz, 2H), 1.43 (br. s, 2H, N*H*_2_); ^13^C NMR (100 MHz, CDCl_3_) *δ* 148.81, 147.34, 132.35, 120.65, 111.96, 111.22, 55.83, 55.73, 43.58, 39.51.

#### 2-(2,3,4-Trimethoxyphenyl)ethanamine (3c)

^1^H NMR (400 MHz, CDCl_3_) *δ* 6.84 (d, *J* = 8.5 Hz, 1H), 6.62 (d, *J* = 8.5 Hz, 1H), 3.88 (s, 3H), 3.87 (s, 3H), 3.84 (s, 3H), 2.91 (t, *J* = 7.0 Hz, 2H), 2.71 (t, *J* = 7.0 Hz, 2H), 2.32 (br. s, 2H, N*H*_2_); ^13^C NMR (100 MHz, CDCl_3_) *δ* 152.31, 152.08, 142.29, 125.46, 124.35, 107.16, 60.92, 60.69, 55.97, 42.81, 33.84.

### Preparation of amides 4a–f

2-Aryl acetic acid (∼11 mmol) was dissolved in CH_2_Cl_2_ (20 mL), and SOCl_2_ (1.790 g, 15.05 mmol) was added. The resulting solution was then heated and stirred at reflux for 4 h. The reaction solution was concentrated under vacuum to dryness, oily residue was then dissolved in dry CH_2_Cl_2_ (10 mL), the solution was immediately used bellow. 2-Aryl ethanamine 3 (10 mmol) was dissolved in 40 mL CH_2_Cl_2_, and an aqueous solution of K_2_CO_3_ (4.150 g, 30.03 mmol) in water (30 mL) was added. The biphasic mixture was cooled by an ice-bath, and was stirred at 0 to 5 °C. The above freshly prepared solution of 2-aryl acetyl chloride was added dropwise into the reaction mixture over 2 min. After the addition was finished, stirring was continued at 0 to 5 °C for 2 h. When the reaction was complete (checked by TLC, eluent: EtOAc/hexane = 1 : 3), the reaction mixture was transferred into a separatory funnel. Two phases were separated, and the aqueous phase was extracted again with CH_2_Cl_2_ (20 mL). The organic extracts were combined, dried over anhydrous MgSO_4_, and then concentrated under vacuum to give crude product as pale yellow solid, which was purified by flash chromatography (eluent: EtOAc/hexane = 1 : 4). Amides 4a–f were thus obtained in 85%, 62%, 63%, 83%, 71% and 84% yields, respectively. Characterization data of compounds 4a–f are as follows:

#### *N*-(2-(Benzo[*d*][1,3]dioxol-5-yl)ethyl)-2-(2-nitrophenyl)aceta-mide (4a)

White crystals, mp 125–126 °C. ^1^H NMR (400 MHz, CDCl_3_) *δ* 8.03 (d, *J* = 8.2 Hz, 1H), 7.60 (dd, *J*_1_ = 8.0 Hz, *J*_2_ = 7.8 Hz, 1H), 7.48–7.42 (m, 2H), 6.69 (d, *J* = 7.9 Hz, 1H), 6.60 (s, 1H), 6.55 (d, *J* = 7.9 Hz, 1H), 5.92 (s, 2H), 5.84 (br. s, 1H, CON*H*), 3.79 (s, 2H), 3.49–3.42 (m, 2H), 2.69 (t, *J* = 6.8 Hz, 2H); ^13^C NMR (100 MHz, CDCl_3_) *δ* 168.93, 148.80, 147.72, 146.11, 133.57, 133.33, 132.44, 130.41, 128.37, 125.12, 121.61, 109.03, 108.30, 100.87, 41.06, 40.89, 35.24; IR (KBr film) 3280, 3070, 2921, 1640, 1552, 1521, 1492, 1350, 1247, 1192, 1034, 923, 809, 790, 715 cm^−1^.

#### *N*-(2-(Benzo[*d*][1,3]dioxol-5-yl)ethyl)-2-(4,5-dimethoxy-2-nitrophenyl)acetamide (4b)

White crystals, mp 206–207 °C. ^1^H NMR (400 MHz, DMSO-*d*_6_) *δ* 7.97 (t, *J* = 5.8 Hz, 1H, CON*H*), 7.64 (s, 1H), 7.05 (s, 1H), 6.81 (d, *J* = 7.9 Hz, 1H), 6.79 (s, 1H), 6.65 (d, *J* = 7.9 Hz, 1H), 5.96 (s, 2H), 3.87 (s, 3H), 3.85 (s, 3H), 3.82 (s, 2H), 3.25–3.17 (m, 2H), 2.61 (t, *J* = 7.2 Hz, 2H); ^13^C NMR (100 MHz, DMSO-*d*_6_) *δ* 168.62, 152.57, 147.16, 147.10, 145.42, 140.92, 133.25, 126.01, 121.43, 115.36, 108.98, 108.02, 107.94, 100.58, 56.10, 55.90, 40.78, 40.00, 34.84; IR (KBr film) 3270, 3092, 2933, 1641, 1580, 1525, 1502, 1420, 1330, 1274, 1239, 1186, 1063, 1034, 923, 875, 804, 748 cm^−1^.

#### *N*-(2-(Benzo[*d*][1,3]dioxol-5-yl)ethyl)-2-(6-nitrobenzo[*d*][1,3]dioxol-5-yl)acetamide (4c)

White crystals, mp 208–209 °C. ^1^H NMR (400 MHz, DMSO-*d*_6_) *δ* 8.02 (t, *J* = 5.7 Hz, 1H, CON*H*), 7.63 (s, 1H), 7.01 (s, 1H), 6.82 (d, *J* = 7.9 Hz, 1H), 6.79 (s, 1H), 6.65 (d, *J* = 7.9 Hz, 1H), 6.22 (s, 2H), 5.96 (s, 2H), 3.76 (s, 2H), 3.26–3.18 (m, 2H), 2.61 (t, *J* = 7.2 Hz, 2H);^13^C NMR (100 MHz, DMSO-*d*_6_) *δ* 168.40, 151.26, 147.11, 146.53, 145.43, 142.63, 133.20, 128.10, 121.45, 111.87, 108.99, 108.03, 105.08, 103.20, 100.58, 40.61, 39.95, 34.82; IR (KBr film) 3280, 3064, 2920, 1637, 1548, 1524, 1504, 1480, 1442, 1378, 1328, 1249, 1192, 1032, 924, 877, 816, 722 cm^−1^.

#### *N*-(3,4-Dimethoxyphenethyl)-2-(2-nitrophenyl)acetamide (4d)

White crystals, mp 129–130 °C. ^1^H NMR (400 MHz, CDCl_3_) *δ* 8.01 (d, *J* = 8.2 Hz, 1H), 7.58 (dd, *J*_1_ = 8.0 Hz, *J*_2_ = 8.2 Hz, 1H), 7.46–7.40 (m, 2H), 6.75 (d, *J* = 8.1 Hz, 1H), 6.69 (s, 1H), 6.67 (d, *J* = 8.1 Hz, 1H), 3.85 (s, 3H), 3.84 (s, 3H), 3.78 (s, 2H), 3.52–3.45 (m, 2H), 2.73 (t, *J* = 7.0 Hz, 2H); ^13^C NMR (100 MHz, CDCl_3_) *δ* 168.88, 148.98, 148.76, 147.62, 133.58, 133.37, 131.20, 130.38, 128.39, 125.12, 120.60, 111.90, 111.32, 55.92, 55.84, 40.94, 40.85, 35.10; IR (KBr film) 3424, 3065, 2944, 2839, 1651, 1602, 1479, 1280, 1256, 1163, 1125, 1028, 935, 823, 718 cm^−1^; HRMS (ESI) *m*/*z* calcd for C_18_H_21_N_2_O_5_ [M + H]^+^: 345.1450, found: 345.1451.

#### *N*-(3,4-Dimethoxyphenethyl)-2-(4,5-dimethoxy-2-nitrophenyl)acetamide (4e)

White crystals, mp 131–132 °C. ^1^H NMR (400 MHz, CDCl_3_) *δ* 7.60 (s, 1H), 6.83 (s, 1H), 6.74 (d, *J* = 8.1 Hz, 1H), 6.67 (s, 1H), 6.65 (d, *J* = 8.1 Hz, 1H), 5.96 (br. s, 1H, CON*H*), 3.96 (s, 3H), 3.94 (s, 3H), 3.85 (s, 3H), 3.84 (s, 3H), 3.77 (s, 2H), 3.53–3.44 (m, 2H), 2.73 (t, *J* = 6.8 Hz, 2H); ^13^C NMR (100 MHz, CDCl_3_) *δ* 168.90, 153.35, 148.96, 148.07, 147.59, 140.80, 131.14, 125.48, 120.56, 114.44, 111.88, 111.23, 108.15, 56.57, 56.35, 55.87, 55.82, 41.39, 40.90, 35.09; IR (KBr film) 3467, 3072, 2945, 2832, 1653, 1600, 1475, 1282, 1251, 1168, 1125, 1018, 925, 827, 724 cm^−1^; HRMS (ESI) *m*/*z* calcd for C_20_H_25_N_2_O_7_ [M + H]^+^: 405.1662, found: 405.1665.

#### *N*-(2,3,4-Trimethoxyphenethyl)-2-(2-nitrophenyl)acetamide (4f)

White crystals, mp 106–107 °C. ^1^H NMR (400 MHz, CDCl_3_) *δ* 8.02 (d, *J* = 7.8 Hz, 1H), 7.57 (d, *J* = 7.2 Hz, 1H), 7.48–7.41 (m, 2H), 6.78 (d, *J* = 8.2 Hz, 1H), 6.58 (d, *J* = 8.2 Hz, 1H), 6.21 (br. s, 1H, CON*H*), 3.85 (s, 3H), 3.845 (s, 3H), 3.843 (s, 3H), 3.80 (s, 2H), 3.47–3.41 (m, 2H), 2.73 (t, *J* = 6.8 Hz, 2H); ^13^C NMR (100 MHz, CDCl_3_) *δ* 168.94, 152.58, 151.80, 148.89, 142.19, 133.49, 133.42, 130.53, 128.28, 125.06, 124.74, 124.51, 107.43, 60.98, 60.74, 56.00, 40.95, 40.94, 29.63; IR (KBr, film) 3420, 3293, 2958, 2939, 1644, 1548, 1524, 1495, 1467, 1417, 1349, 1240, 1101, 933, 792, 716 cm^−1^; HRMS (ESI) *m*/*z* calcd for C_19_H_23_N_2_O_6_ [M + H]^+^: 375.1556, found: 375.1537.

### Preparation of 1-benzyl-3,4-dihydroisoquinolines 5

A corresponding amide 4 (∼5 mmol) was dissolved in anhydrous acetonitrile (40 mL), and phosphorus oxychloride (2.350 g, 15.33 mmol) was slowly added into the mixture. The resulting solution was then heated and stirred at reflux for 2 h. After the reaction was complete (checked by TLC, eluent: CH_2_Cl_2_/hexane = 2 : 1), the solution was concentrated under vacuum to dryness, the residue was dissolved in CH_2_Cl_2_ (50 mL). An aqueous solution of K_2_CO_3_ (20 mL, 15% w/w) was added. After the mixture was vigorously stirred for 5 min, two phases were separated, and the aqueous phase was extracted again with CH_2_Cl_2_ (20 mL). The organic extracts were combined, dried over anhydrous MgSO_4_, and then concentrated under vacuum to give crude solid product as a tautomeric mixture of 1-benzyl-3,4-dihydroisoquinoline 5 and enamine 5′, which was used as such for the next step.

### General procedure for the CuBr_2_-catalyzed conversion of 1-benzyl-3,4-dihydroisoquinolines 5 to 1-benzoyl-isoquinolines 6

The above tautomeric mixture of 1-benzyl-3,4-dihydroisoquinoline 5 and enamine 5′ were dissolved in DMSO (10 mL), DBU (0.755 g, 4.959 mmol) and CuBr_2_ (0.113 g, 0.506 mmol) were added. The resulting solution was then stirred at 35 °C for 6–10 h (see Table 1) under an atmosphere of air. After the reaction was complete (checked by TLC, eluent: EtOAc/hexane = 1 : 4), a dilute ammonia aqueous solution (5% w/w, 50 mL) and CH_2_Cl_2_ (40 mL) were added into the mixture. Two phases were separated, and the aqueous phase was twice extracted again with CH_2_Cl_2_ (2 × 30 mL). The organic extracts were combined, and dried over anhydrous MgSO_4_. Removal of solvent by vacuum distillation gave crude product as pale yellow solid, which was purified by flash chromatography (eluent: EtOAc/CH_2_Cl_2_ = 1 : 10) to afford pure 1-benzoyl-isoquinoline 6 as crystals in a yield (over two steps) as indicated in Table 1. Characterization data of 1-benzoyl-isoquinolines 6a–t are as follows:

#### ([1,3]Dioxolo[4,5-*g*]isoquinolin-5-yl)(2-nitrophenyl)metha-none (6a)

Pale yellow crystals, mp 244–245 °C. ^1^H NMR (400 MHz, CDCl_3_-TFA) *δ* 8.42 (s, 1H), 8.30 (d, *J* = 8.2 Hz, 1H), 8.19 (d, *J* = 6.4 Hz, 1H), 8.09 (d, *J* = 6.4 Hz, 1H), 7.98 (dd, *J*_1_ = 7.5 Hz, *J*_1_ = 7.8 Hz, 1H), 7.92–7.84 (m, 2H), 7.40 (s, 1H), 6.40 (s, 2H); ^13^C NMR (100 MHz, CDCl_3_-TFA) *δ* 186.14, 156.93, 154.17, 146.24, 142.08, 141.68, 135.87, 134.04, 131.80, 131.08, 130.20, 126.47, 125.39, 124.52, 104.36, 103.78, 103.16; IR (KBr, film) 3077, 2917, 1684, 1576, 1518, 1460, 1348, 1275, 1214, 1036, 1017, 946, 867, 788, 706 cm^−1^; HRMS (ESI) *m*/*z* calcd for C_17_H_11_N_2_O_5_ [M + H]^+^: 323.0668, found: 323.0665.

#### (4,5-Dimethoxy-2-nitro-phenyl)([1,3]dioxolo[4,5-*g*]isoquino-lin-5-yl)methanone (6b)

Pale yellow crystals, mp 258–259 °C. ^1^H NMR (400 MHz, CDCl_3_-TFA) *δ* 8.37 (s, 1H), 8.16 (d, *J* = 6.3 Hz, 1H), 8.06 (d, *J* = 6.3 Hz, 1H), 7.70 (s, 1H), 7.38 (s, 1H), 7.27 (s, 1H), 6.40 (s, 2H), 4.07 (s, 3H), 4.05 (s, 3H); ^13^C NMR (100 MHz, CDCl_3_-TFA) *δ* 187.14, 155.78, 154.82, 153.24, 152.19, 144.56, 140.54, 139.96, 131.12, 126.75, 125.71, 124.55, 112.93, 107.34, 103.84, 103.81, 103.35, 56.88, 56.73; IR (KBr, film) 3096, 2916, 1682, 1579, 1520, 1500, 1463, 1397, 1326, 1282, 1261, 1228, 1210, 1077, 1034, 942, 863, 778, 758 cm^−1^; HRMS (ESI) *m*/*z* calcd for C_19_H_15_N_2_O_7_ [M + H]^+^: 383.0879, found: 383.0881.

#### ([1,3]Dioxolo[4,5-*g*]isoquinolin-5-yl)(6-nitrobenzo[*d*][1,3] dioxol-5-yl)methanone (6c)

Pale yellow crystals, mp 229–230 °C. ^1^H NMR (400 MHz, CDCl_3_-TFA) *δ* 8.38 (s, 1H), 8.21 (d, *J* = 6.3 Hz, 1H), 8.07 (d, *J* = 6.3 Hz, 1H), 7.64 (s, 1H), 7.38 (s, 1H), 7.16 (s, 1H), 6.39 (s, 2H), 6.30 (s, 2H); ^13^C NMR (100 MHz, CDCl_3_-TFA) *δ* 185.29, 156.70, 154.12, 153.93, 151.96, 142.54, 141.76, 141.62, 130.04, 128.24, 126.18, 124.54, 109.97, 105.43, 104.87, 104.22, 103.62, 103.61; IR (KBr, film) 3065, 2917, 1677, 1609, 1577, 1519, 1500, 1464, 1424, 1398, 1326, 1273, 1237, 1203, 1031, 988, 920, 871, 764 cm^−1^; HRMS (ESI) *m*/*z* calcd for C_18_H_11_N_2_O_7_ [M + H]^+^: 367.0566, found: 367.0565.

#### (6,7-Dimethoxyisoquinolin-1-yl)(2-nitrophenyl)methanone (6d)

Pale yellow crystals, mp 195–196 °C. ^1^H NMR (400 MHz, CDCl_3_) *δ* 8.75 (s, 1H), 8.24 (d, *J* = 5.6 Hz, 1H), 8.15 (d, *J* = 5.6 Hz, 1H), 7.80 (dd, *J*_1_ = 8.0 Hz, *J*_2_ = 7.7 Hz, 1H), 7.73–7.58 (m, 3H), 7.10 (s, 1H), 4.14 (s, 3H), 4.05 (s, 3H); ^13^C NMR (100 MHz, CDCl_3_) *δ* 195.63, 153.08, 152.47, 148.35, 147.59, 140.15, 137.94, 134.54, 134.02, 130.29, 129.66, 123.67, 123.66, 123.55, 104.74, 104.53, 56.26, 56.07; IR (KBr film) 2945, 2835, 1650, 1600, 1477, 1329, 1282, 1255, 1168, 1125, 1020, 915, 809 cm^−1^; HRMS (ESI) *m*/*z* calcd for C_18_H_15_N_2_O_5_ [M + H]^+^: 339.0981, found: 339.0985.

#### (6,7-Dimethoxyisoquinolin-1-yl)(4,5-dimethoxy-2-nitrophenyl)methanone (6e)

Pale yellow crystals, mp 212–213 °C. ^1^H NMR (400 MHz, CDCl_3_) *δ* 8.65 (s, 1H), 8.24 (d, *J* = 5.4 Hz, 1H), 7.65 (s, 1H), 7.61 (d, *J* = 5.4 Hz, 1H), 7.12 (s, 1H), 7.09 (s, 1H), 4.13 (s, 3H), 4.04 (s, 3H), 4.02 (s, 3H), 4.01 (S, 3H); ^13^C NMR (100 MHz, CDCl_3_) *δ* 195.33, 153.69, 153.06, 152.30, 149.68, 149.06, 140.57, 140.01, 134.44, 132.05, 123.54, 111.05, 106.46, 104.72, 104.71, 104.61, 56.64, 56.57, 56.22, 56.07; IR (KBr film) 2981, 2845, 1652, 1603, 1473, 1328, 1282, 1255, 1166, 1123, 1021, 934, 865, 812 cm^−1^; HRMS (ESI) *m*/*z* calcd for C_20_H_19_N_2_O_7_ [M + H]^+^: 399.1192, found: 399.1190.

#### (2-Nitrophenyl)(5,6,7-trimethoxyisoquinolin-1-yl)methanone (6f)

Pale yellow crystals, mp 113–114 °C. ^1^H NMR (400 MHz, CDCl_3_) *δ* 8.56 (s, 1H), 8.26 (d, *J* = 5.2 Hz, 1H), 8.15 (d, *J* = 8.0 Hz, 1H), 8.00 (d, *J* = 5.2 Hz, 1H), 7.80 (dd, *J*_1_ = 8.0 Hz, *J*_2_ = 7.7 Hz, 1H), 7.70 (d, *J* = 7.5 Hz, 1H), 7.65 (dd, *J*_1_ = 7.7 Hz, *J*_2_ = 7.5 Hz, 1H), 4.12 (s, 3H), 4.05 (s, 3H), 4.04 (s, 3H); ^13^C NMR (100 MHz, CDCl_3_) *δ* 195.45, 156.02, 148.63, 147.48, 146.51, 144.13, 139.55, 137.92, 134.07, 130.33, 129.84, 129.69, 124.45, 123.58, 119.13, 100.86, 61.70, 61.23, 56.26; IR (KBr, film) 2938, 1687, 1612, 1577, 1528, 1476, 1353, 1274, 1243, 1124, 1048, 941, 855, 758, 637 cm^−1^; HRMS (ESI) *m*/*z* calcd for C_19_H_17_N_2_O_6_ [M + H]^+^: 369.1087, found: 369.1085.

#### (6,7-Dimethoxyisoquinolin-1-yl)(3-methoxyphenyl)methanone (6g)

White crystals, mp 168–169 °C. ^1^H NMR (400 MHz, CDCl_3_) *δ* 8.47 (d, *J* = 5.5 Hz, 1H), 7.66 (d, *J* = 5.5 Hz, 1H), 7.60 (s, 1H), 7.56 (s, 1H), 7.44 (d, *J* = 7.6 Hz, 1H), 7.37 (dd, *J* = 7.9 Hz, *J* = 7.6 Hz, 1H), 7.17 (d, *J* = 7.9 Hz, 1H), 7.14 (s, 1H), 4.05 (s, 3H), 3.96 (s, 3H), 3.85 (s, 3H); ^13^C NMR (100 MHz, CDCl_3_) *δ* 195.18, 159.62, 153.22, 153.02, 151.21, 140.05, 138.40, 134.05, 129.37, 123.99, 122.95, 121.64, 120.13, 114.55, 104.88, 103.95, 56.14, 56.08, 55.47. IR (KBr film) 2937, 2834, 1660, 1591, 1485, 1439, 1310, 1270, 1259, 1148, 1036, 746, 634 cm^−1^; HRMS (ESI) *m*/*z* calcd for C_19_H_18_NO_4_ [M + H]^+^: 324.1236, found: 324.1219.

#### (6,7-Dimethoxyisoquinolin-1-yl)(4-methoxyphenyl)methanone (6h)

White crystals, mp 157–158 °C (lit. 21 150–152 °C). ^1^H NMR (400 MHz, CDCl_3_) *δ* 8.46 (d, *J* = 5.5 Hz, 1H),7.96 (d, *J* = 8.9 Hz, 2H), 7.65 (d, *J* = 5.5 Hz), 7.57 (s, 1H), 7.15 (s, 1H), 6.96 (d, *J* = 8.9 Hz, 2H), 4.06 (s, 3H), 3.96 (s, 3H), 3.88 (s, 3H); ^13^C NMR (101 MHz, CDCl_3_) *δ* 193.92, 163.95, 153.69, 153.15, 151.00, 140.02, 133.98, 133.28 (2C), 129.81, 122.80, 121.29, 113.71 (2C), 104.86, 104.06, 56.12, 56.07, 55.55; IR (KBr film) 2961, 2940, 2836, 1650, 1607, 1511, 1442, 1321, 1264, 1156, 1054, 916, 862, 836, 613 cm^−1^.

#### (6,7-Dimethoxyisoquinolin-1-yl)(3,4-dimethoxyphenyl)metha-none (6i)

White crystals, mp 209–210 °C. ^1^H NMR (400 MHz, CDCl_3_) *δ* 8.47 (d, *J* = 5.5 Hz, 1H), 7.72 (s, 1H), 7.69 (d, *J* = 5.5 Hz, 1H), 7.55 (s, 1H), 7.42 (d, *J* = 8.4 Hz, 1H), 7.15 (s, 1H), 6.87 (d, *J* = 8.4 Hz, 1H), 4.06 (s, 3H), 3.97 (s, 3H), 3,96 (s, 3H), 3.95 (s, 3H); ^13^C NMR (100 MHz, CDCl_3_) *δ* 193.94, 153.79, 153.68, 153.15, 150.98, 149.00, 140.01, 133.93, 129.89, 126.91, 122.80, 121.26, 111.88, 109.94, 104.85, 104.01, 56.11, 56.09, 56.04, 55.99; IR (KBr film) 2970, 2934, 1657, 1505, 1460, 1270, 1227, 1140, 1025, 860, 749 cm^−1^; HRMS (ESI) *m*/*z* calcd for C_20_H_20_NO_5_ [M + H]^+^: 354.1341, found: 354.1343.

#### (6,7-Dimethoxyisoquinolin-1-yl)(phenyl)methanone (6j)

White crystals, mp 130–131 °C (lit. 21 131–132 °C). ^1^H NMR (400 MHz, CDCl_3_) *δ* 8.47 (d, *J* = 5.5 Hz, 1H), 7.96 (d, *J* = 8.0 Hz, 2H), 7.67 (d, *J* = 5.5 Hz, 1H), 7.65 (s, 1H), 7.63 (t, *J* = 7.8 Hz, 1H), 7.50 (dd, *J*_1_ = 7.8 Hz, *J*_2_ = 8.0 Hz, 2H), 7.15 (s, 1H), 4.06 (s, 3H), 3.97 (s, 3H); ^13^C NMR (100 MHz, CDCl_3_) *δ* 195.39, 153.20, 152.96, 151.22, 140.07, 137.11, 134.07, 133.42, 130.87 (2C), 128.38 (2C), 123.03, 121.66, 104.88, 103.99, 56.13, 56.08; IR (KBr film) 2971, 2841, 1657, 1510, 1456, 1260, 1232, 1154, 1052, 864, 709, 649 cm^−1^.

#### (7-Methoxyisoquinolin-1-yl)(3-methoxyphenyl)methanone (6k)

White crystals, mp 106–107 °C. ^1^H NMR (400 MHz, CDCl_3_) *δ* 8.50 (d, *J* = 5.5 Hz, 1H), 7.82 (d, *J* = 8.5 Hz, 1H), 7.75 (d, *J* = 5.5 Hz, 1H), 7.58 (s, 1H), 7.57 (s, 1H), 7.45–7.34 (m, 3H), 7.16 (d, *J* = 8.2 Hz, 1H), 3.89 (s, 3H), 3.86 (s, 3H); ^13^C NMR (100 MHz, CDCl_3_) *δ* 194.98, 159.68, 159.33, 154.03, 139.35, 138.32, 132.64, 129.41, 128.66, 127.93, 124.31, 124.00, 122.73, 120.26, 114.48, 103.22, 55.53, 55.50; IR (KBr film) 3448, 2923, 1660, 1622, 1593, 1452, 1315, 1283, 1263, 1235, 1038, 869, 848, 646 cm^−1^; HRMS (ESI) *m*/*z* calcd for C_18_H_16_NO_3_ [M + H]^+^: 294.1130, found: 294.1136.

#### (7-Methoxyisoquinolin-1-yl)(4-methoxyphenyl)methanone (6l)

White crystals, mp 101–103 °C. ^1^H NMR (400 MHz, CDCl_3_) *δ* 8.49 (d, *J* = 5.5 Hz, 1H), 7.96 (d, *J* = 8.9 Hz, 2H), 7.81 (d, *J* = 9.0 Hz, 1H), 7.73 (d, *J* = 5.5 Hz, 1H), 7.54 (s, 1H), 7.38 (d, *J* = 9.0 Hz, 1H), 6.96 (d, *J* = 8.9 Hz, 2H), 3.88 (s, 3H), 3.87 (s, 3H); ^13^C NMR (100 MHz, CDCl_3_) *δ* 193.74, 164.02, 159.15, 154.69, 139.33, 133.27 (2C), 132.58, 129.76, 128.60, 127.82, 124.19, 122.35, 113.76 (2C), 103.35, 55.56, 55.51. IR (KBr film) 2926, 2837, 1652, 1622, 1601, 1506, 1427, 1251, 1215, 1185, 1166, 1044, 843, 613 cm^−1^. HRMS (ESI) *m*/*z* calcd for C_18_H_16_NO_3_ [M + H]^+^: 294.1130, found: 294.1127.

#### (3,4-Dimethoxyphenyl)(7-methoxyisoquinolin-1-yl)methanone (6m)

White crystals, mp 150–152 °C. ^1^H NMR (400 MHz, CDCl_3_) *δ* 8.49 (d, *J* = 5.4 Hz, 1H), 7.82 (d, *J* = 9.0 Hz, 1H), 7.74 (d, *J* = 5.4 Hz, 1H), 7.72 (s, 1H), 7.52 (s, 1H), 7.44–7.38 (m, 2H), 6.87 (d, *J* = 8.5 Hz, 1H), 3.97 (s, 3H), 3.95 (s, 3H), 3.88 (s, 3H); ^13^C NMR (100 MHz, CDCl_3_) *δ* 193.76, 159.17, 154.68, 153.92, 149.11, 139.27, 132.57, 129.87, 128.60, 127.86, 126.92, 124.23, 122.35, 111.89, 109.99, 103.37, 56.14, 56.04, 55.52; IR (KBr film) 2998, 2931, 1653, 1597, 1585, 1516, 1265, 1232, 1143, 1027, 847, 762, 641 cm^−1^. HRMS (ESI) *m*/*z* calcd for C_19_H_18_NO_4_ [M + H]^+^: 324.1236, found: 324.1233.

#### (7-Methoxyisoquinolin-1-yl)(phenyl)methanone (6n)

White crystals, mp 120–122 °C. ^1^H NMR (400 MHz, CDCl_3_) *δ* 8.50 (d, *J* = 5.4 Hz, 1H), 7.96 (d, *J* = 8.4 Hz, 2H), 7.82 (d, *J* = 9.0 Hz, 1H), 7.75 (d, *J* = 5.4 Hz, 1H), 7.62 (t, *J* = 8.0 Hz, 1H), 7.61 (s, 1H), 7.49 (dd, *J*_1_ = 8.0 Hz, *J*_2_ = 8.4 Hz, 2H), 7.40 (d, *J* = 9.0 Hz, 1H), 3.89 (s, 3H); ^13^C NMR (100 MHz, CDCl_3_) *δ* 195.13, 159.37, 153.95, 139.32, 137.04, 133.50, 132.68, 130.85 (2C), 128.65 (2C), 128.42, 128.00, 124.31, 122.75, 103.29, 55.53; IR (KBr film) 2978, 2923, 1661, 1624, 1502, 1455, 1317, 1247, 1216, 1168, 851, 715, 646 cm^−1^. HRMS (ESI) *m*/*z* calcd for C_17_H_14_NO_2_ [M + H]^+^: 264.1025, found: 264.1018.

#### (Isoquinolin-1-yl)(3-methoxyphenyl)methanone (6o)

White crystals, mp 62–63 °C. ^1^H NMR (400 MHz, CDCl_3_) *δ* 8.60 (d, *J* = 5.6 Hz, 1H), 8.19 (d, *J* = 8.5 Hz, 1H), 7.93 (d, *J* = 8.4 Hz, 1H), 7.82 (d, *J* = 5.6 Hz, 1H), 7.75 (dd, *J*_1_ = 8.2 Hz, *J*_2_ = 8.4 Hz, 1H), 7.63 (dd, *J*_1_ = 8.2 Hz, *J*_2_ = 8.5 Hz, 1H), 7.58 (s, 1H), 7.41 (d, *J* = 7.6 Hz, 1H), 7.35 (dd, *J*_1_ = 7.6 Hz, *J*_2_ = 8.0 Hz, 1H), 7.16 (d, *J* = 8.0 Hz, 1H), 3.86 (s, 3H); ^13^C NMR (100 MHz, CDCl_3_) *δ* 194.61, 159.73, 156.49, 141.16, 137.93, 136.67, 130.78, 129.50, 128.36, 127.14, 126.35, 126.12, 123.99, 122.63, 120.44, 114.33, 55.50; IR (KBr film) 3005, 2937, 1665, 1594, 1463, 1281, 1266, 1147, 1039, 834, 769, 637 cm^−1^; HRMS (ESI) *m*/*z* calcd for C_17_H_14_NO_2_ [M + H]^+^: 264.1025, found: 264.1030.

#### (Isoquinolin-1-yl)(4-methoxyphenyl)methanone (6p)

White crystals, mp 67–69 °C. ^1^H NMR (400 MHz, CDCl_3_) *δ* 8.60 (d, *J* = 5.7 Hz, 1H), 8.17 (d, *J* = 8.5 Hz, 1H), 7.95 (d, *J* = 8.8 Hz, 2H), 7.94 (d, *J* = 8.0 Hz, 1H), 7.81 (d, *J* = 5.7 Hz, 1H), 7.75 (dd, *J*_1_ = 8.3 Hz, *J*_2_ = 8.5 Hz, 1H), 7.61 (dd, *J*_1_ = 8.0 Hz, *J*_2_ = 8.3 Hz, 1H), 6.95 (d, *J* = 8.8 Hz, 2H), 3.88 (s, 3H); ^13^C NMR (100 MHz, CDCl_3_) *δ* 193.40, 164.16, 157.08, 141.16, 136.67, 133.19 (2C), 130.72, 129.52, 128.19, 127.08, 126.32, 126.30, 122.30, 113.83 (2C), 55.58; IR (KBr film) 3008, 2936, 1651, 1600, 1576, 1412, 1249, 1154, 1022, 828, 745 cm^−1^.

#### (3,4-Dimethoxyphenyl)(isoquinolin-1-yl)methanone (6q)

White crystals, mp 145–146 °C. ^1^H NMR (400 MHz, CDCl_3_) *δ* 8.60 (d, *J* = 5.7 Hz, 1H), 8.16 (d, *J* = 8.5 Hz, 1H), 7.93 (d, *J* = 8.1 Hz, 1H), 7.81 (d, *J* = 5.7 Hz, 1H), 7.76 (dd, *J*_1_ = 8.3 Hz, *J*_2_ = 8.5 Hz, 1H), 7.74 (s, 1H), 7.62 (dd, *J*_1_ = 8.1 Hz, *J*_2_ = 8.3 Hz, 1H), 7.36 (d, *J* = 8.4 Hz, 1H), 6.84 (d, *J* = 8.4 Hz, 1H), 3.97 (s, 3H), 3.94 (s, 3H); ^13^C NMR (100 MHz, CDCl_3_) *δ* 193.45, 157.03, 154.04, 149.18, 141.12, 136.66, 130.76, 129.62, 128.20, 127.08, 127.02, 126.36, 126.29, 122.31, 111.52, 109.97, 56.17, 56.08; IR (KBr film) 3002, 2938, 1660, 1582, 1510, 1410, 1266, 1150, 1134, 1019, 825, 752 cm^−1^; HRMS (ESI) *m*/*z* calcd for C_18_H_16_NO_3_ [M + H]^+^: 294.1130, found: 294.1131.

#### (Isoquinolin-1-yl)(phenyl)methanone (6r)

White crystals, mp 74–75 °C. ^1^H NMR (400 MHz, CDCl_3_) *δ* 8.61 (d, *J* = 5.6 Hz, 1H), 8.22 (d, *J* = 8.5 Hz, 1H), 8.00–7.91 (m, 3H), 7.81 (d, *J* = 5.6 Hz, 1H), 7.74 (dd, *J*_1_ = 8.2 Hz, *J*_2_ = 8.5 Hz, 1H), 7.64–7.58 (m, 2H), 7.48 (dd, *J*_1_ = 7.8 Hz, *J*_2_ = 8.0 Hz, 2H); ^13^C NMR (100 MHz, CDCl_3_) *δ* 194.81, 156.44, 141.18, 136.71, 136.63, 133.73, 130.78 (2C), 130.74, 128.51, 128.37 (2C), 127.15, 126.42, 126.16, 122.65; IR (KBr film) 2978, 2922, 1663, 1596, 1449, 1402, 1335, 1317, 1279, 1252, 1153, 924, 709, 678, 639 cm^−1^.

#### (2-Bromophenyl)([1,3]dioxolo[4,5-*g*]isoquinolin-5-yl)metha-none (6s)

White crystals, mp 180–181 °C. ^1^H NMR (400 MHz, CDCl_3_) *δ* 8.38 (d, *J* = 5.4 Hz, 1H), 8.20 (s, 1H), 7.65–7.57 (m, 3H), 7.46 (dd, *J*_1_ = 7.7 Hz, *J*_2_ = 7.9 Hz, 1H), 7.36 (dd, *J*_1_ = 7.9 Hz, *J*_2_ = 8.1 Hz, 1H), 7.15 (s, 1H), 6.15 (s, 2H); ^13^C NMR (100 MHz, CDCl_3_) *δ* 197.22, 151.43, 151.04, 150.38, 141.64, 140.61, 135.97, 133.08, 131.88, 130.72, 127.35, 124.68, 123.63, 120.46, 102.78, 102.53, 102.00; IR (KBr film) 2975, 2908, 1679, 1576, 1497, 1460, 1317, 1261, 1208, 1037, 951, 867, 723, 631 cm^−1^; HRMS (ESI) *m*/*z* calcd for C_17_H_11_NO_3_Br [M + H]^+^: 355.9922, found: 355.9917.

#### (4-Methoxyphenyl)(5,6,7-trimethoxyisoquinolin-1-yl)methan-one (6t)

White crystals, mp 154–156 °C. ^1^H NMR (400 MHz, CDCl_3_) *δ* 8.48 (d, *J* = 5.5 Hz, 1H), 8.01 (d, *J* = 5.5 Hz, 1H), 7.94 (d, *J* = 8.3 Hz, 2H), 7.36 (s, 1H), 6.96 (d, *J* = 8.3 Hz, 2H), 4.08 (s, 3H), 4.03 (s, 3H), 3.93 (s, 3H), 3.88 (s, 3H); ^13^C NMR (100 MHz, CDCl_3_) *δ* 193.72, 164.04, 154.58, 154.01, 146.71, 144.30, 139.44, 133.26 (2C), 129.73, 129.21, 123.77, 116.58, 113.75 (2C), 100.36, 61.64, 61.23, 56.10, 55.55; IR (KBr film) 2944, 2839, 1651, 1602, 1479, 1327, 1280, 1256, 1163, 1125, 1028, 935, 823, 702 cm^−1^. HRMS (ESI) *m*/*z* calcd for C_20_H_20_NO_5_ [M + H]^+^: 354.1341, found: 354.1336.

### Preparation of anilines 7a–f

Nitro compound 6 (∼2 mmol) was dissolved in a mixed solvent of EtOH (18 mL) and H_2_O (2 mL). Iron powder (1.130 g, 20.23 mmol) and acetic acid (3.610 g, 60.12 mmol) were added into the solution. The mixture was then heated and stirred under N_2_ at 70 °C for 3–5 h. After the reaction was complete (checked by TLC, eluent: EtOAc/hexane = 1 : 2), the mixture was cooled down to room temperature and then filtered. The cake was washed twice with EtOH (2 × 10 mL). The filtrate was concentrated under vacuum to give a residue, which was dissolved in CH_2_Cl_2_ (30 mL). An aqueous solution of Na_3_PO_4_ (20 mL, 10% w/w) was added, the mixture was vigorously stirred for 5 min. Two phases were separated, and the aqueous phase was extracted again with CH_2_Cl_2_ (30 mL). The organic extracts were combined, and dried over anhydrous MgSO_4_. Solvent was removed under vacuum to give crude product as pale yellow solid, which was purified by flash chromatography (eluent: EtOAc/hexane = 1 : 2). Anilines 7a–f were thus obtained in 85%, 85%, 86%, 93%, 90% and 91% yields, respectively. Characterization data of compounds 7a–f are as follows:

#### (2-Aminophenyl)([1,3]dioxolo[4,5-*g*]isoquinolin-5-yl)metha-none (7a)

Pale yellow crystals, mp 246–247 °C. ^1^H NMR (400 MHz, DMSO-*d*_6_) *δ* 8.37 (d, *J* = 5.6 Hz, 1H), 7.78 (d, *J* = 5.6 Hz, 1H), 7.56 (br. s, 2H, N*H*_2_), 7.47 (s, 1H), 7.29 (dd, *J*_1_ = 8.2 Hz, *J*_2_ = 8.5 Hz, 1H), 7.03 (s, 1H), 6.92–6.87 (m, 2H), 6.37 (dd, *J*_1_ = 8.0 Hz, *J*_2_ = 8.3 Hz, 1H), 6.22 (s, 2H); ^13^C NMR (100 MHz, DMSO-*d*_6_) *δ* 196.49, 156.03, 152.65, 150.85, 148.81, 140.43, 135.06, 134.73, 134.12, 122.04, 120.89, 116.90, 115.83, 114.16, 102.77, 102.21, 100.59; IR (KBr film) 3421, 3124, 3078, 2918, 1684, 1576, 1518, 1460, 1348, 1314, 1275, 1260, 1214, 1036, 946, 867, 788, 706, 636 cm^−1^; HRMS (ESI) *m*/*z* calcd for C_17_H_13_N_2_O_3_ [M + H]^+^: 293.0926, found: 293.0919.

#### (2-Amino-4,5-dimethoxy-phenyl)([1,3]dioxolo[4,5-*g*]isoqui-nolin-5-yl)methanone (7b)

Pale yellow crystals, mp 182–183 °C. ^1^H NMR (400 MHz, DMSO-*d*_6_) *δ* 8.37 (d, *J* = 5.6 Hz, 1H), 7.74 (d, *J* = 5.6 Hz, 1H), 7.56 (br. s, 2H, N*H*_2_), 7.41 (s, 1H), 7.05 (s, 1H), 6.48 (s, 1H), 6.40 (s, 1H), 6.19 (s, 2H), 3.80 (s, 3H), 3.43 (s, 3H); ^13^C NMR (100 MHz, DMSO-*d*_6_) *δ* 193.52, 156.42, 156.34, 150.85, 150.76, 148.67, 140.36, 138.72, 134.74, 122.05, 120.74, 115.72, 108.04, 102.75, 102.17, 100.79, 98.61, 56.07, 55.41; IR (KBr film) 3415, 3310, 2916, 1645, 1585, 1545, 1507, 1465, 1403, 1264, 1221, 1204, 1150, 1110, 1037, 948, 873, 854, 734, 675 cm^−1^; HRMS (ESI) *m*/*z* calcd for C_19_H_17_N_2_O_5_ [M + H]^+^: 353.1137, found: 353.1142.

#### (6-Aminobenzo[*d*][1,3]dioxol-5-yl)([1,3]dioxolo[4,5-*g*]iso-quinolin-5-yl)methanone (7c)

Pale yellow crystals, mp 240–241 °C. ^1^H NMR (400 MHz, DMSO-*d*_6_) *δ* 8.37 (d, *J* = 5.6 Hz, 1H), 7.84 (br. s, 2H, N*H*_2_), 7.75 (d, *J* = 5.6 Hz, 1H), 7.44 (s, 1H), 7.02 (s, 1H), 6.46 (s, 1H), 6.21 (s, 2H), 6.19 (s, 1H), 5.91 (s, 2H); ^13^C NMR (100 MHz, DMSO-*d*_6_) *δ* 193.22, 156.33, 153.63, 152.51, 150.77, 148.66, 140.41, 137.27, 134.70, 121.90, 120.70, 109.29, 108.08, 102.75, 102.17, 101.29, 100.70, 95.69; IR (KBr film) 3393, 3286, 2890, 1684, 1648, 1565, 1500, 1462, 1421, 1392, 1270, 1230, 1207, 1091, 1035, 944, 855, 839, 778, 669 cm^−1^; HRMS (ESI) *m*/*z* calcd for C_18_H_13_N_2_O_5_ [M + H]^+^: 337.0824, found: 337.0826.

#### (2-Aminophenyl)(6,7-dimethoxyisoquinolin-1-yl)methanone (7d)

Pale yellow crystals, mp 170–171 °C. ^1^H NMR (400 MHz, CDCl_3_) *δ* 8.42 (d, *J* = 5.6 Hz, 1H), 7.57 (d, *J* = 5.6 Hz, 1H), 7.25 (s, 1H), 7.25–7.18 (m, 2H), 7.10 (s, 1H), 6.72 (d, *J* = 8.2 Hz, 1H), 6.60 (br. s, 2H, N*H*_2_), 6.46 (dd, *J*_1_ = 8.0 Hz, *J*_2_ = 8.2 Hz, 1H), 3.99 (s, 3H), 3.88 (s, 3H); ^13^C NMR (100 MHz, CDCl_3_) *δ* 197.64, 155.57, 153.14, 152.11, 150.72, 140.34, 135.20, 134.79, 133.77, 122.06, 120.45, 117.46, 117.06, 115.57, 104.95, 103.94, 56.10, 56.04; IR (KBr film) 3412, 3302, 2967, 2844, 1672, 1645, 1477, 1282, 1212, 1127, 1090, 938, 854, 735, 674 cm^−1^; HRMS (ESI) *m*/*z* calcd for C_18_H_17_N_2_O_3_ [M + H]^+^: 309.1239, found: 309.1236.

#### (2-Amino-4,5-dimethoxyphenyl)(6,7-dimethoxyisoquinolin-1-yl)methanone (7e)

Pale yellow crystals, mp 156–157 °C. ^1^H NMR (400 MHz, CDCl_3_) *δ* 8.44 (d, *J* = 5.5 Hz, 1H), 7.59 (d, *J* = 5.5 Hz, 1H), 7.33 (s, 1H), 7.13 (s, 1H), 6.68 (s, 1H), 6.56 (br. s, 2H, N*H*_2_), 6.20 (s, 1H), 4.04 (s, 3H), 3.93 (s, 3H), 3.89 (s, 3H), 3.50 (s, 3H); ^13^C NMR (100 MHz, CDCl_3_) *δ* 195.00, 156.42, 155.80, 153.14, 150.65, 149.96, 140.32, 140.01, 133.86, 122.09, 120.34, 115.97, 109.84, 104.88, 104.21, 98.82, 56.38, 56.12, 56.10, 55.93; IR (KBr film) 3421, 3312, 2982, 2834, 1658, 1601, 1470, 1285, 1252, 1203, 1143, 1027, 937, 828, 689 cm^−1^; HRMS (ESI) *m*/*z* calcd for C_20_H_21_N_2_O_5_ [M + H]^+^: 369.1450, found: 369.1449.

#### (2-Aminophenyl)(5,6,7-trimethoxyisoquinolin-1-yl)methanone (7f)

Pale yellow crystals, mp 105–106 °C. ^1^H NMR (400 MHz, CDCl_3_) *δ* 8.47 (d, *J* = 5.7 Hz, 1H), 7.94 (d, *J* = 5.7 Hz, 1H), 7.27 (dd, *J*_1_ = 8.1 Hz, *J*_2_ = 7.8 Hz, 1H), 7.20 (d, *J* = 8.1 Hz, 1H), 7.10 (s, 1H), 6.73 (d, *J* = 8.2 Hz, 1H), 6.55 (br. s, 2H, N*H*_2_), 6.50 (dd, *J*_1_ = 8.2 Hz, *J*_2_ = 7.8 Hz, 1H), 4.07 (s, 3H), 4.02 (s, 3H), 3.89 (s, 3H); ^13^C NMR (100 MHz, CDCl_3_) *δ* 197.48, 155.80, 154.30, 152.05, 146.79, 144.28, 139.82, 135.27, 134.87, 129.00, 123.14, 117.48, 117.04, 115.73, 115.69, 100.23, 61.62, 61.23, 56.10; IR (KBr, film) 3457, 3310, 2958, 2922, 2851, 1668, 1619, 1584, 1547, 1475, 1262, 1203, 1157, 1122, 1049, 940, 749, 657 cm^−1^; HRMS (ESI): calcd for C_19_H_19_N_2_O_4_ [M + H]^+^: 339.1345, found: 339.1337.

### Preparation of oxoaporphines 1a–f

Aniline 7 (∼1 mmol) was dissolved in an aqueous solution (5 mL, 10% w/w) of sulfuric acid. The solution was cooled to 0–8 °C with an ice bath. A freshly prepared aqueous solution (1 mL, 1 M) of sodium nitrite was dropwise added over 5 min. Stirring was continued at 0–8 °C for 15 min to give a clear pale orange aqueous solution of diazonium salt, which was immediately used below. Copper powder (0.640 g, 10.07 mmol) was added into an aqueous solution (5 mL, 10% w/w) of sulfuric acid. The suspension was warmed and stirred at 60 °C. The above aqueous solution of diazonium salt was dropwise added over 10 min. The mixture was further stirred at 60 °C for 2–4 h. After the reaction was complete (checked by TLC, eluent: EtOAc/hexane = 2 : 1), the mixture was cooled to room temperature. CH_2_Cl_2_ (30 mL) and concentrated ammonia (10 mL, 25% w/w) were added, and the mixture was then vigorously stirred for 5 min. Two phases were separated, and the aqueous phase was extracted again with CH_2_Cl_2_ (30 mL). The organic extracts were combined, and dried over anhydrous MgSO_4_. The organic solution was concentrated under vacuum to give crude product, which was purified by flash chromatography (eluent: EtOAc/CH_2_Cl_2_ = 1 : 4). Compounds 1a–f were thus obtained in 86%, 77%, 82%, 80%, 79% and 81% yields, respectively. Characterization data of oxoaporphines 1a–f are as follows:

#### Liriodenine (1a)

Pale yellow crystals, mp 283–284 °C (lit.^3*d*^ mp 282 °C). ^1^H NMR (400 MHz, CDCl_3_-TFA) *δ* 8.78 (d, *J* = 5.9 Hz, 1H), 8.66 (d, *J* = 8.1 Hz, 1H), 8.43 (d, *J* = 8.0 Hz, 1H), 8.36 (d, *J* = 5.9 Hz, 1H), 7.93 (dd, *J*_1_ = 7.8 Hz, *J*_2_ = 8.1 Hz, 1H), 7.67 (dd, *J*_1_ = 7.8 Hz, *J*_2_ = 8.0 Hz, 1H), 7.48 (s, 1H), 6.63 (s, 2H); ^13^C NMR (100 MHz, CDCl_3_-TFA) *δ* 176.51, 158.50, 152.03, 143.26, 137.16, 134.99, 133.94, 132.15, 130.37, 129.11, 128.27, 128.08, 126.79, 122.36, 108.14, 105.20, 103.66; IR (KBr film) 3034, 2920, 1660, 1597, 1575, 1469, 1486, 1421, 1309, 1260, 1229, 1206, 1125, 1041, 1014, 958, 869, 778, 689, 568 cm^−1^; HRMS (ESI) *m*/*z* calcd for C_17_H_10_NO_3_ [M + H]^+^: 276.0661, found: 276.0665.

#### Dicentrinone (1b)

Pale yellow crystals, mp 292–293 °C (lit.^4*b*^ mp 293–295 °C). ^1^H NMR (400 MHz, CDCl_3_-TFA) *δ* 8.74 (d, *J* = 5.6 Hz, 1H), 8.33 (d, *J* = 5.6 Hz, 1H), 8.05 (s, 1H), 7.82 (s, 1H), 7.43 (s, 1H), 6.63 (s, 2H), 4.10 (s, 3H), 4.04 (s, 3H); ^13^C NMR (100 MHz, CDCl_3_-TFA) *δ* 174.96, 158.63, 157.13, 152.03, 150.56, 143.27, 135.07, 133.57, 129.04, 126.63, 122.59, 121.78, 109.71, 109.64, 108.06, 105.40, 103.58, 56.46, 56.16; IR (KBr film) 3002, 2918, 2839, 1640, 1593, 1577, 1513, 1476, 1453, 1425, 1368, 1344, 1307, 1276, 1253, 1217, 1136, 1056, 1001, 966, 855, 777, 676, 636, 577 cm^−1^; HRMS (ESI) *m*/*z* calcd for C_19_H_14_NO_5_ [M + H]^+^: 336.0872, found: 336.0870.

#### Cassameridine (1c)

Pale yellow crystals, mp 301–302 °C (lit.^5*a*^ mp 300 °C). ^1^H NMR (400 MHz, CDCl_3_-TFA) *δ* 8.72 (d, *J* = 6.2 Hz, 1H), 8.35 (d, *J* = 6.2 Hz, 1H), 8.11 (s, 1H), 7.76 (s, 1H), 7.45 (s, 1H), 6.61 (s, 2H), 6.23 (s, 2H); ^13^C NMR (100 MHz, CDCl_3_-TFA) *δ* 174.76, 158.64, 156.46, 152.30, 150.01, 143.18, 134.79, 133.71, 130.79, 126.58, 124.25, 121.60, 108.03, 107.52, 107.12, 105.34, 103.55, 103.54; IR (KBr film) 3029, 2917, 2848, 1648, 1611, 1572, 1503, 1482, 1467, 1444, 1376, 1294, 1268, 1240, 1123, 1101, 1079, 1040, 965, 899, 818, 604; HRMS (ESI) *m*/*z* calcd for C_18_H_10_NO_5_ [M + H]^+^: 320.0559, found: 320.0560.

#### Lysicamine (1d)

Pale yellow crystals, mp 206–207 °C (lit.^26^ mp 207–208 °C). ^1^H NMR (400 MHz, CDCl_3_) *δ* 9.17 (d, *J* = 8.2 Hz, 1H), 8.90 (d, *J* = 5.3 Hz, 1H), 8.59 (d, *J* = 7.8 Hz, 1H), 7.80 (d, *J* = 5.3 Hz, 1H), 7.78 (dd, *J*_1_ = 7.8 Hz, *J*_2_ = 8.0 Hz, 1H), 7.57 (dd, *J*_1_ = 8.0 Hz, *J*_2_ = 8.2 Hz, 1H), 7.22 (s, 1H), 4.10 (s, 3H), 4.02 (s, 3H); ^13^C NMR (100 MHz, CDCl_3_) *δ* 182.71, 156.85, 152.08, 152.05, 145.39, 145.05, 135.50, 134.34, 132.11, 128.90, 128.80, 128.45, 123.58, 122.11, 119.84, 106.48, 60.67, 56.23; IR (KBr film) 2968, 2920, 2837, 1662, 1610, 1552, 1512, 1465, 1415, 1375, 1260, 1235, 1110, 1079, 1041, 962, 887, 792, 605; HRMS (ESI) *m*/*z* calcd for C_18_H_14_NO_3_ [M + H]^+^: 292.0974, found: 292.0970.

#### Oxoglaucine (1e)

Pale yellow crystals, mp 225–226 °C (lit.^27^ mp 227–229 °C). ^1^H NMR (400 MHz, CDCl_3_) *δ* 8.82 (d, *J* = 5.2 Hz, 1H), 8.72 (s, 1H), 7.95 (s, 1H), 7.69 (d, *J* = 5.2 Hz, 1H), 7.11 (s, 1H), 4.06 (s, 3H), 4.04 (s, 3H), 4.03 (s, 3H), 3.99 (s, 3H); ^13^C NMR (100 MHz, CDCl_3_) *δ* 181.28, 156.60, 153.65, 150.91, 149.39, 145.38, 144.81, 135.25, 129.05, 126.76, 123.30, 121.53, 119.73, 110.13, 109.67, 105.98, 60.57, 56.18, 56.17, 55.98; IR (KBr film) 2979, 2924, 2841, 1662, 1600, 1554, 1501, 1463, 1403, 1378, 1292, 1266, 1246, 1217, 1100, 1081, 1037, 975, 890, 827, 739, 624; HRMS (ESI) *m*/*z* calcd for C_20_H_18_NO_5_ [M + H]^+^: 352.1185, found: 352.1187.

#### *O*-Methylmoschatoline (1f)

Pale yellow crystals, mp 184–185 °C (lit.^10*b*^ mp 186–188 °C). ^1^H NMR (400 MHz, CDCl_3_) *δ* 9.00 (d, *J* = 8.2 Hz, 1H), 8.86 (d, *J* = 5.2 Hz, 1H), 8.46 (d, *J* = 7.7 Hz, 1H), 8.11 (d, *J* = 5.2 Hz, 1H), 7.64 (dd, *J*_1_ = 8.2 Hz, *J*_2_ = 7.9 Hz, 1H), 7.42 (dd, *J*_1_ = 7.7 Hz, *J*_2_ = 7.9 Hz, 1H), 4.11 (s, 3H), 4.03 (s, 3H), 4.00 (s, 3H); ^13^C NMR (100 MHz, CDCl_3_) *δ* 182.59, 156.48, 148.45, 147.25, 145.43, 144.52, 134.51, 134.34, 131.38, 131.01, 128.85, 128.08, 127.64, 122.77, 119.12, 115.56, 61.81, 61.46, 60.99; IR (KBr, film) 2956, 2923, 2853, 1660, 1579, 1466, 1393, 1311, 1261, 1205, 1092, 970, 800, 723, 612 cm^−1^; HRMS (ESI): calcd for C_19_H_16_NO_4_ [M + H]^+^: 322.1079, found: 322.1072.

## Conflicts of interest

There are no conflicts to declare.

## Supplementary Material

RA-008-C8RA05338C-s001
