# Supplementary material for: Novel total syntheses of oxoaporphine alkaloids enabled by mild Cu-catalyzed tandem oxidation/aromatization of 1-Bn-DHIQs
Source: RSC Adv. 2018 Aug 14;8(51):28997–9007. doi: 10.1039/c8ra05338c (PMC9084380; doi:10.1039/c8ra05338c)

Electronic Supplementary Material (ESI) for RSC Advances.
This journal is © The Royal Society of Chemistry 2018

Supporting Information

Novel Total Syntheses of Oxoaporphine Alkaloids Enabled by Mild Cu-Catalyzed Tandem Oxidation/Aromatization of 1-Bn-DHIQs

Bo Zheng, Hui-Ya Qu, Tian-Zhuo Meng, Xia Lu, Jie Zheng, Yun-Gang He, Qi-Qi Fan, Xiao-Xin Shi*

Shanghai Key Laboratory of Chemical Biology and Department of Pharmaceutical Engineering, School of Pharmacy, East China University of Science and Technology, 130 Mei-Long Road, Shanghai 200237, P. R. China
E-mail: xxshi@ecust.edu.cn

Contents

^{1}H and ^{13}C NMR Spectra of All Compounds...S2-S45

^{1}H and ^{13}C NMR Spectra of All Compounds:

^{1}H NMR ($CDCl_3$, 400 MHz) spectrum of compound **2a**:

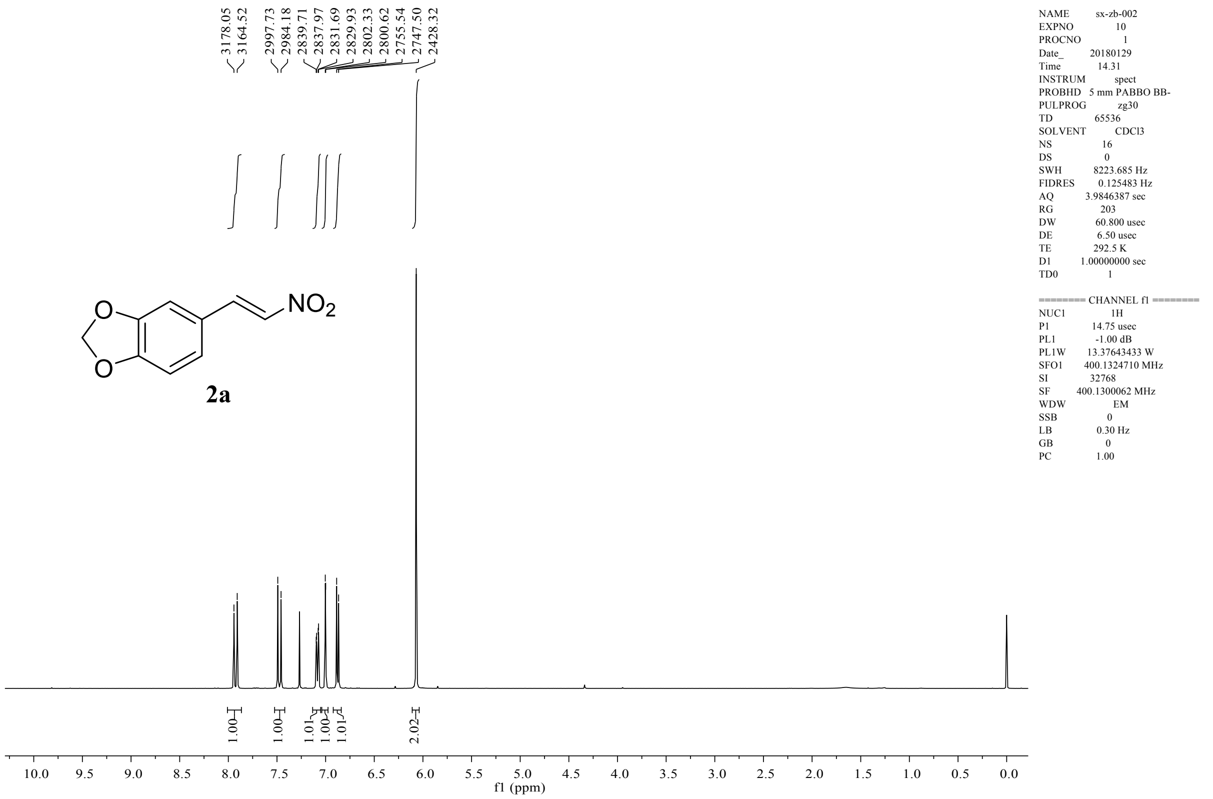

^{13}C NMR ($CDCl_3$, 100 MHz) spectrum of compound **2a**:

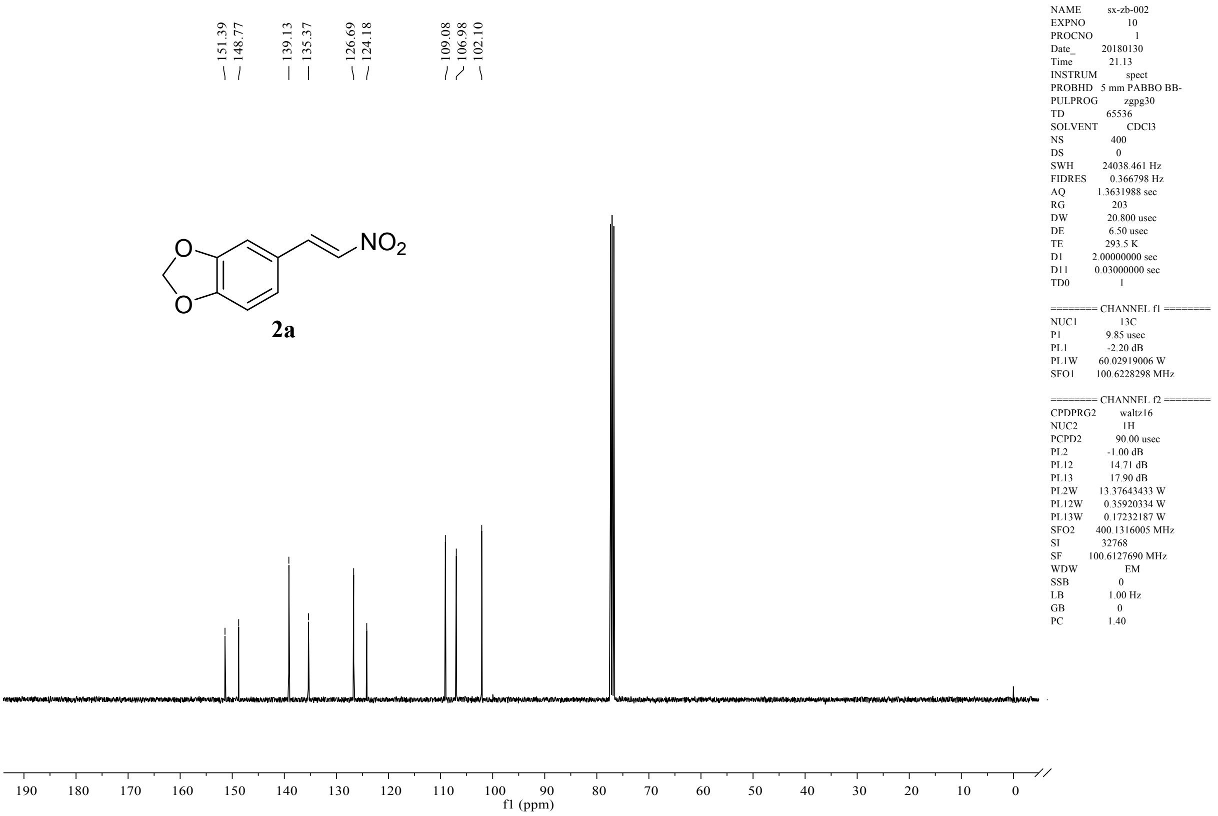

^{1}H NMR ($CDCl_3$, 400 MHz) spectrum of compound **2b**:

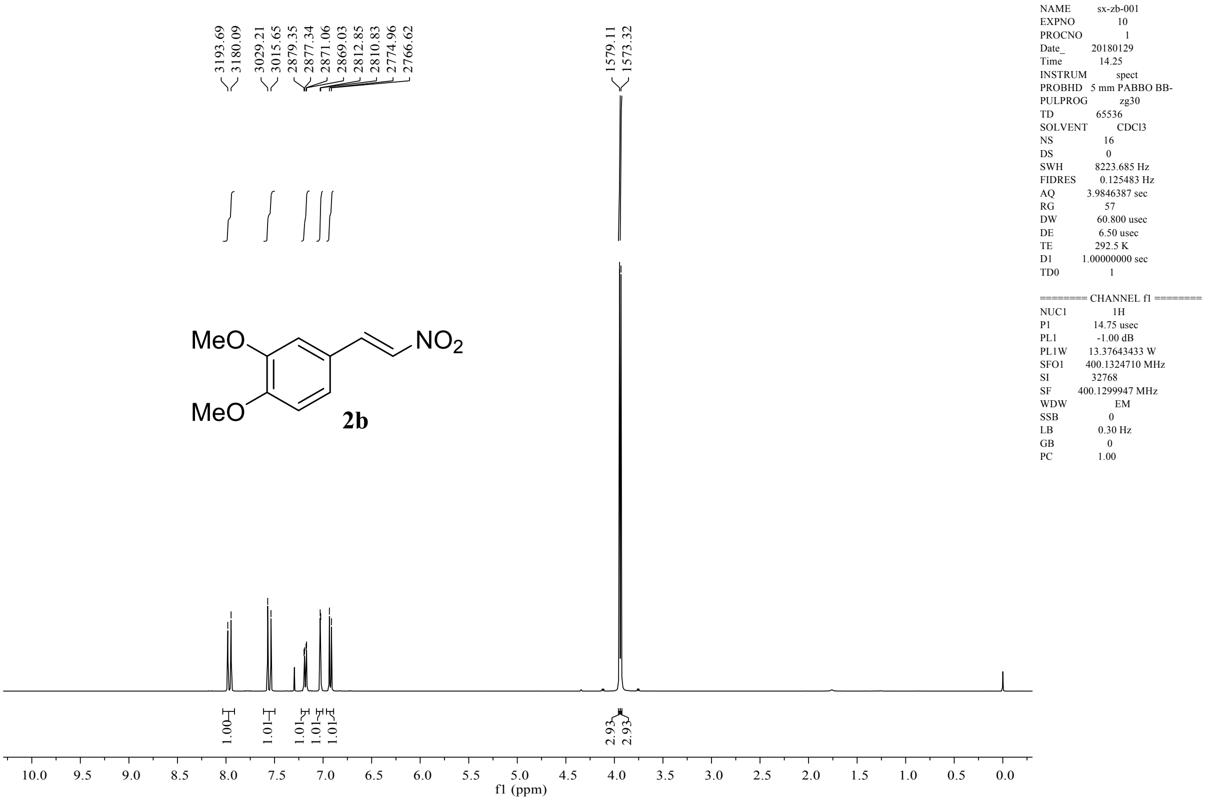

^{13}C NMR ($CDCl_3$, 100 MHz) spectrum of compound **2b**:

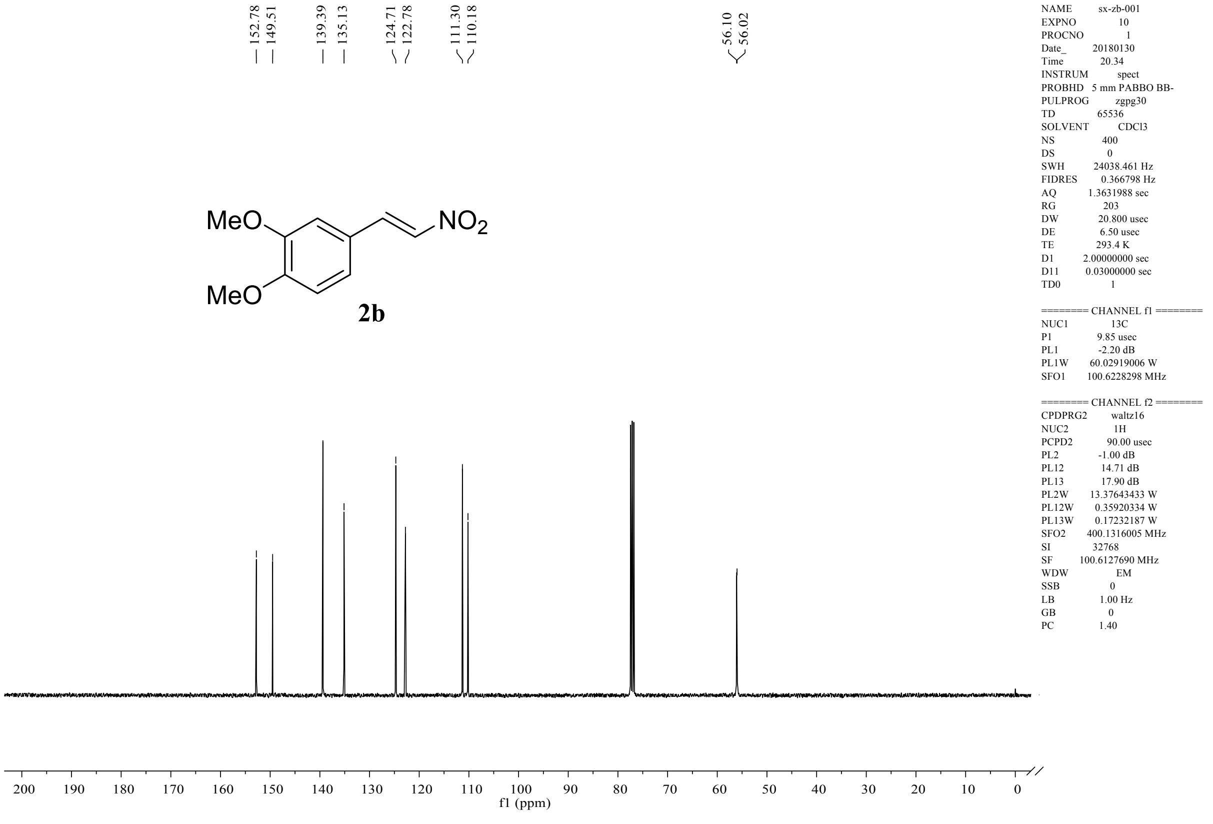

^{1}H NMR ($CDCl_3$, 400 MHz) spectrum of compound **2c**:

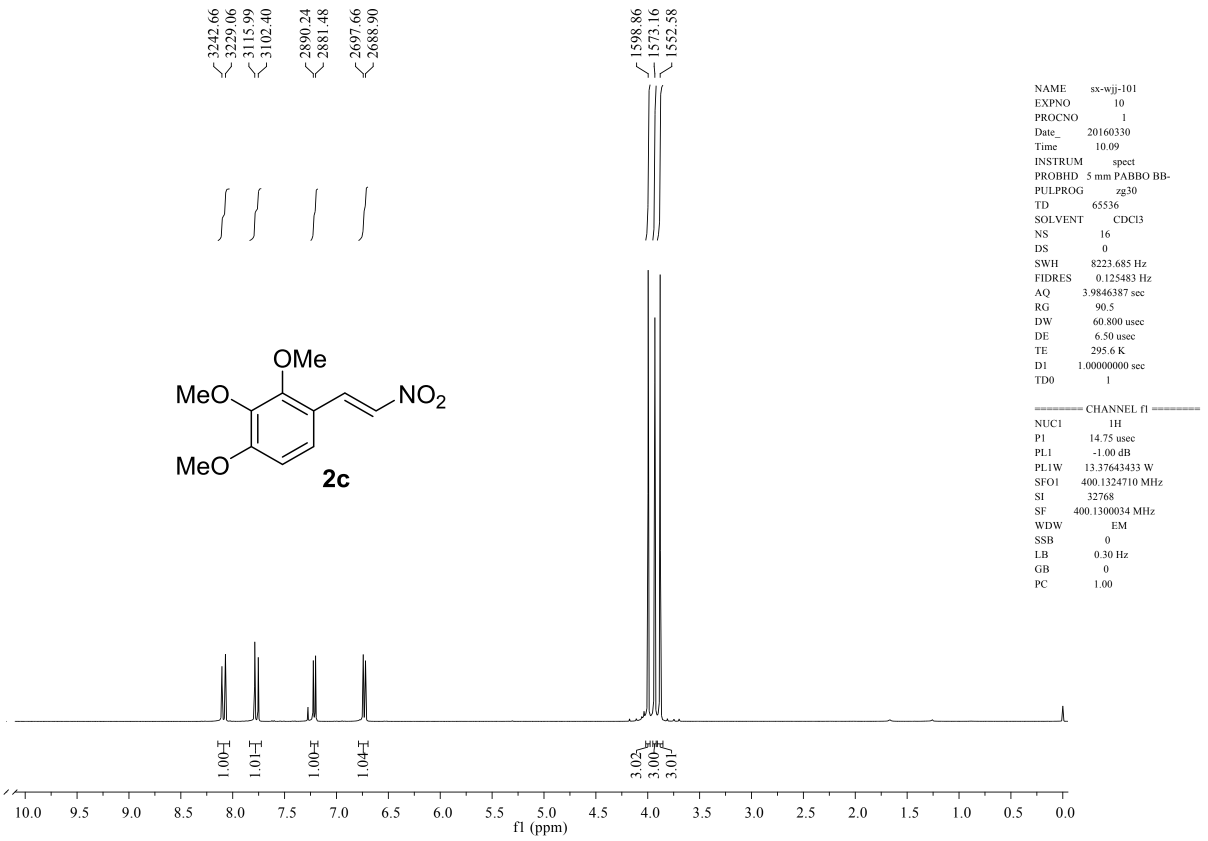

^{13}C NMR ($CDCl_3$, 100 MHz) spectrum of compound **2c**:

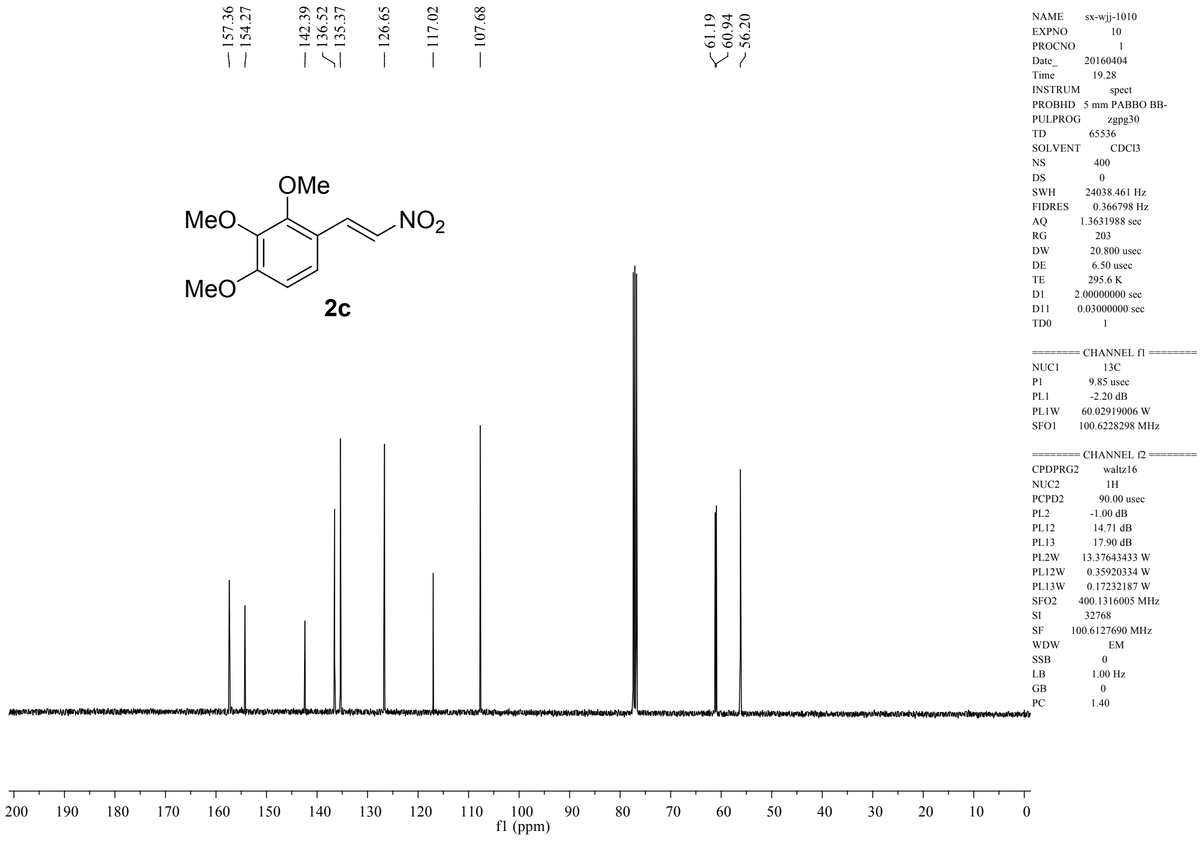

^{1}H NMR ($CDCl_3$, 400 MHz) spectrum of compound **3a**:

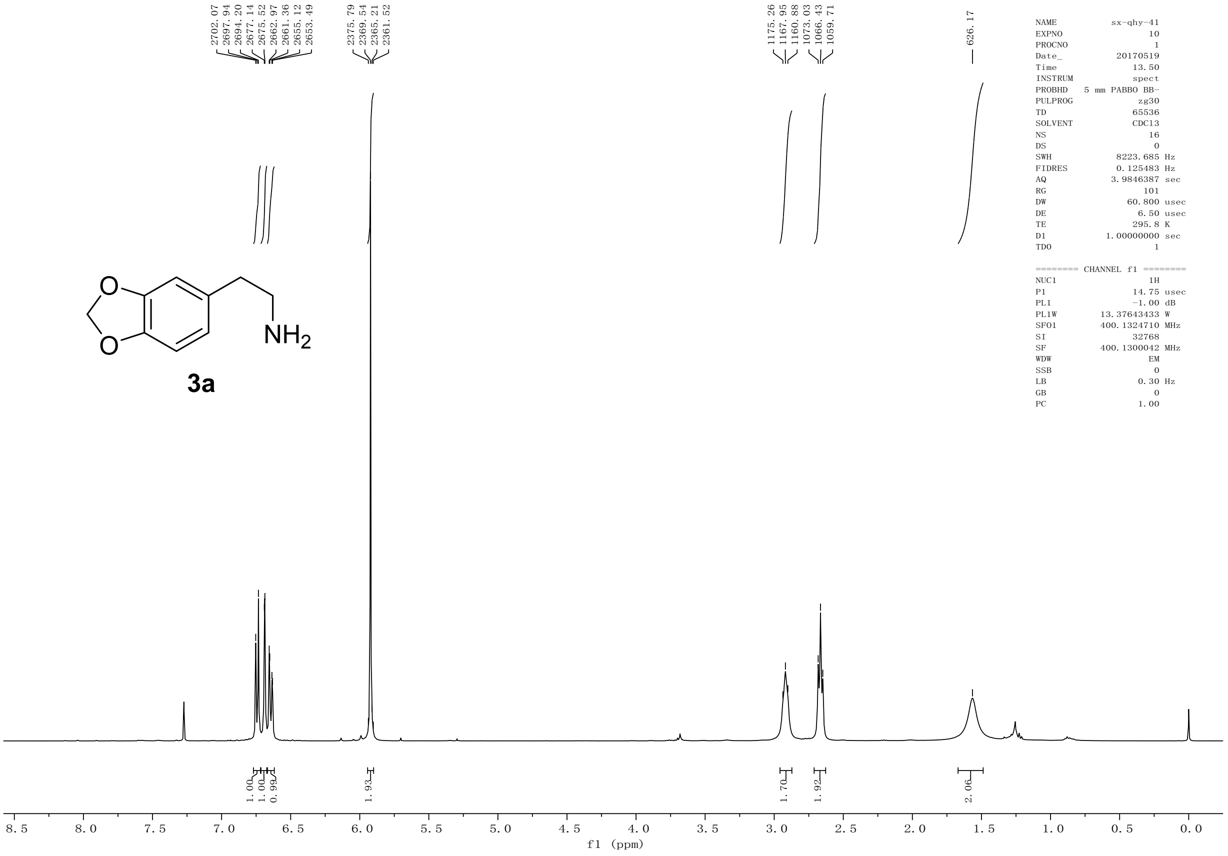

^{13}C NMR ($CDCl_3$-TFA, 100 MHz) spectrum of compound **3a**:

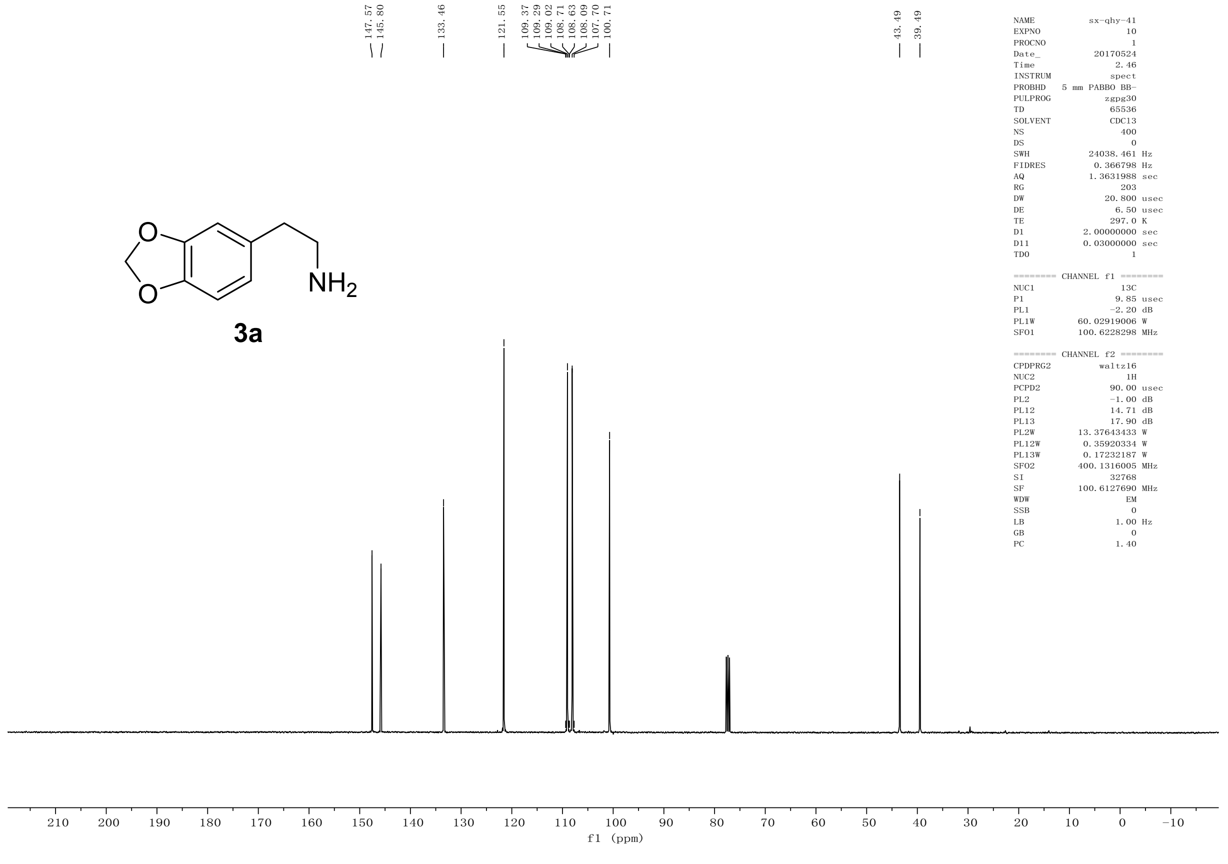

^{1}H NMR ($CDCl_3$, 400 MHz) spectrum of compound **3b**:

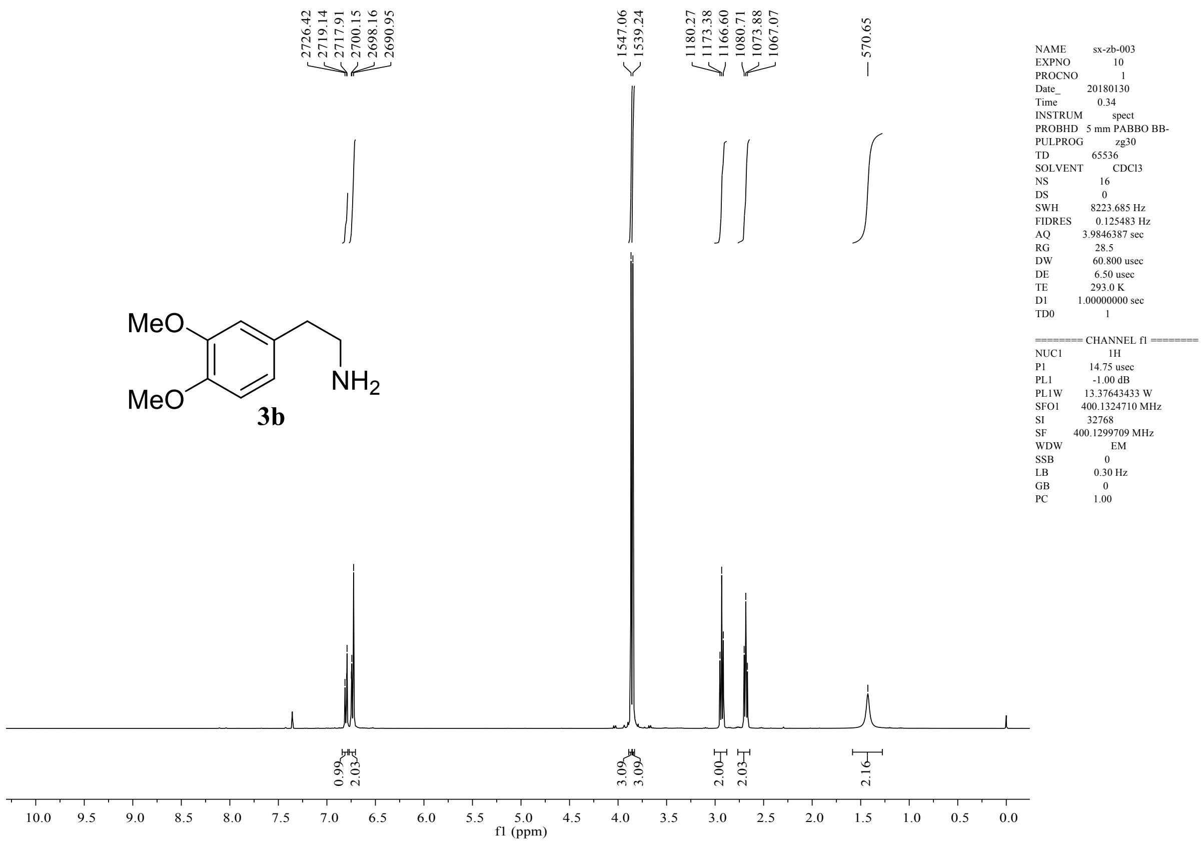

^{13}C NMR ($CDCl_3$, 100 MHz) spectrum of compound **3b**:

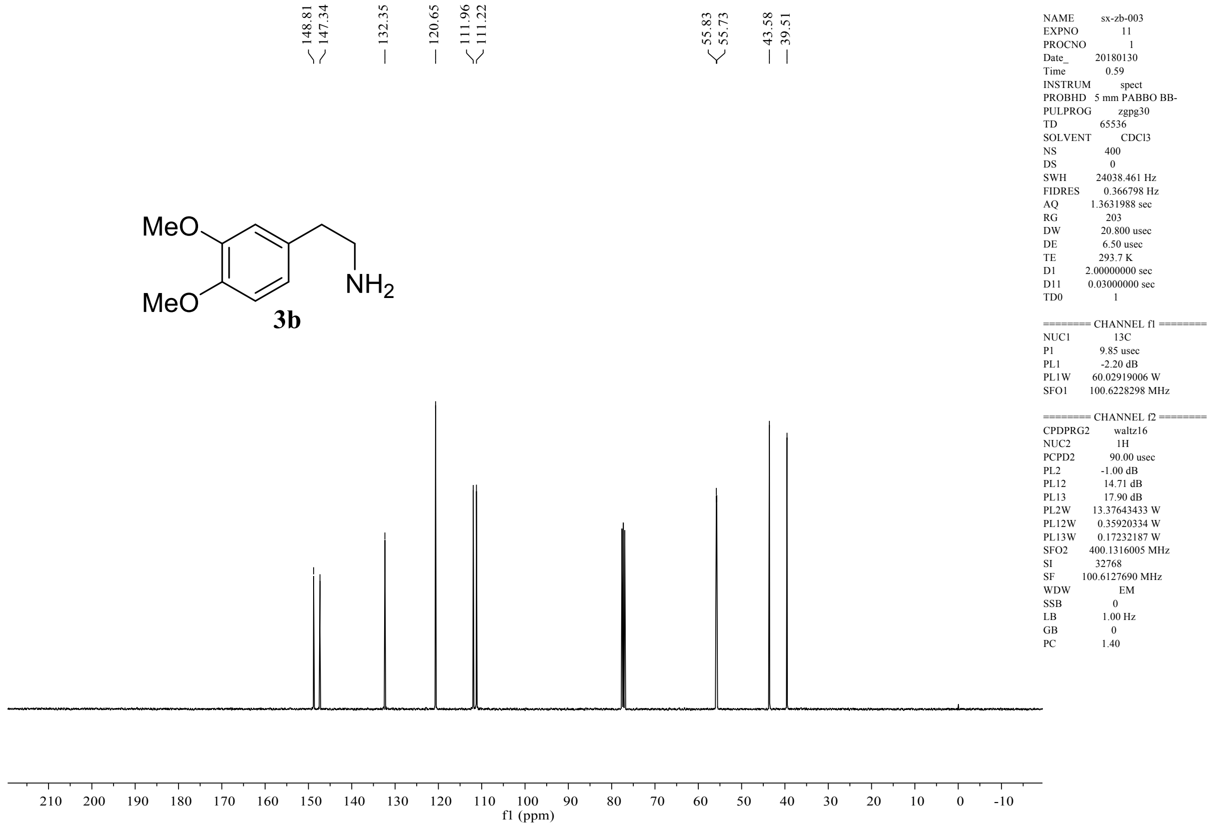

^{1}H NMR ($CDCl_3$, 400 MHz) spectrum of compound **3c**:

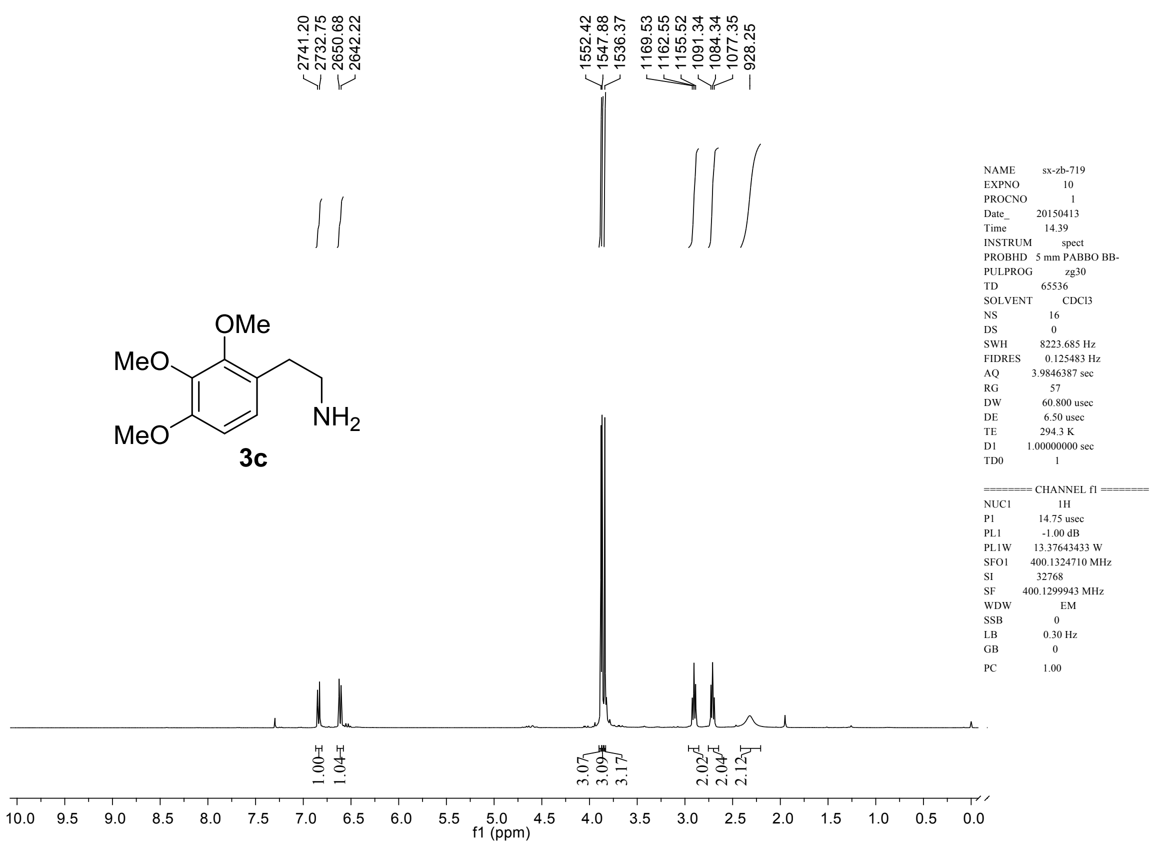

^{13}C NMR ($CDCl_3$, 100 MHz) spectrum of compound **3c**:

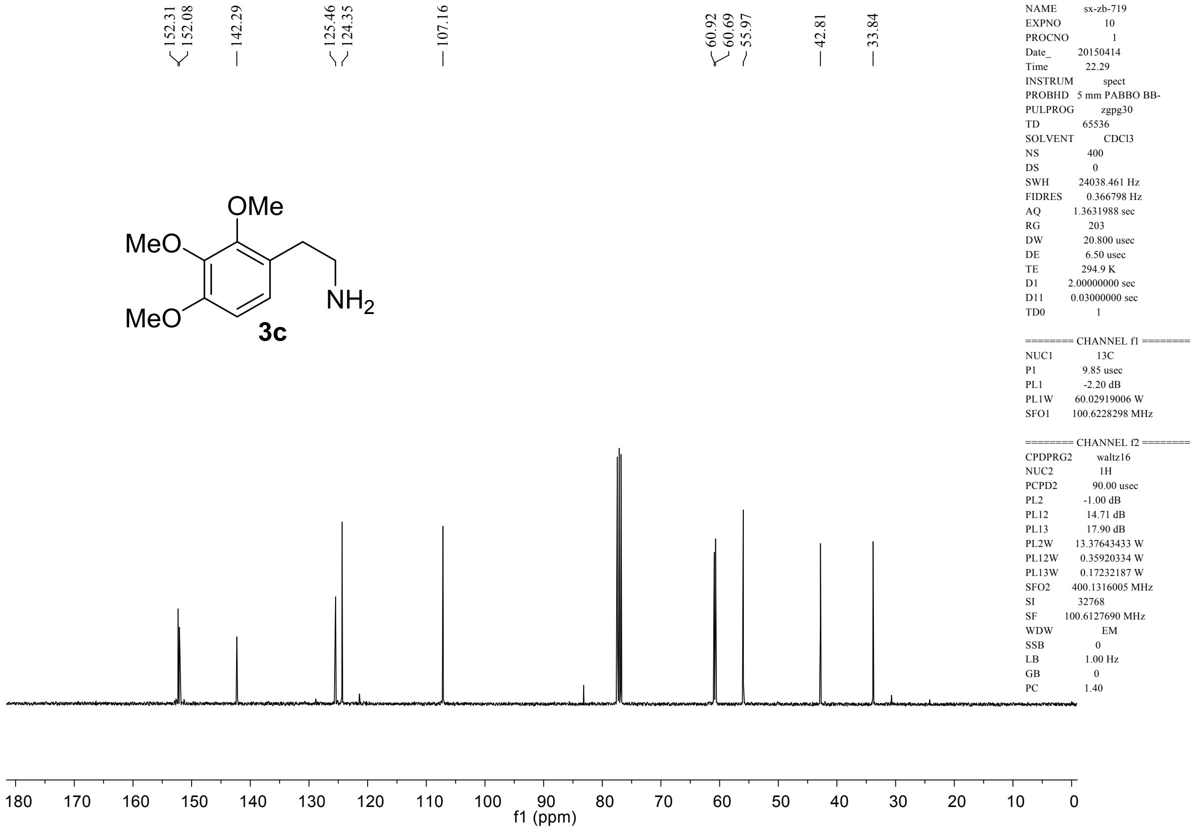

^{1}H NMR ($CDCl_3$, 400 MHz) spectrum of compound **4a**:

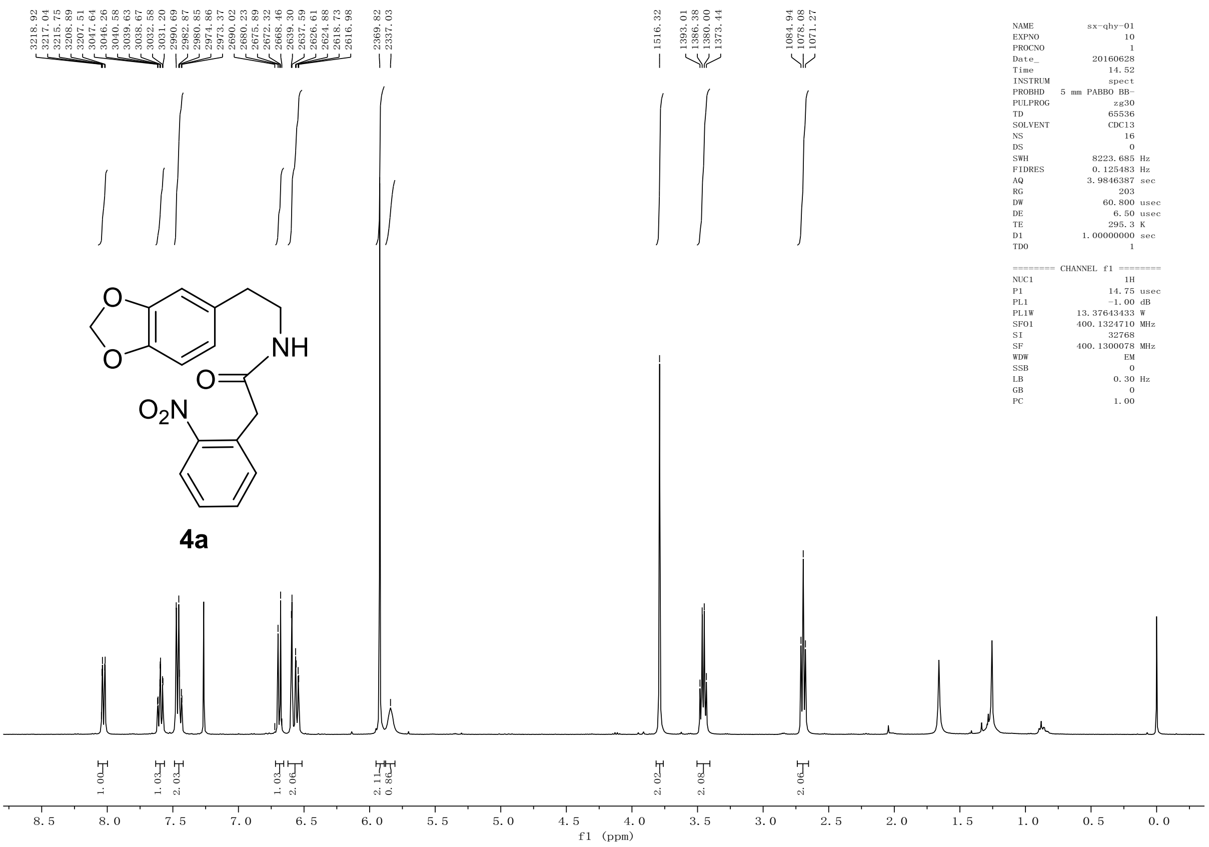

^{13}C NMR ($CDCl_3$, 100 MHz) spectrum of compound **4a**:

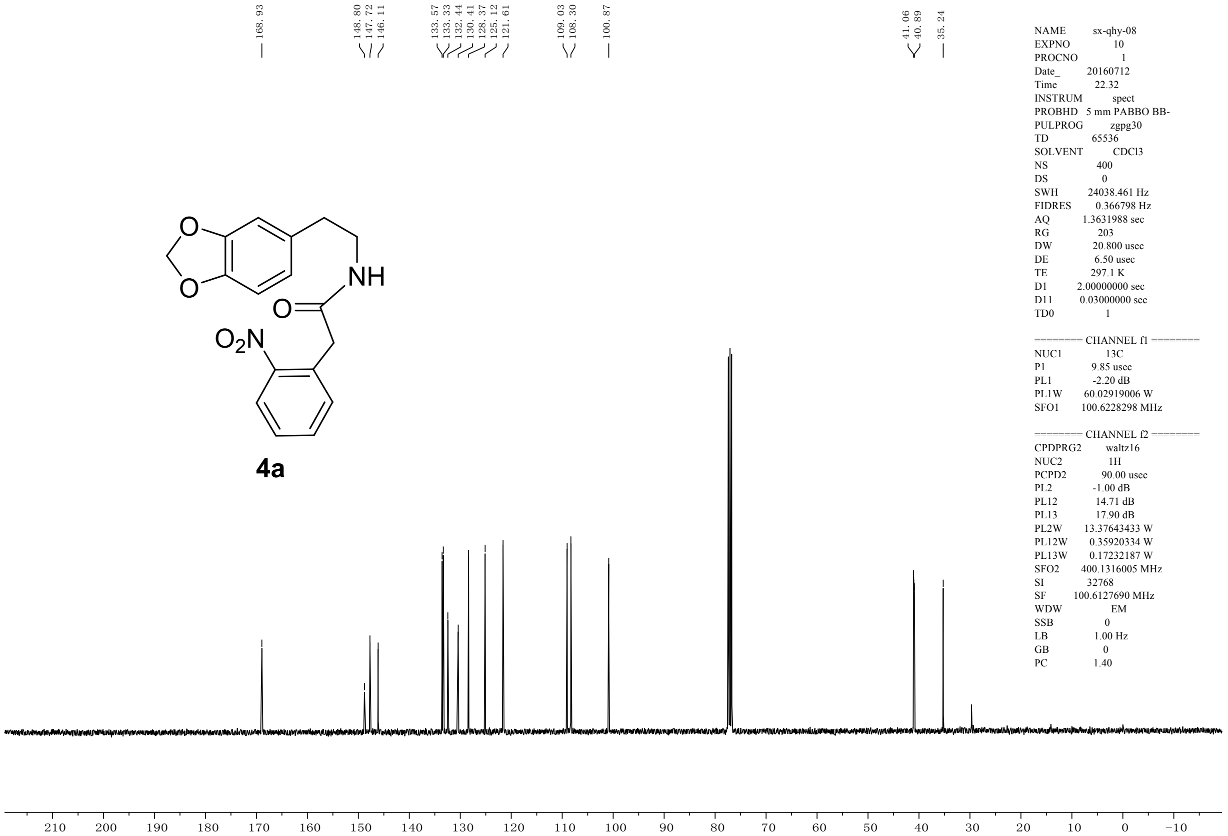

^{1}H NMR (DMSO-d_6, 400 MHz) spectrum of compound **4b**:

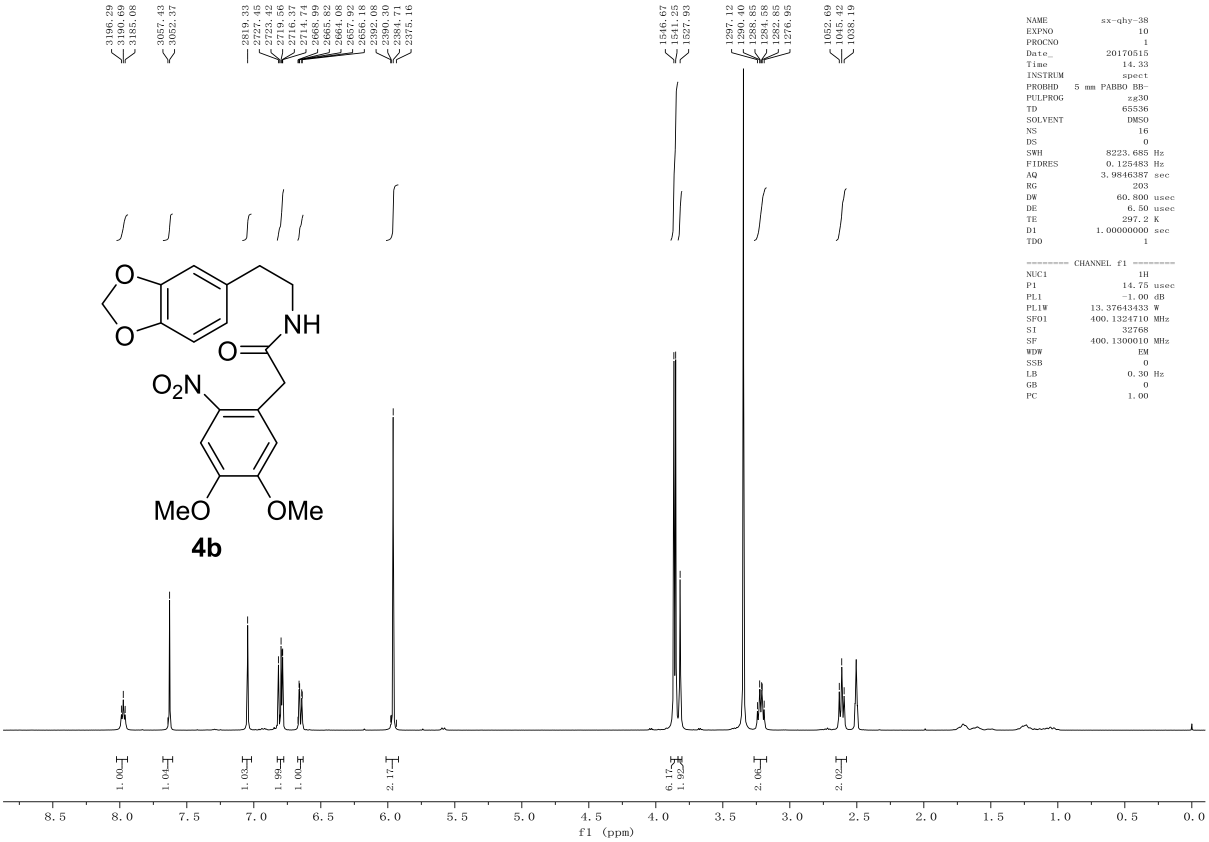

^{13}C NMR (DMSO-d_6, 100 MHz) spectrum of compound **4b**:

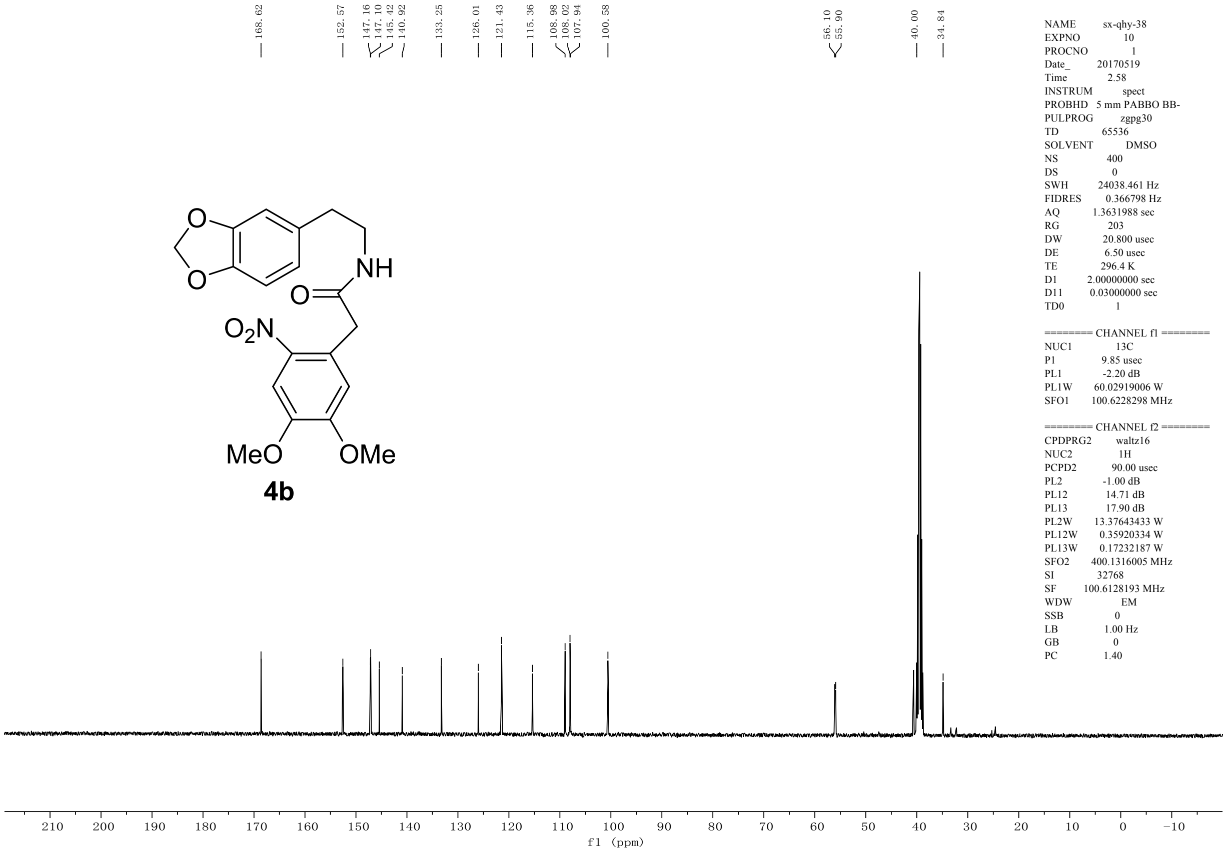

^{1}H NMR (DMSO-d_6, 400 MHz) spectrum of compound **4c**:

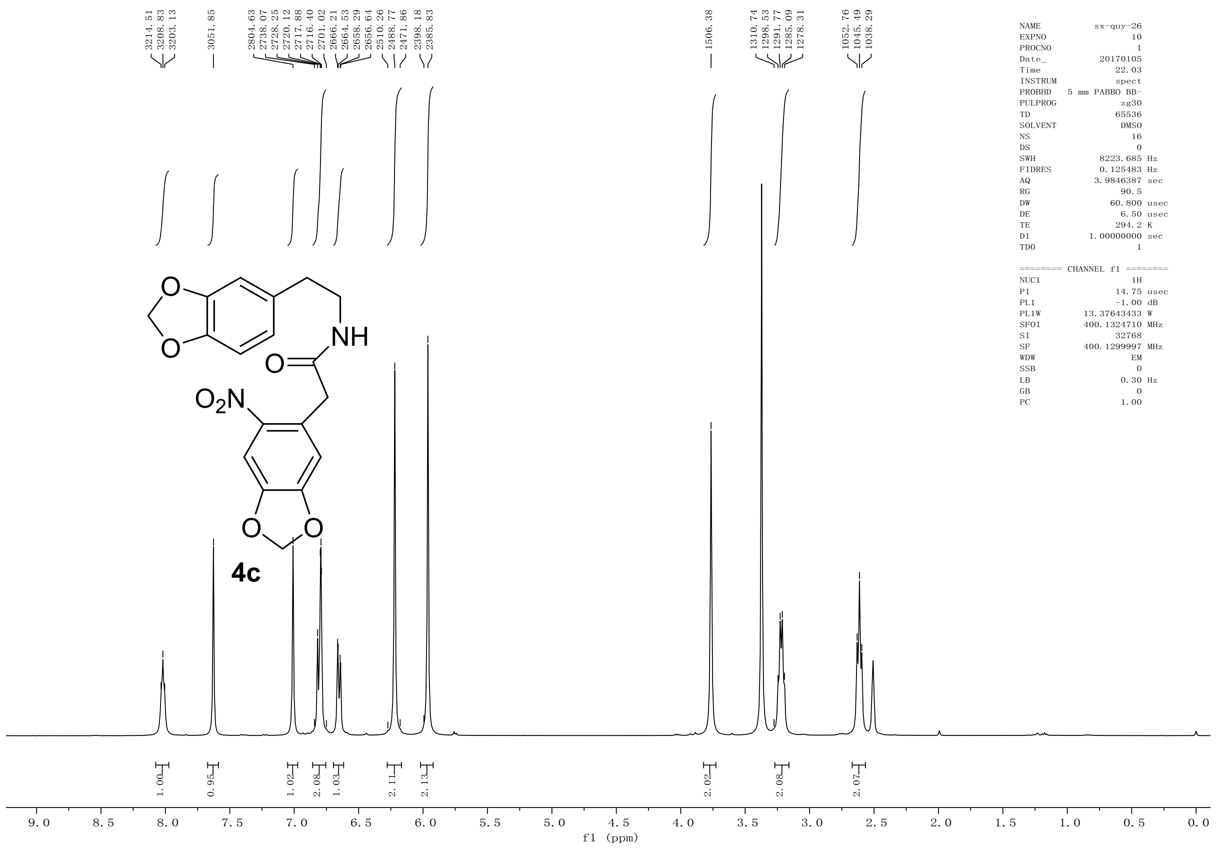

^{13}C NMR (DMSO-d_6, 100 MHz) spectrum of compound **4c**:

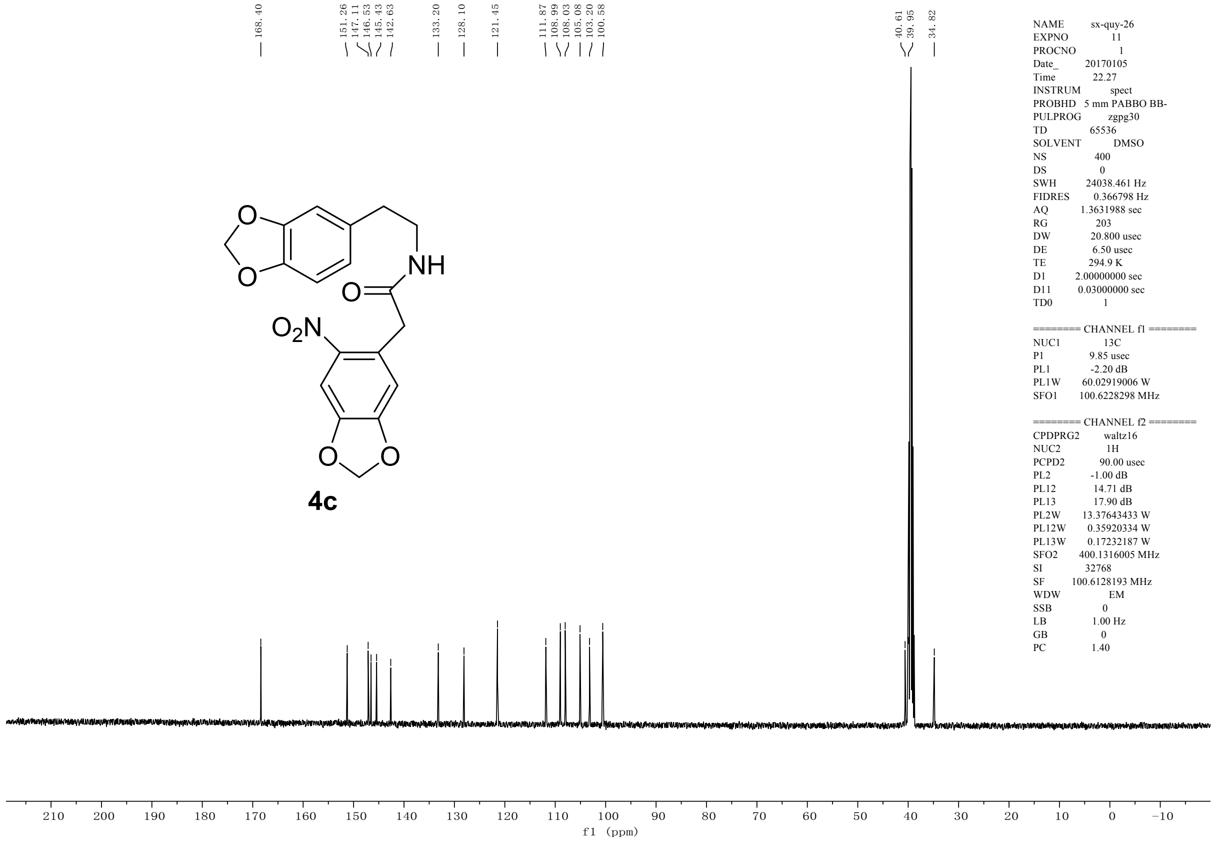

^{1}H NMR ($CDCl_3$, 400 MHz) spectrum of compound **4d**:

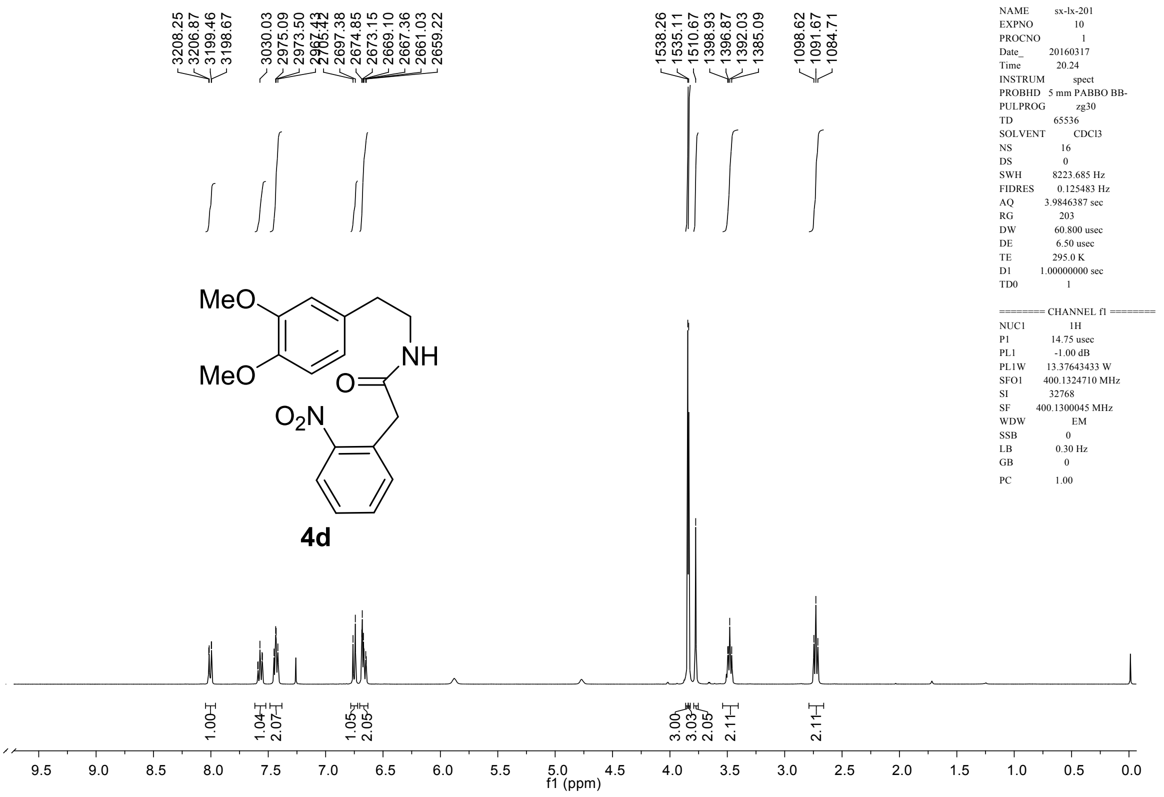

^{13}C NMR ($CDCl_3$, 100 MHz) spectrum of compound **4d**:

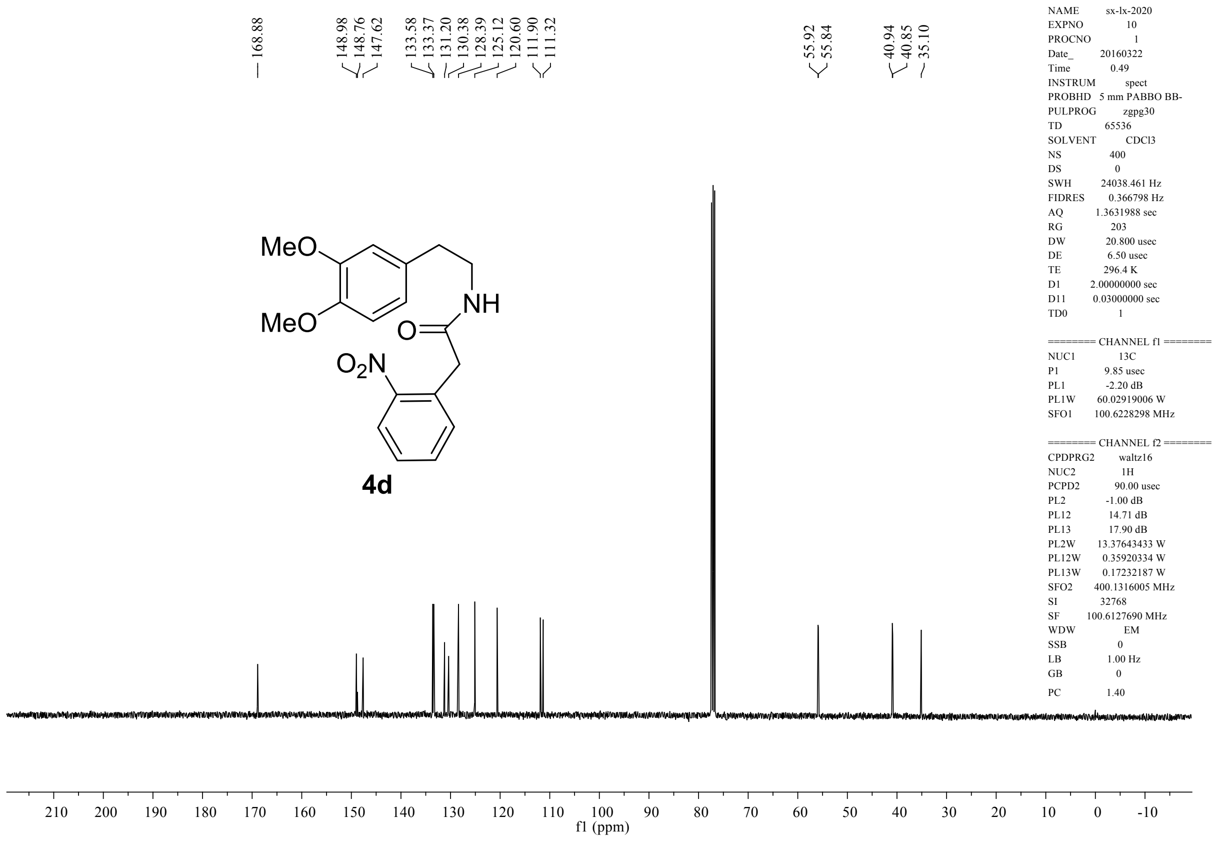

^{1}H NMR ($CDCl_3$, 400 MHz) spectrum of compound **4e**:

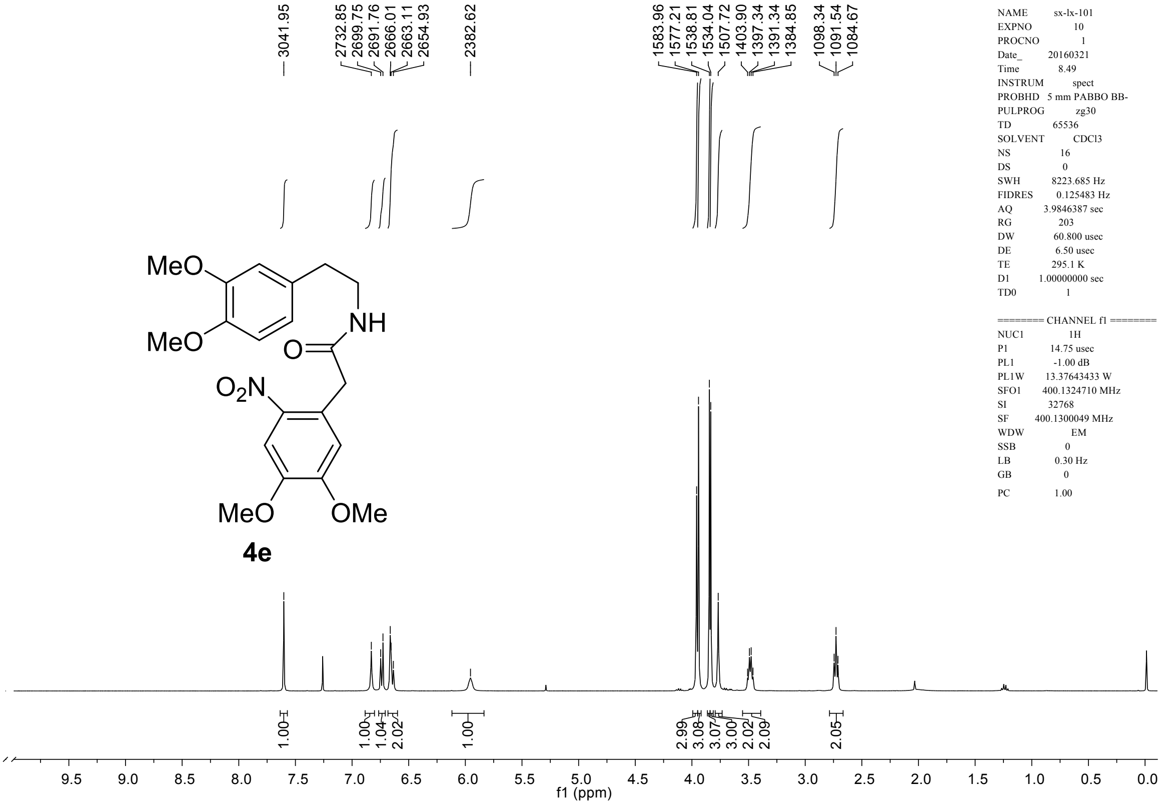

^{13}C NMR ($CDCl_3$, 100 MHz) spectrum of compound **4e**:

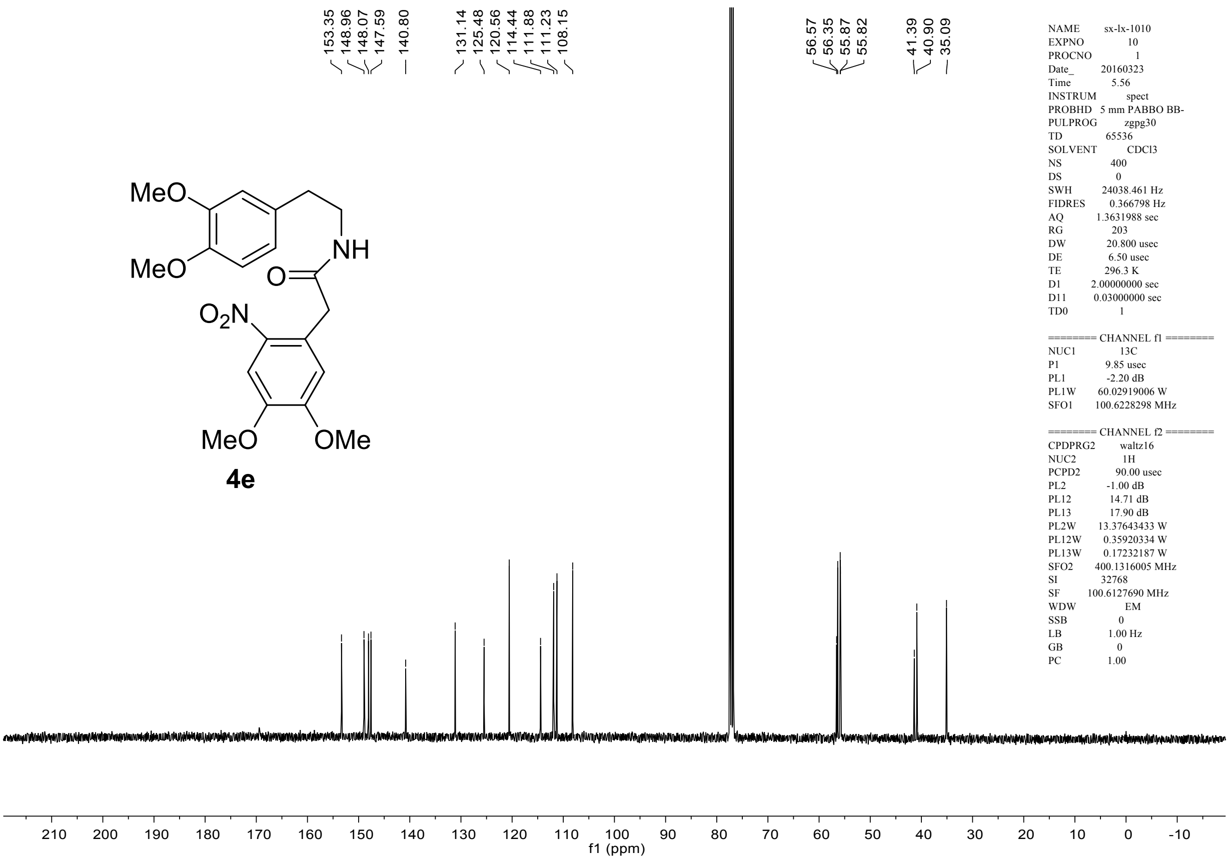

^{1}H NMR ($CDCl_3$, 400 MHz) spectrum of compound **4f**:

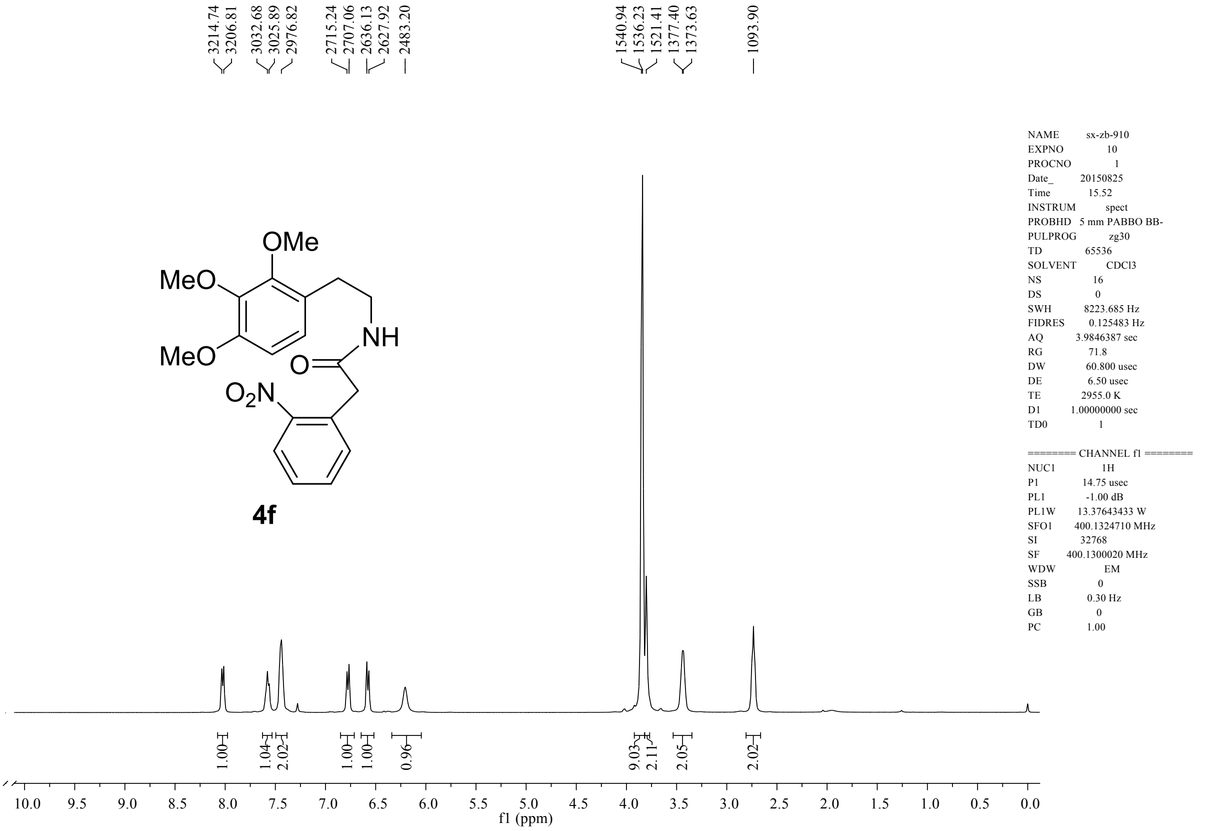

^{13}C NMR ($CDCl_3$, 100 MHz) spectrum of compound **4f**:

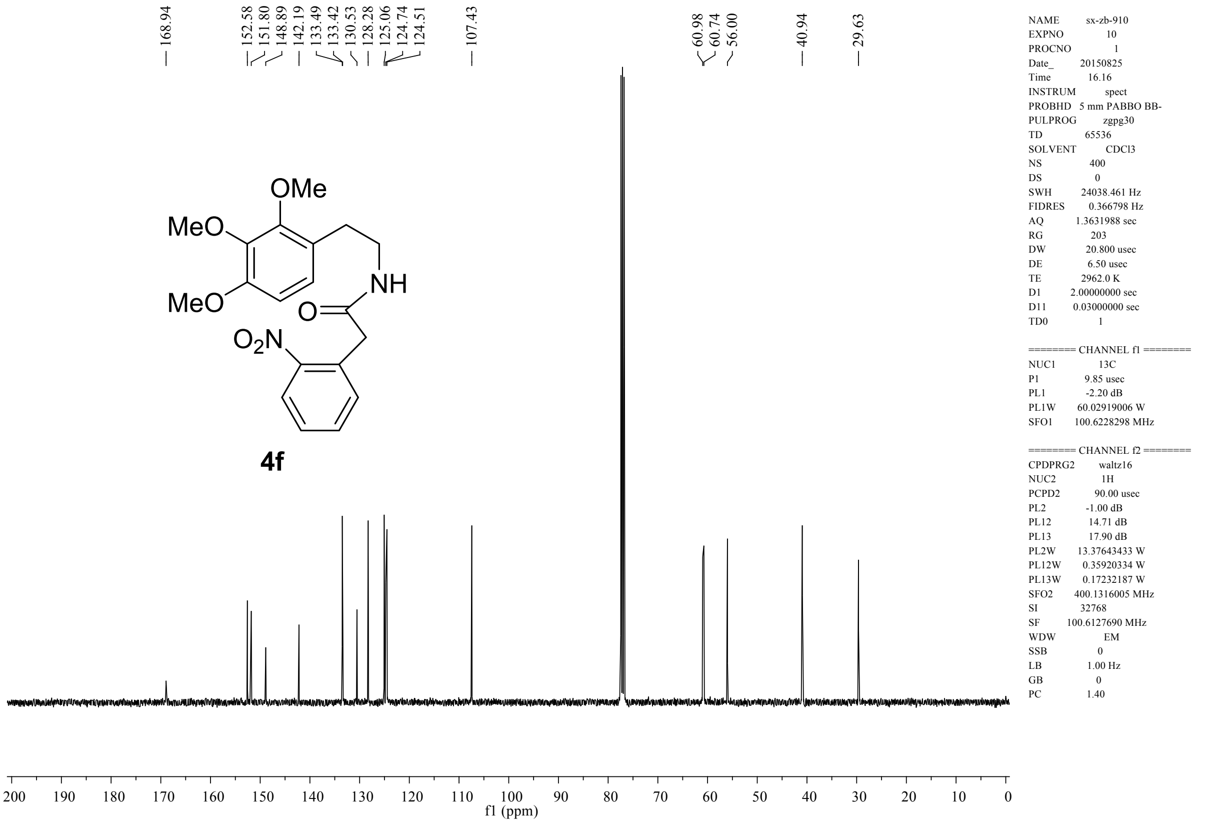

^{1}H NMR ($CDCl_3$-TFA, 400 MHz) spectrum of compound **6a**:

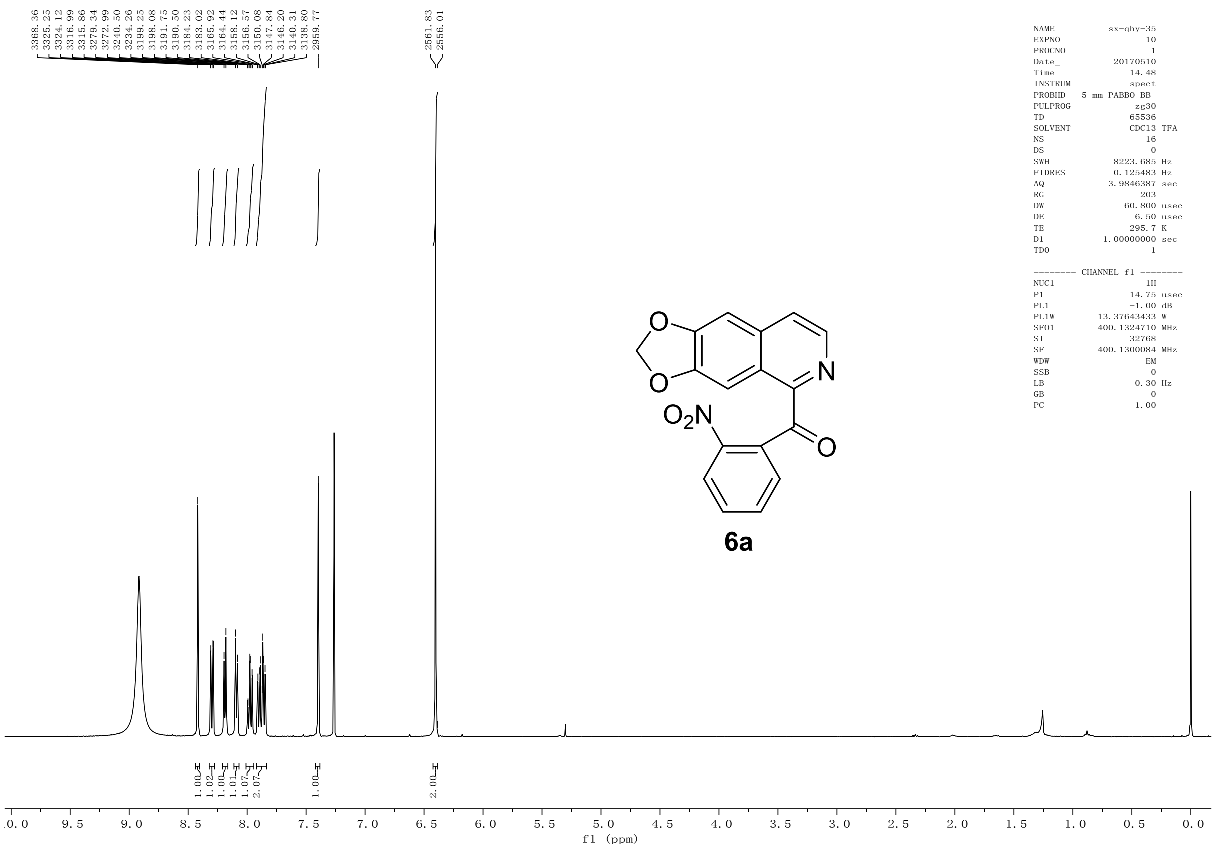

^{13}C NMR ($CDCl_3$-TFA, 100 MHz) spectrum of compound **6a**:

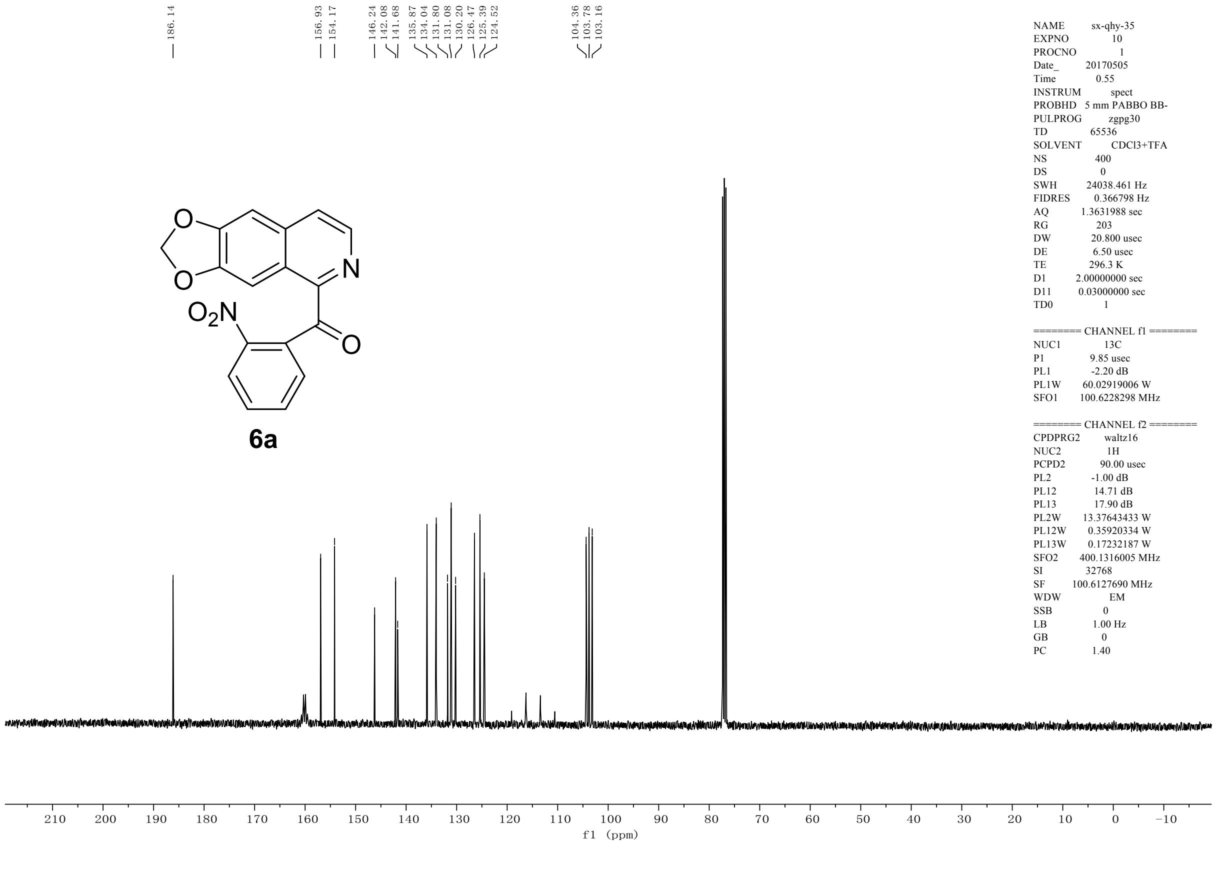

^{1}H NMR ($CDCl_3$-TFA, 400 MHz) spectrum of compound **6b**:

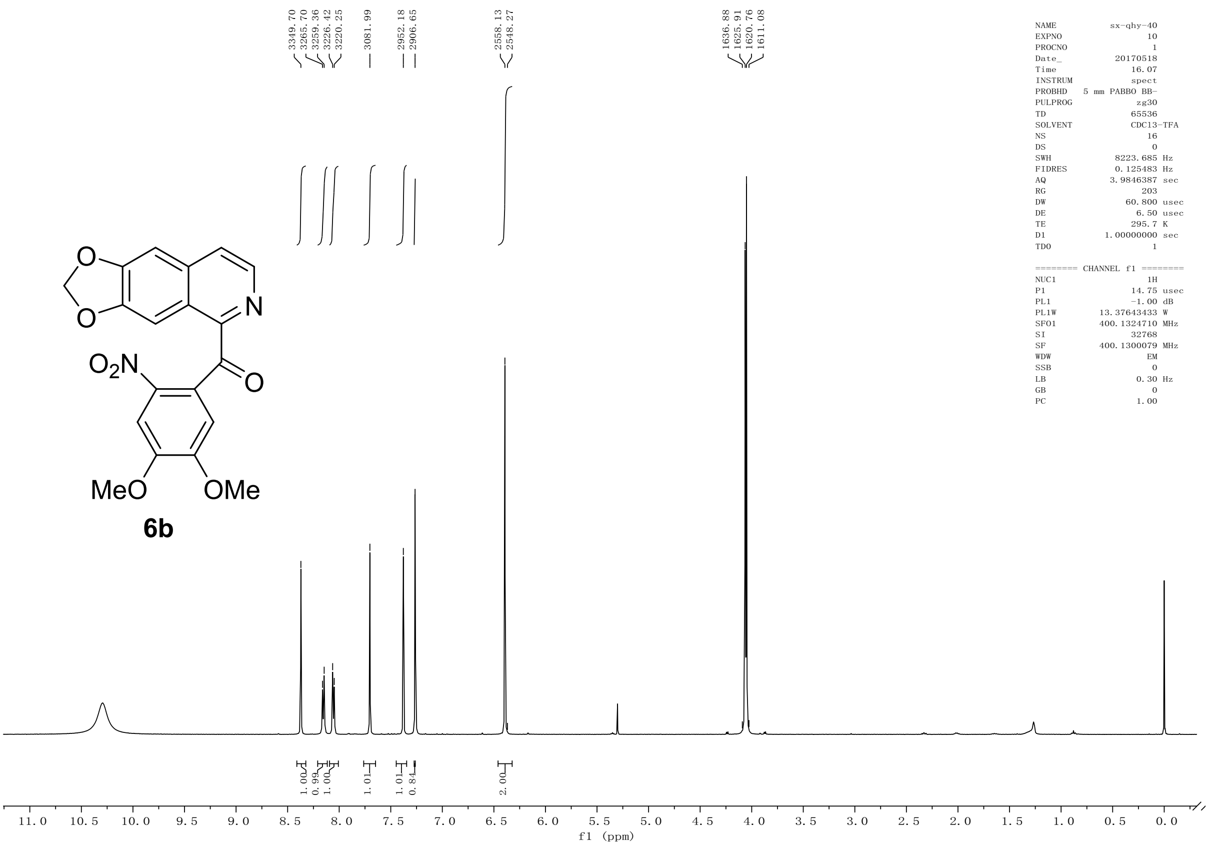

^{13}C NMR ($CDCl_3$-TFA, 100 MHz) spectrum of compound **6b**:

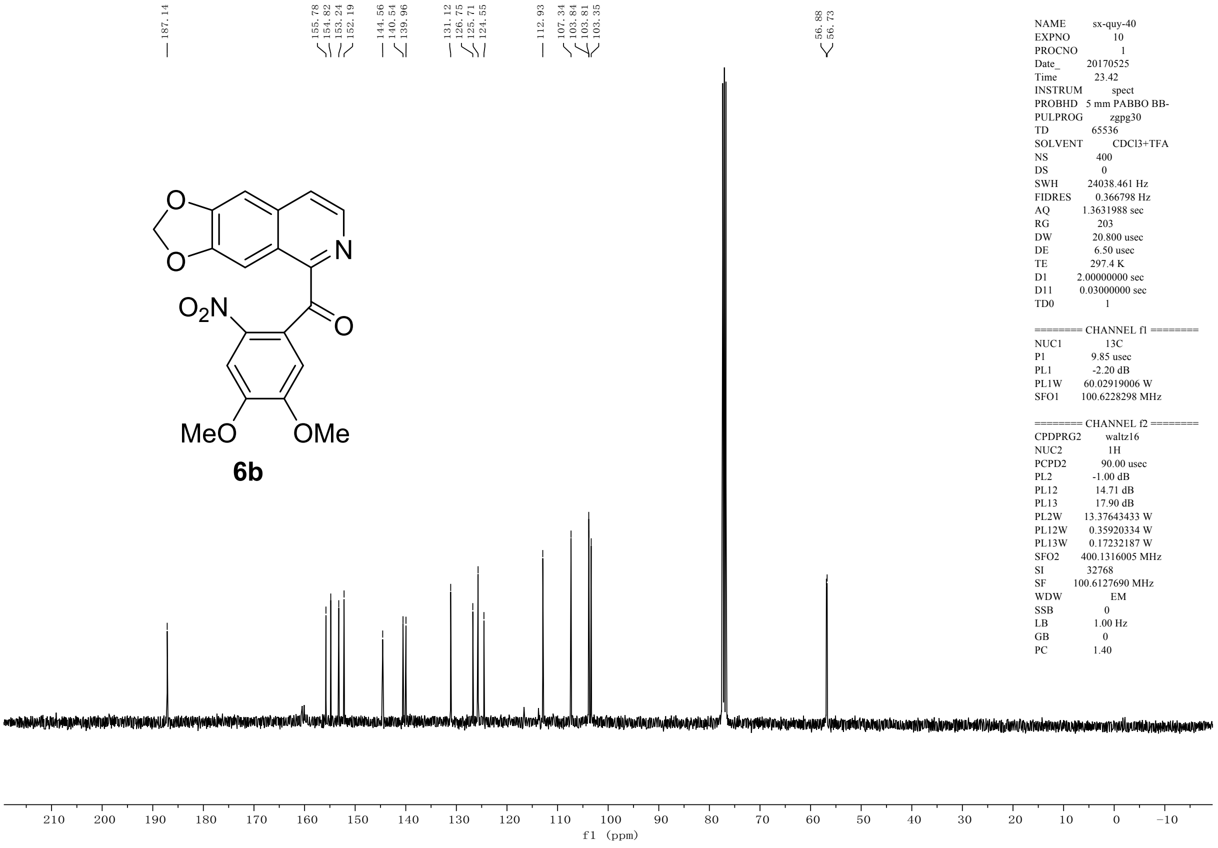

^{1}H NMR ($CDCl_3$-TFA, 400 MHz) spectrum of compound **6c**:

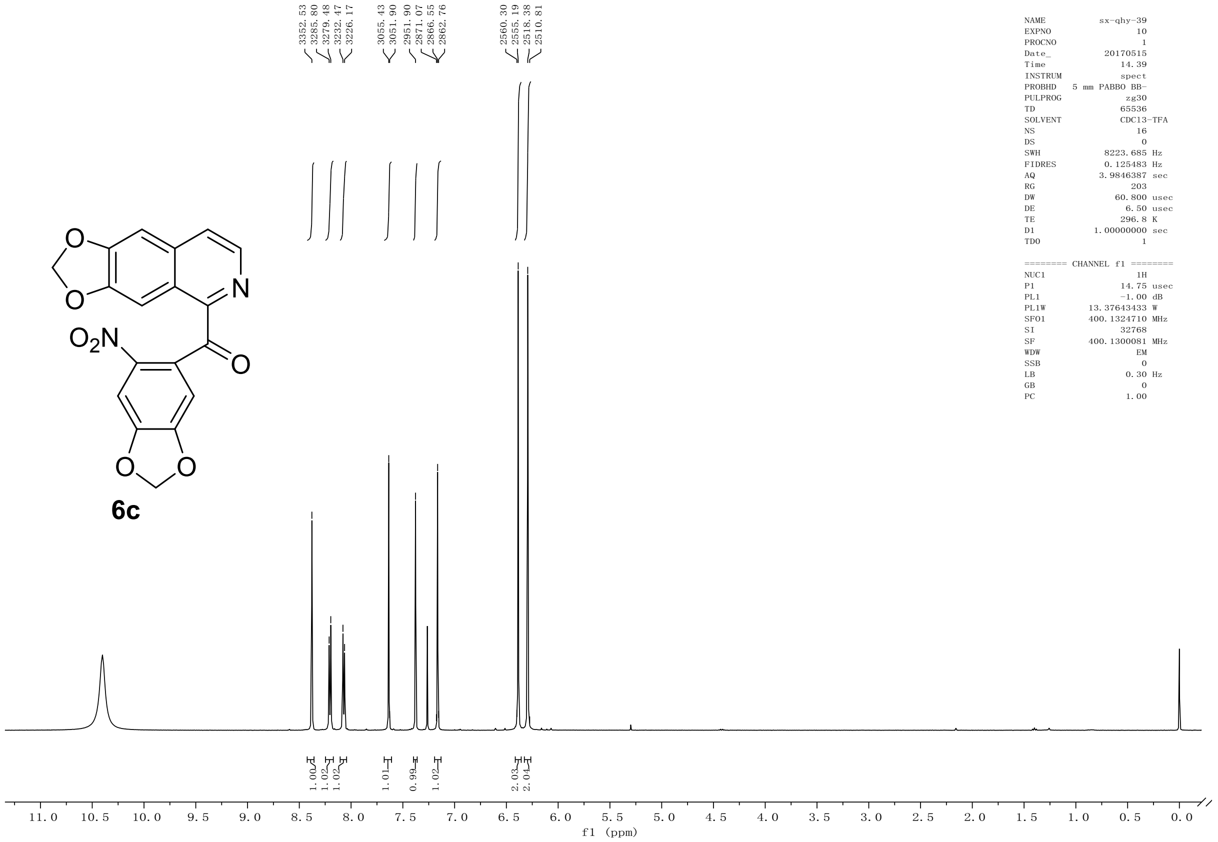

^{13}C NMR ($CDCl_3$-TFA, 100 MHz) spectrum of compound **6c**:

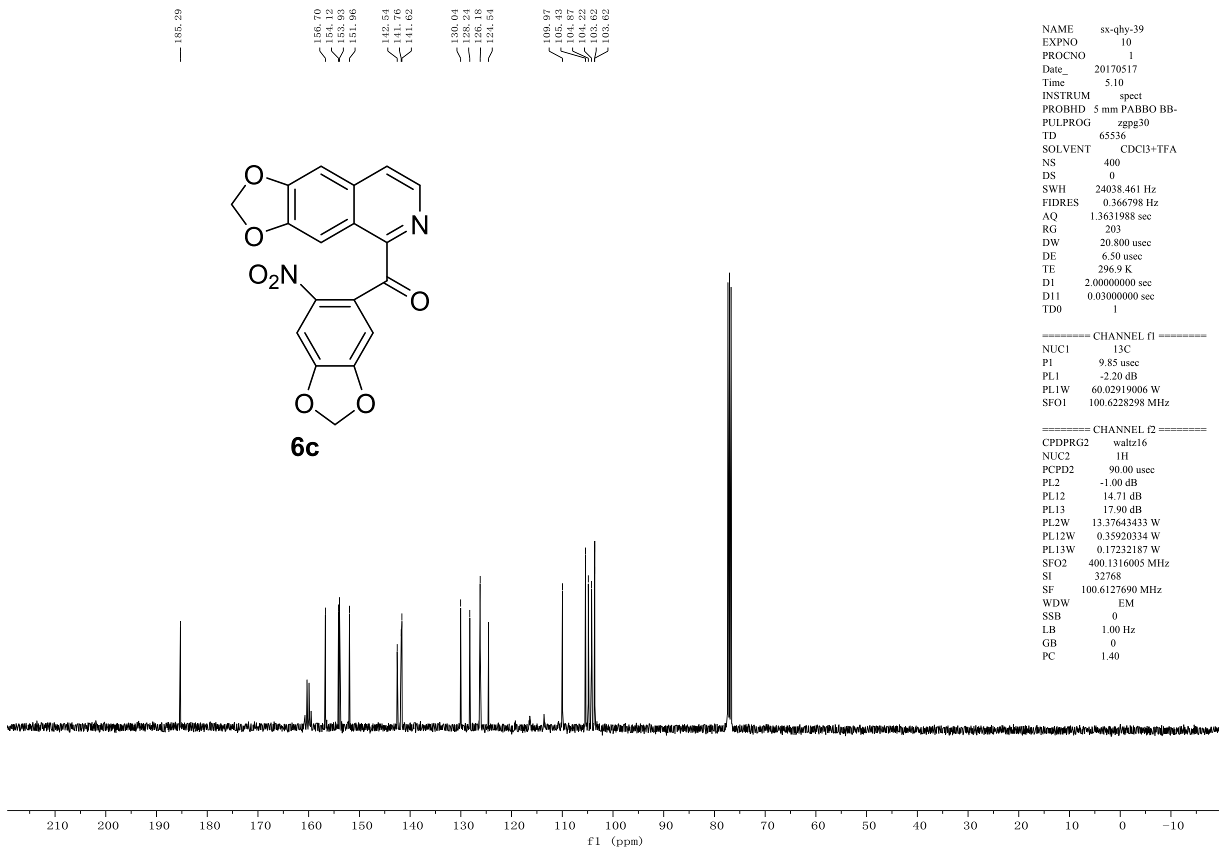

^{1}H NMR ($CDCl_3$, 400 MHz) spectrum of compound **6d**:

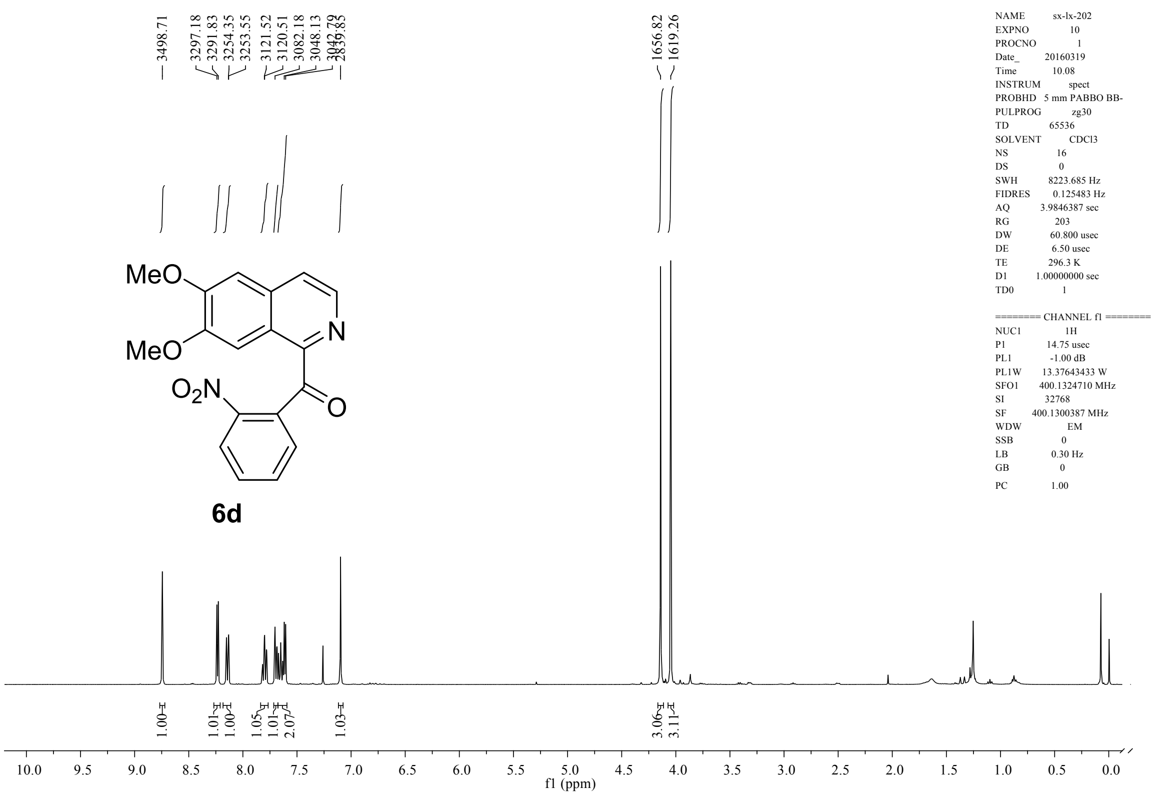

^{13}C NMR ($CDCl_3$, 100 MHz) spectrum of compound **6d**:

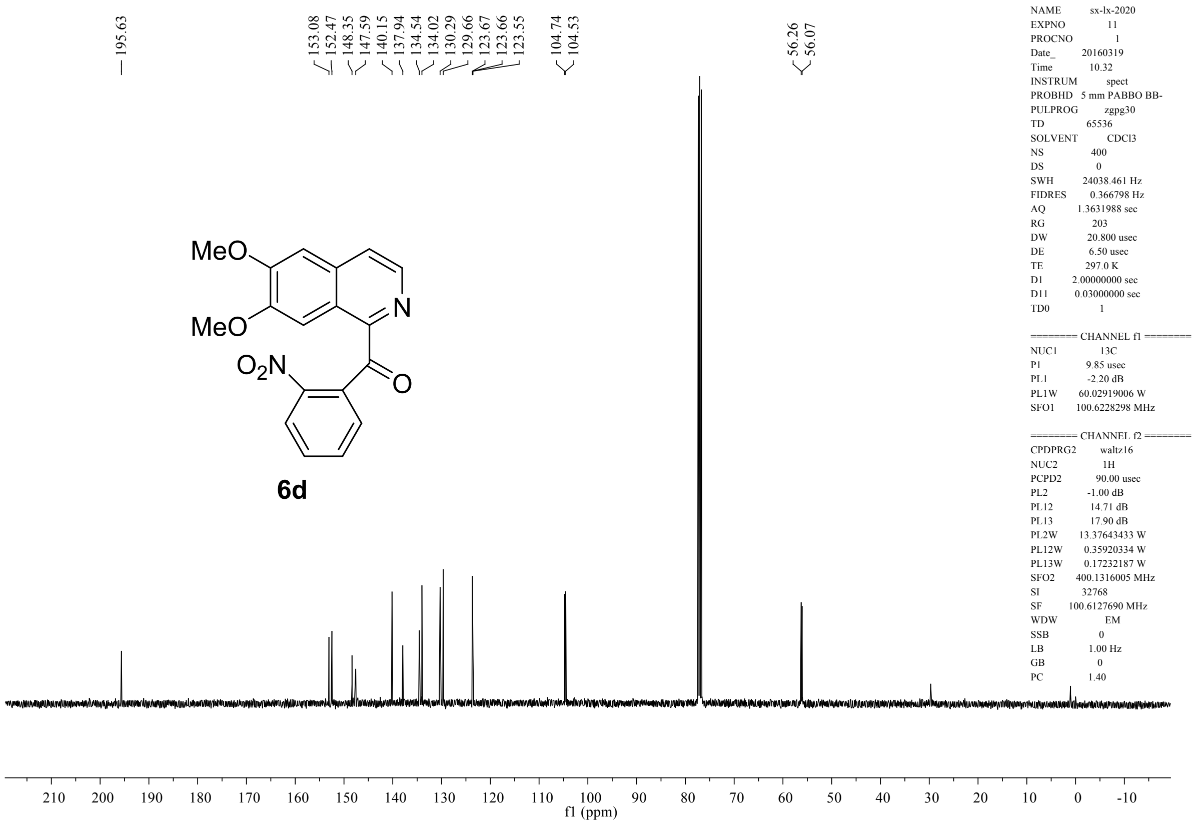

^{1}H NMR ($CDCl_3$, 400 MHz) spectrum of compound **6e**:

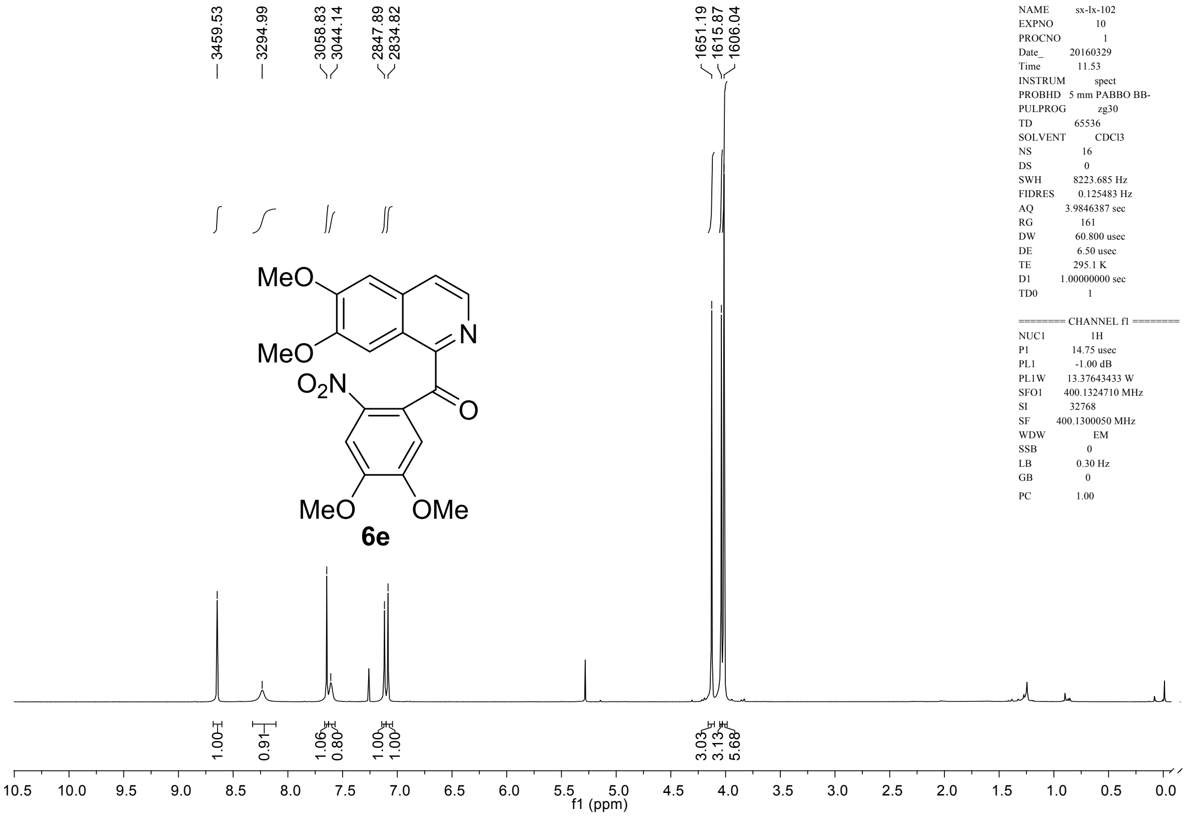

^{13}C NMR ($CDCl_3$, 100 MHz) spectrum of compound **6e**:

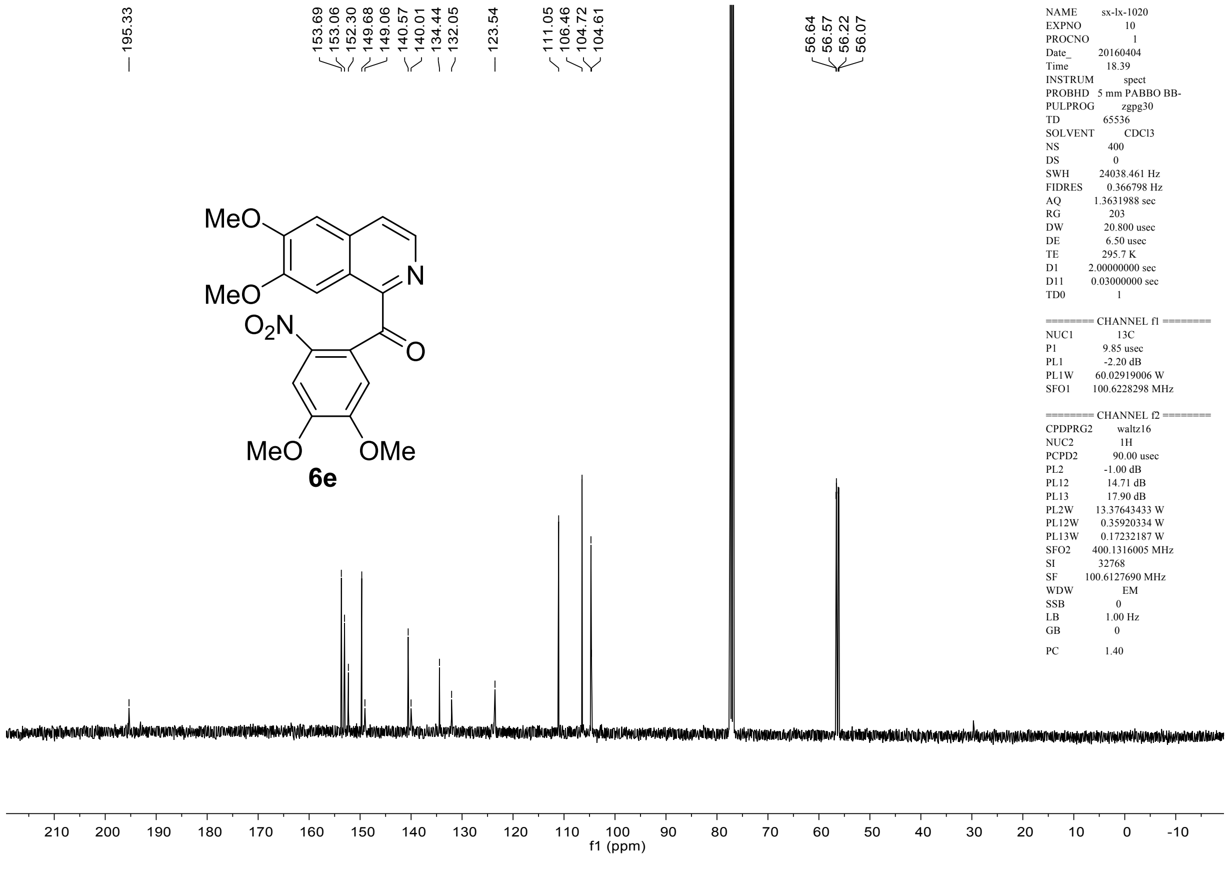

^{1}H NMR ($CDCl_3$, 400 MHz) spectrum of compound **6f**:

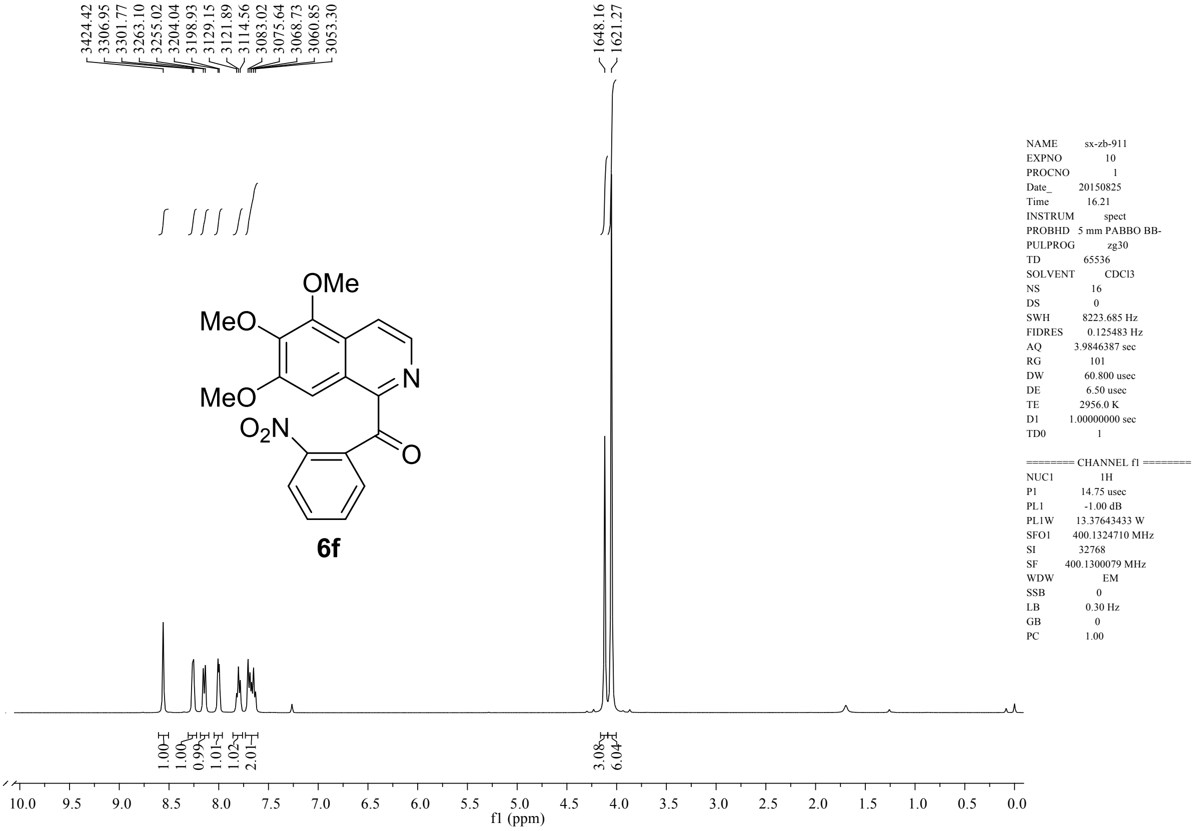

^{13}C NMR ($CDCl_3$, 100 MHz) spectrum of compound **6f**:

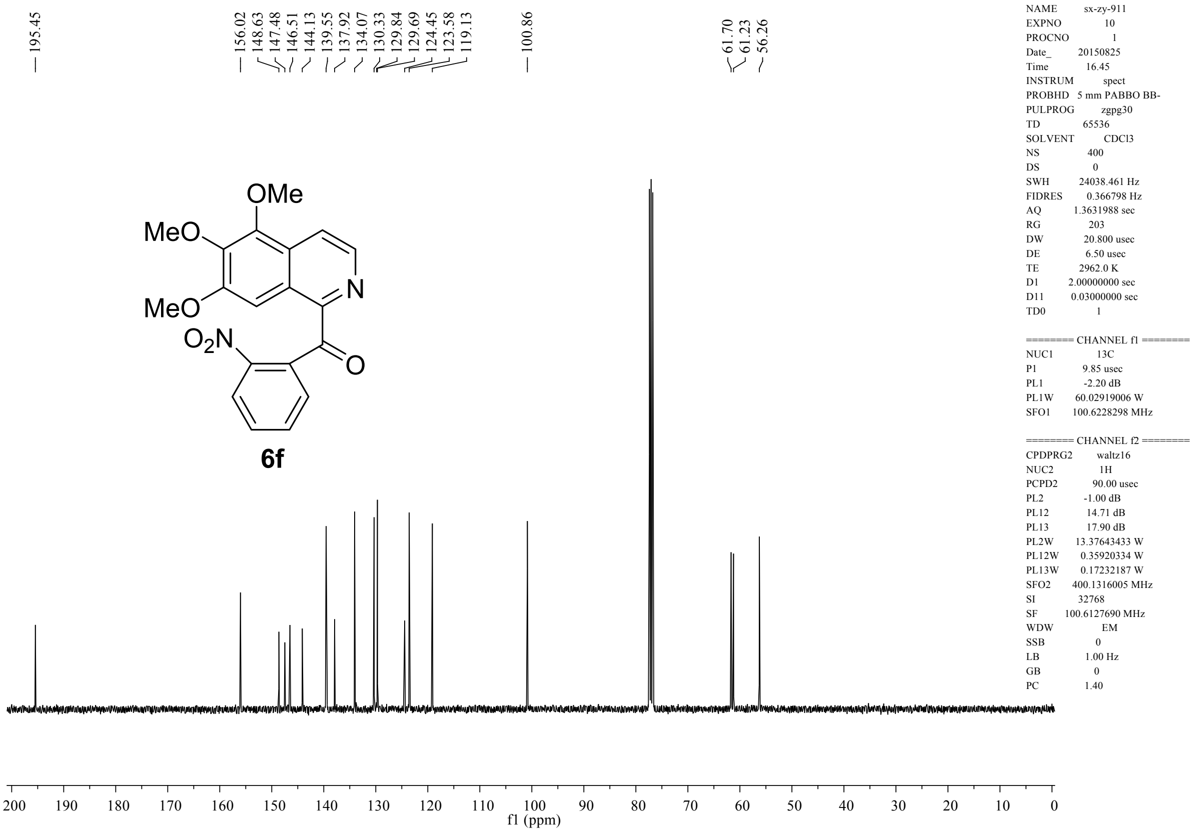

^{1}H NMR ($CDCl_3$, 400 MHz) spectrum of compound **6g**:

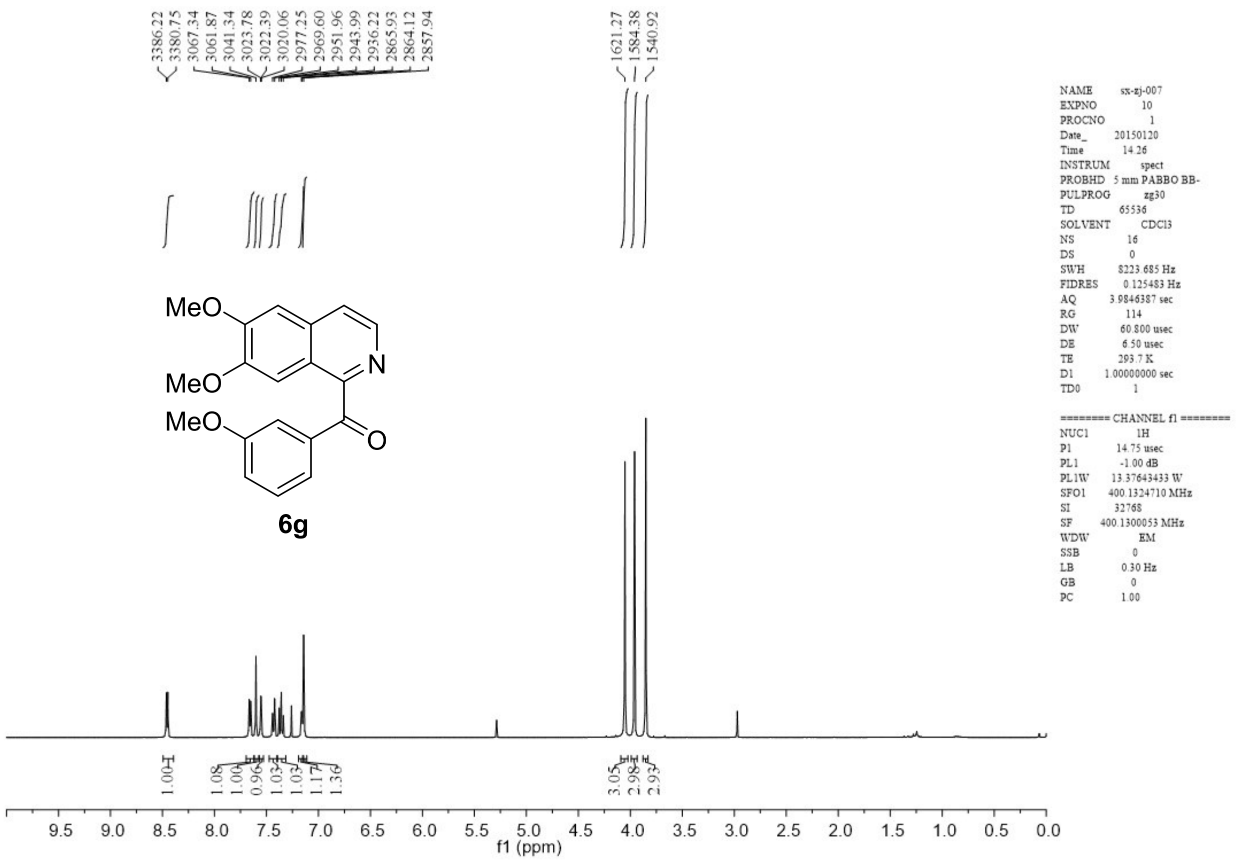

^{13}C NMR ($CDCl_3$, 100 MHz) spectrum of compound **6g**:

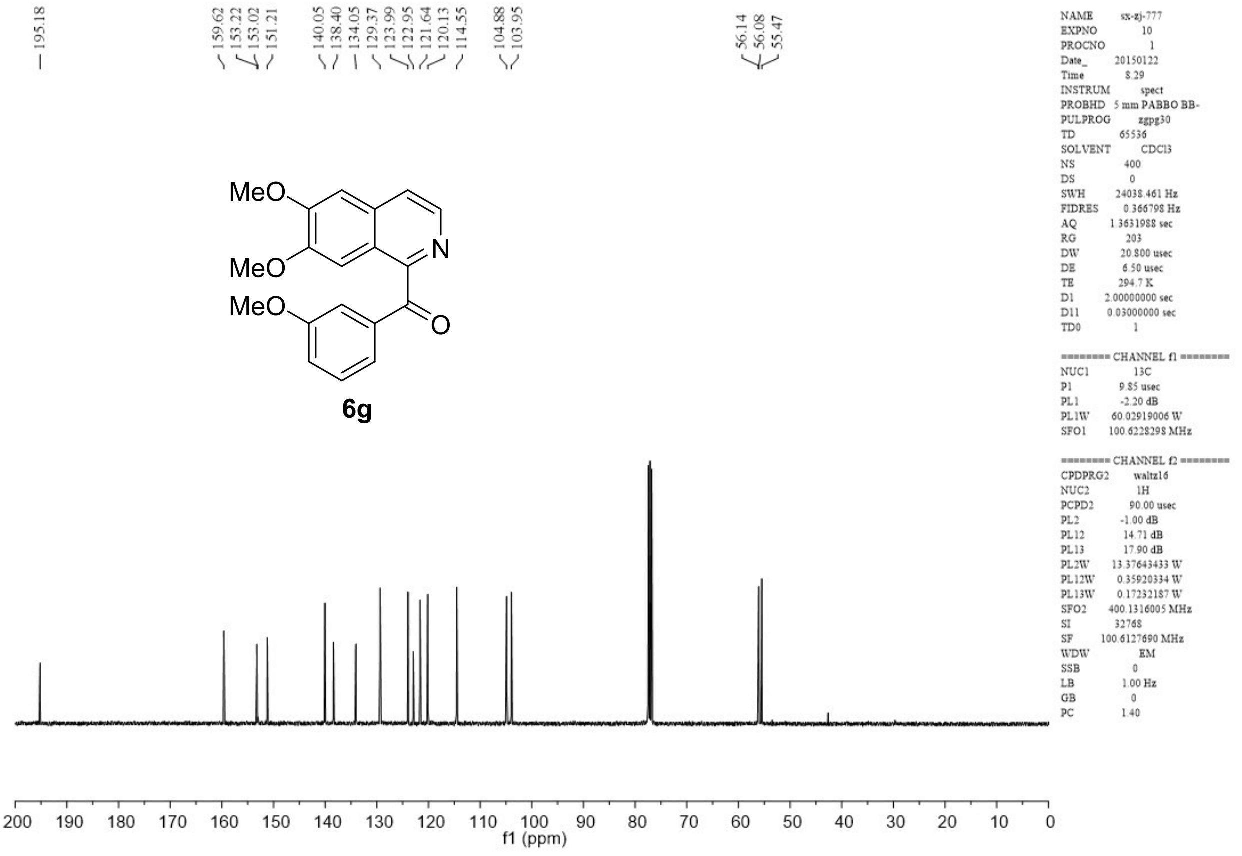

^{1}H NMR ($CDCl_3$, 400 MHz) spectrum of compound **6h**:

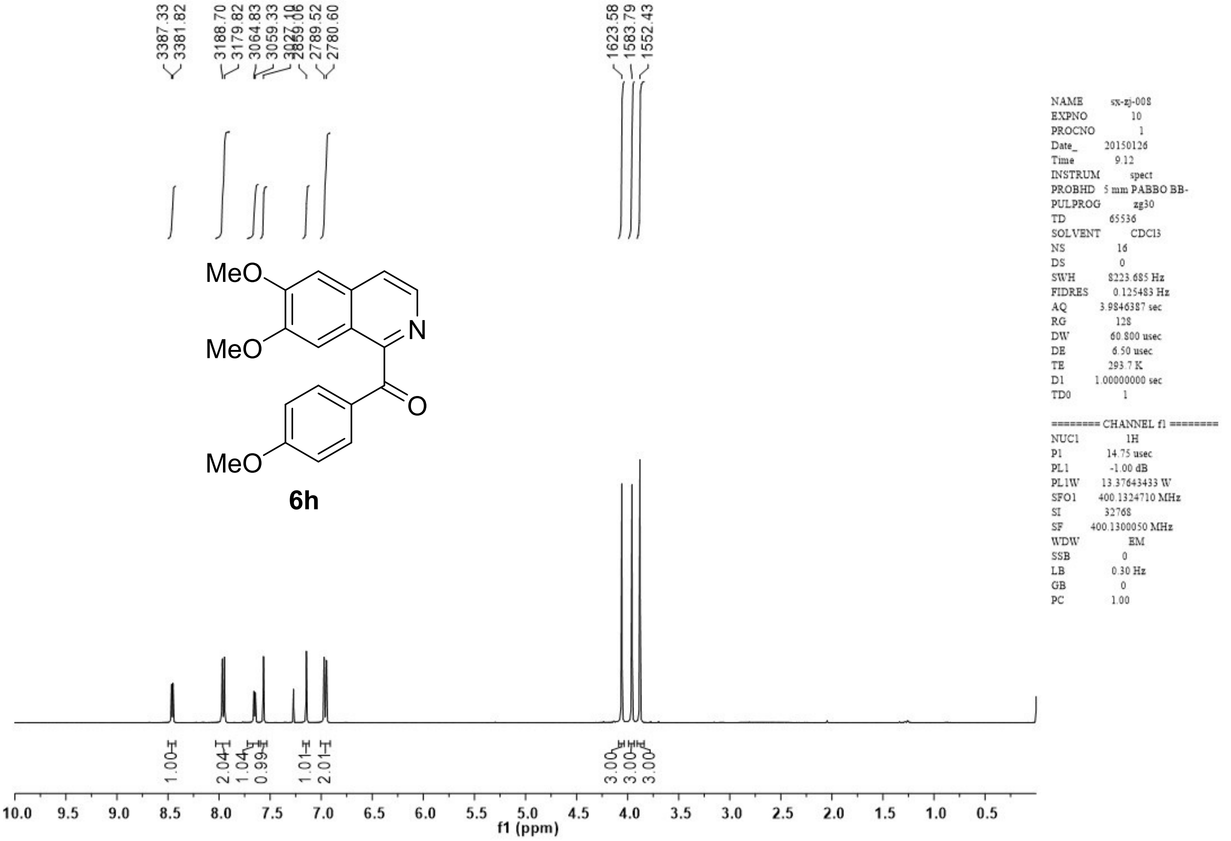

^{13}C NMR ($CDCl_3$, 100 MHz) spectrum of compound **6h**:

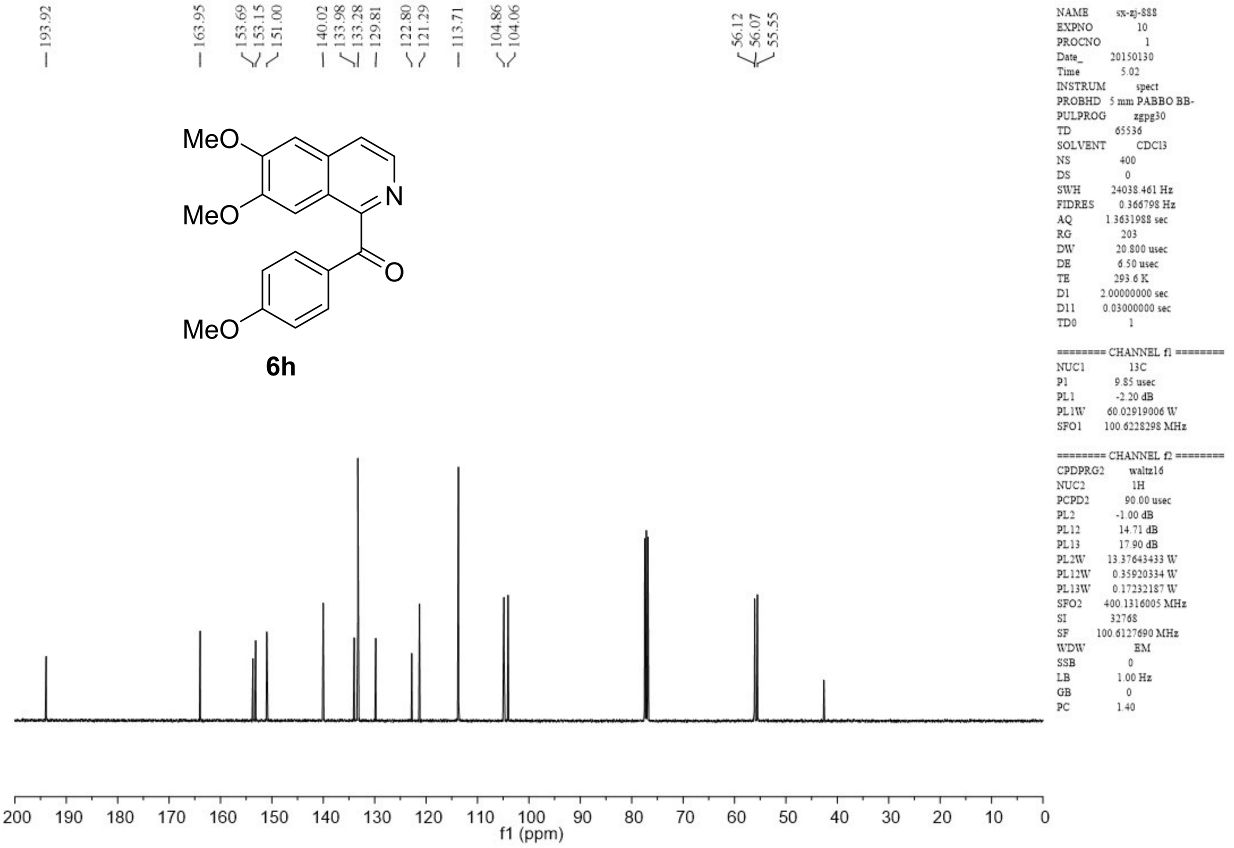

^{1}H NMR ($CDCl_3$, 400 MHz) spectrum of compound **6i**:

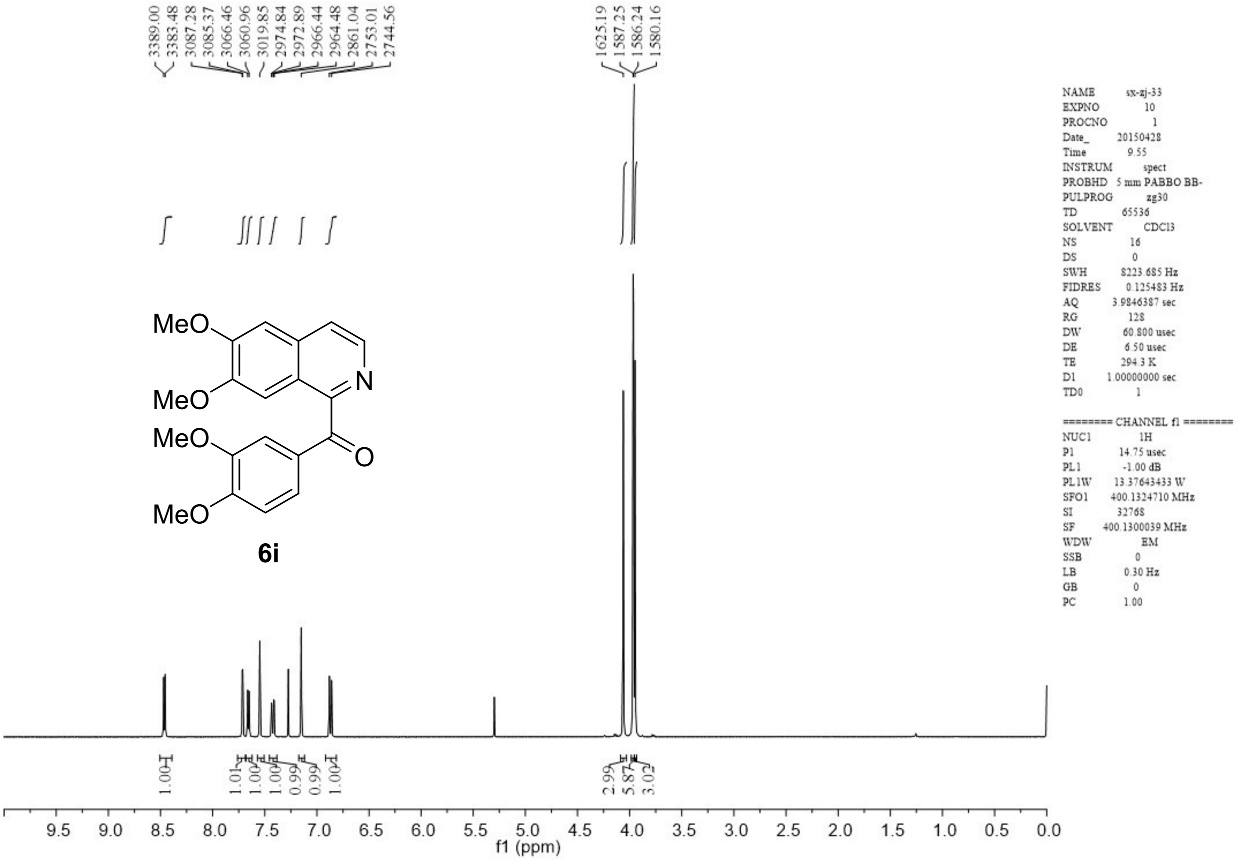

^{13}C NMR ($CDCl_3$, 100 MHz) spectrum of compound **6i**:

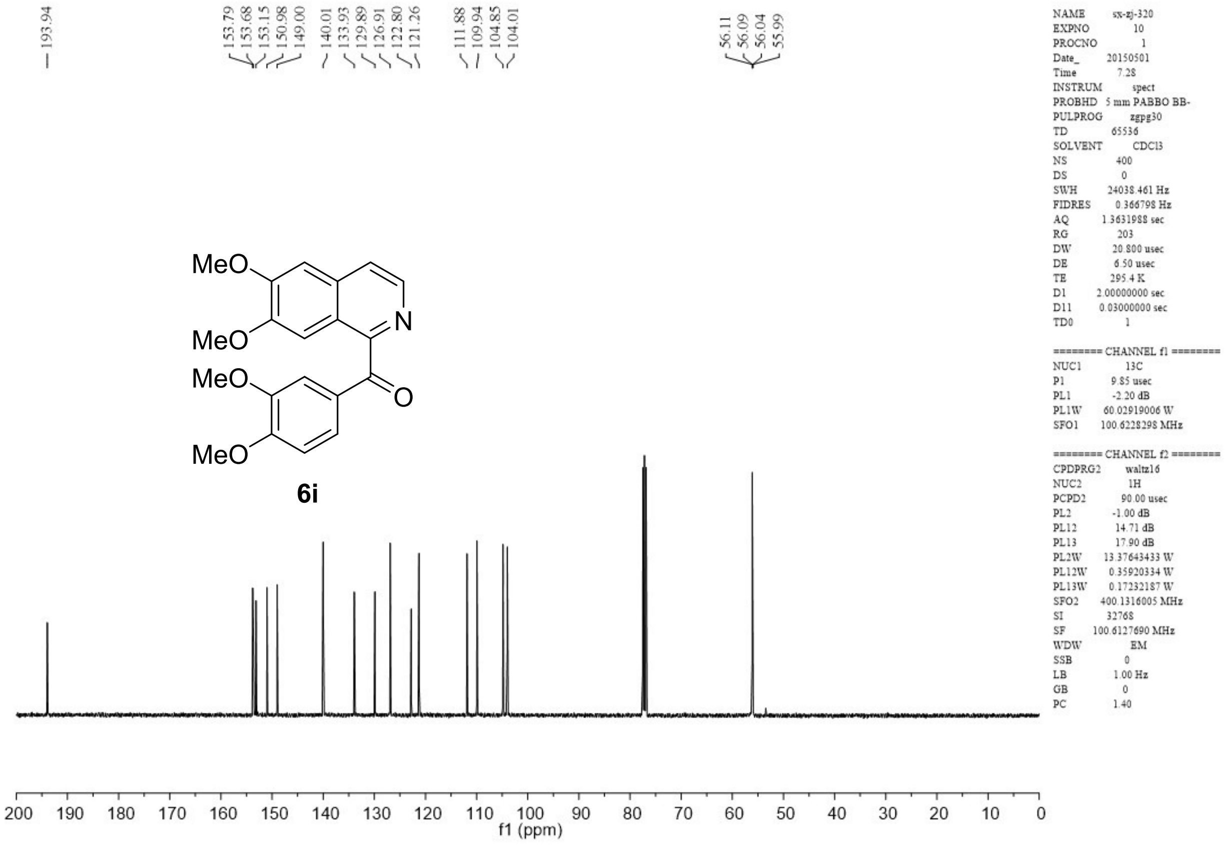

^{1}H NMR ($CDCl_3$, 400 MHz) spectrum of compound **6j**:

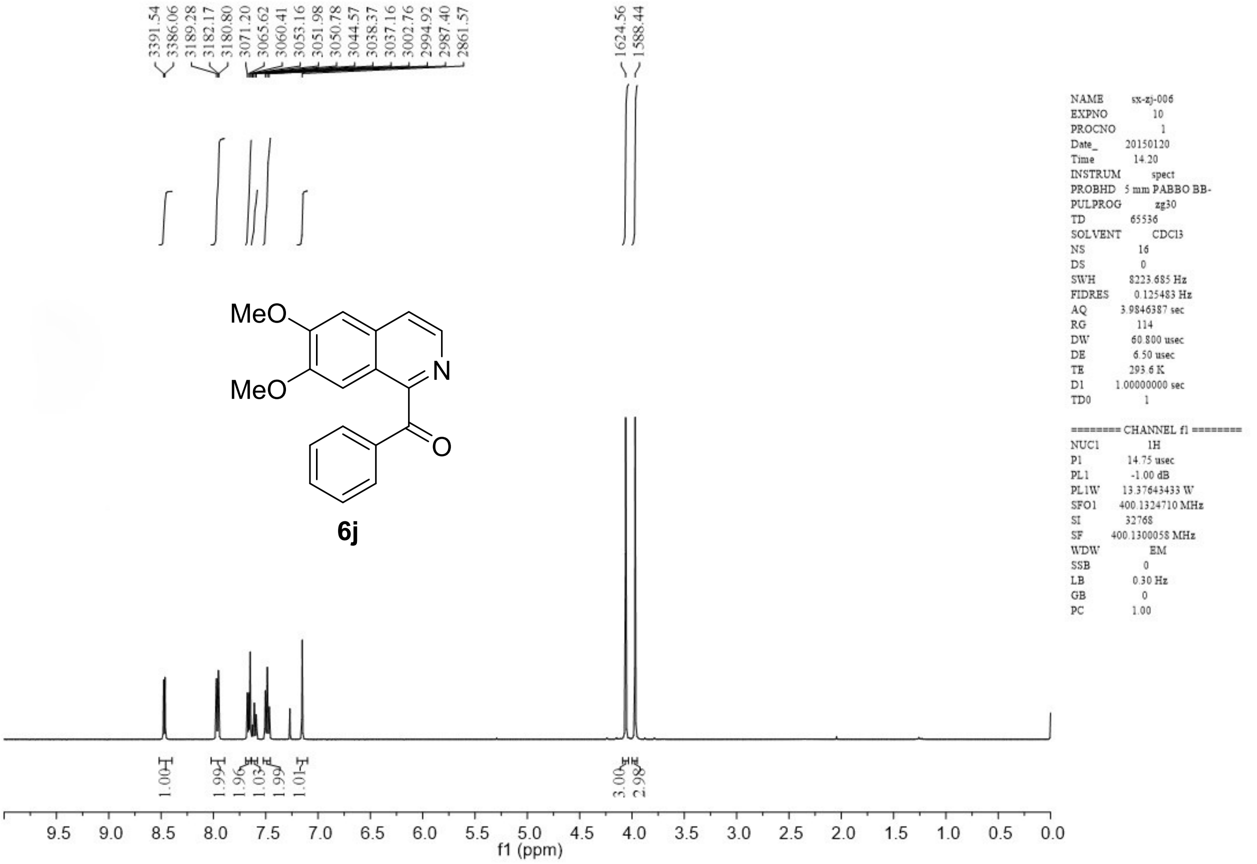

^{13}C NMR ($CDCl_3$, 100 MHz) spectrum of compound **6j**:

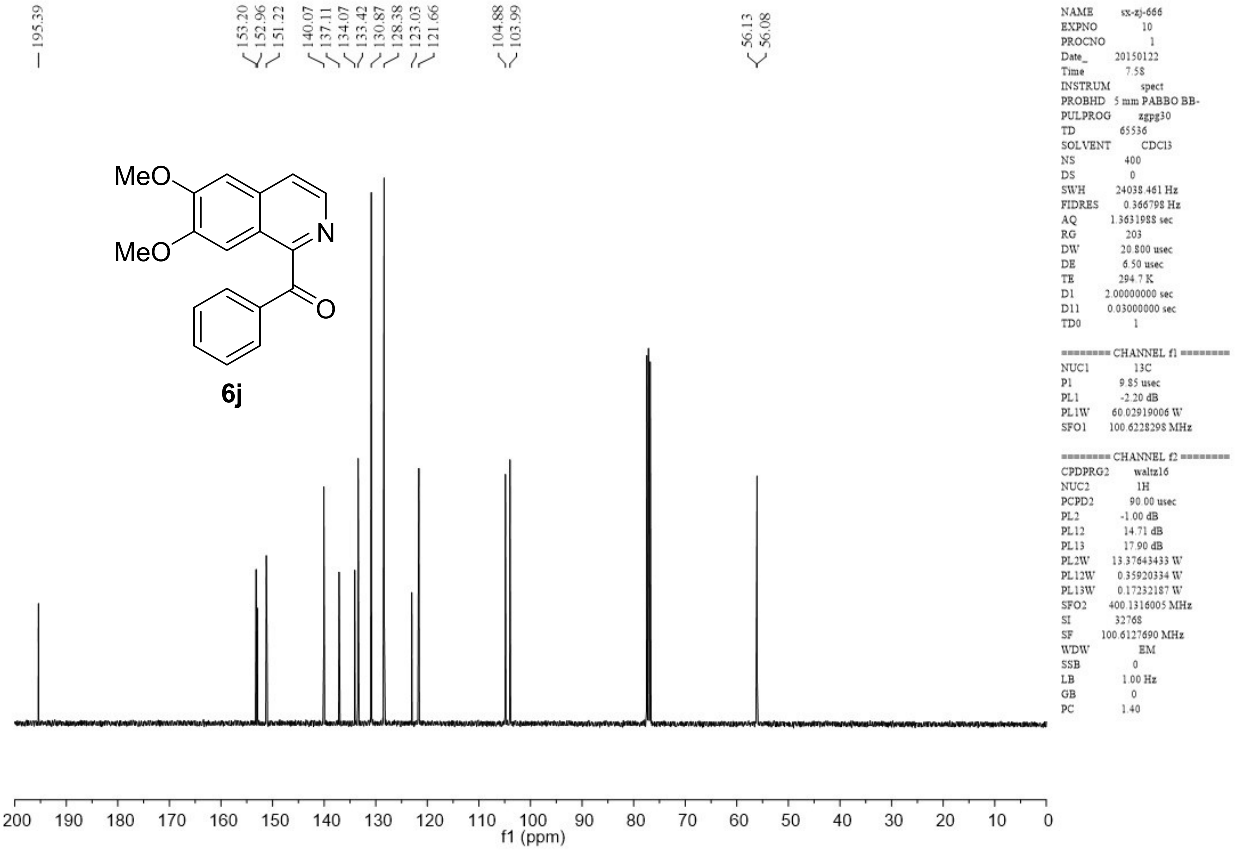

^{1}H NMR ($CDCl_3$, 400 MHz) spectrum of compound **6k**:

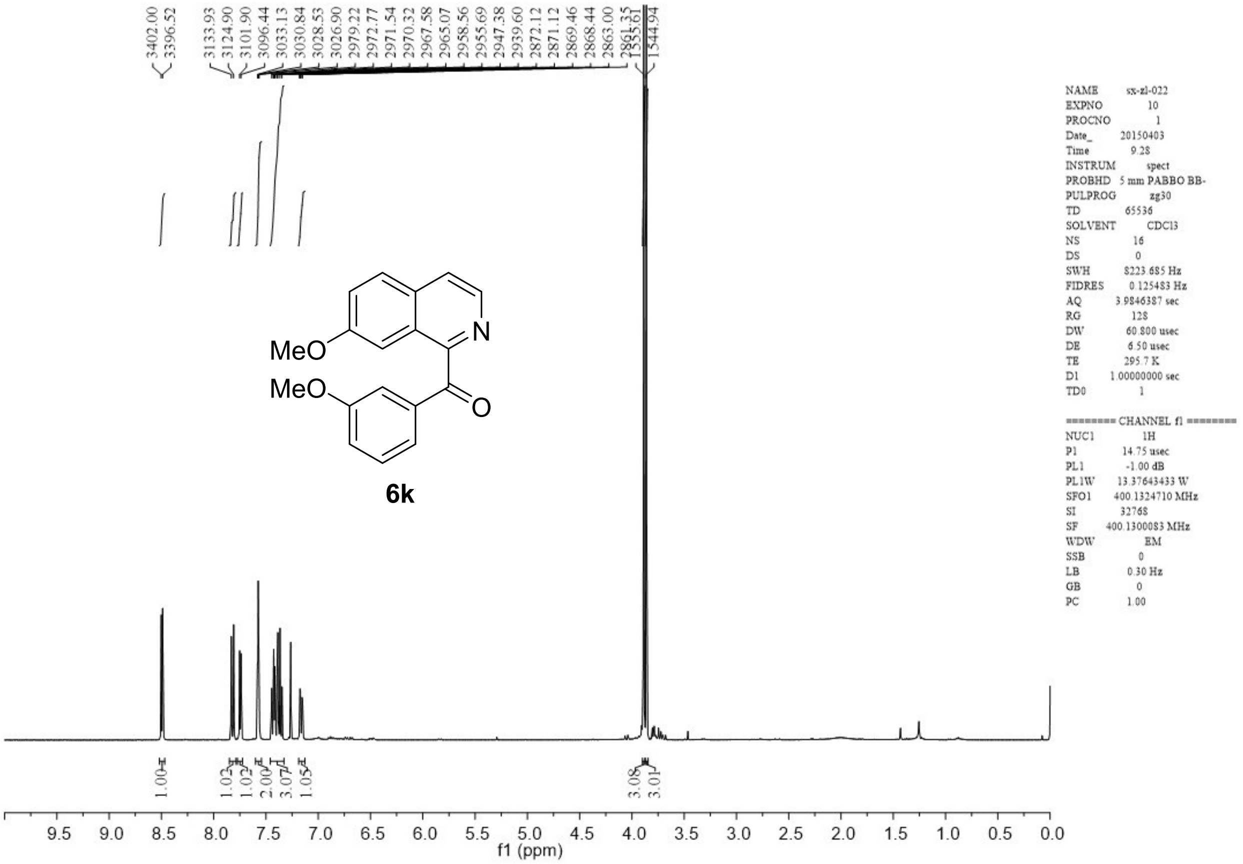

^{13}C NMR ($CDCl_3$, 100 MHz) spectrum of compound **6k**:

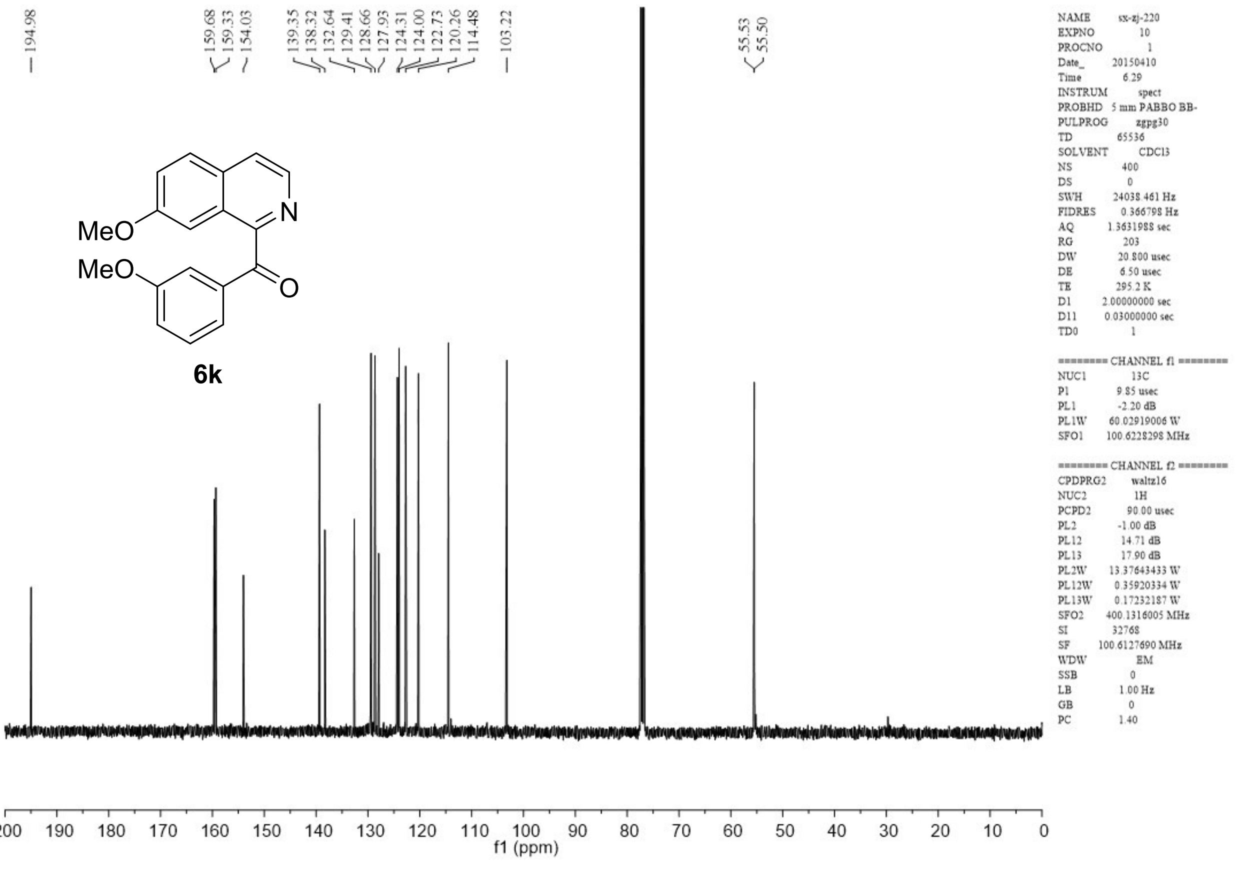

^{1}H NMR ($CDCl_3$, 400 MHz) spectrum of compound **6l**:

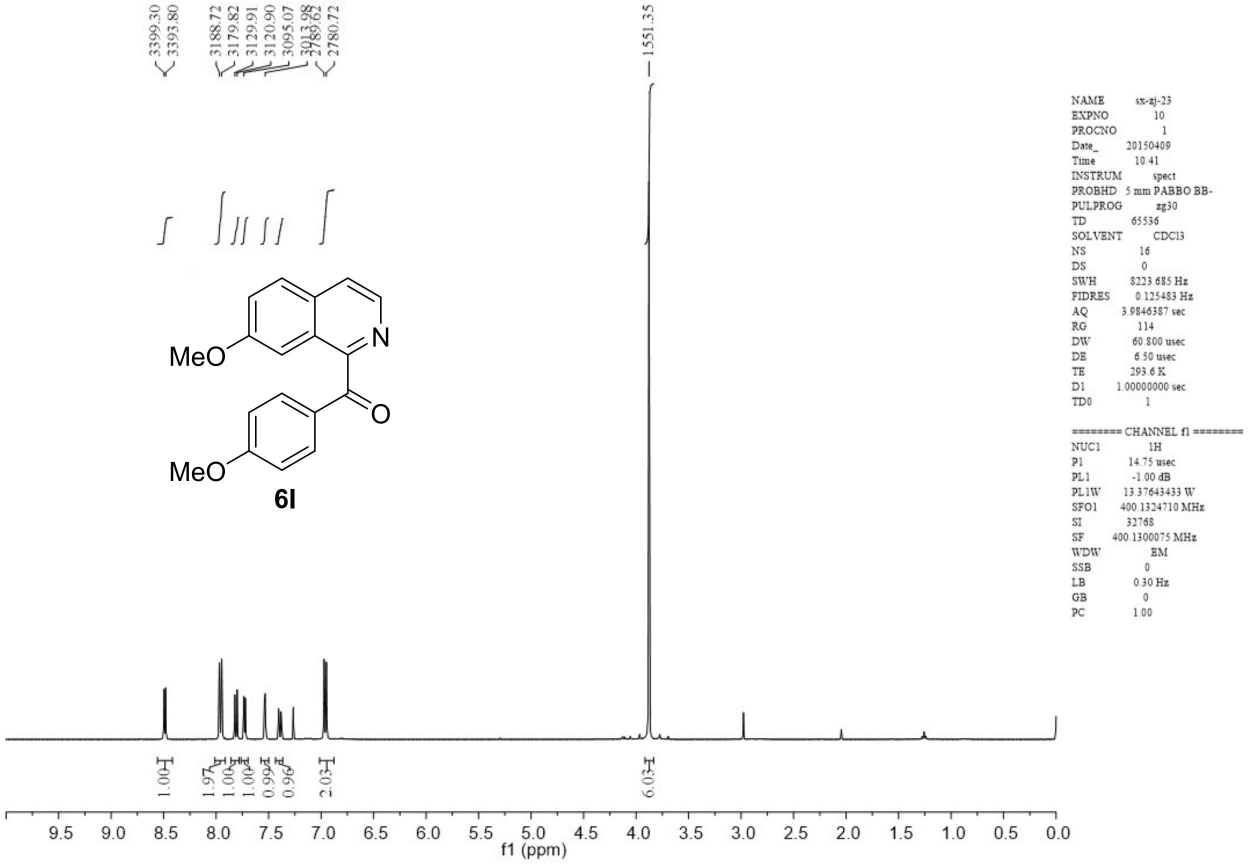

^{13}C NMR ($CDCl_3$, 100 MHz) spectrum of compound **6l**:

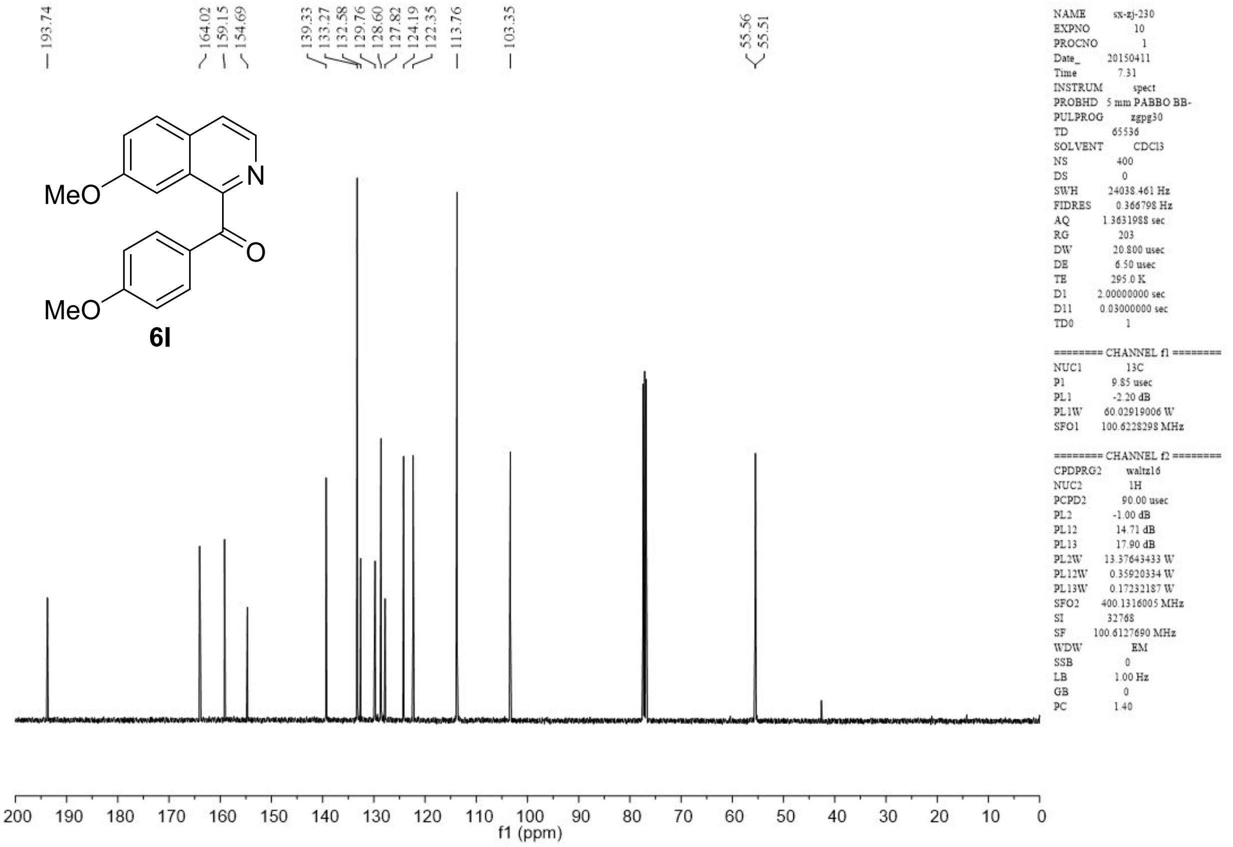

^1H NMR ($CDCl_3$, 400 MHz) spectrum of compound **6m**:

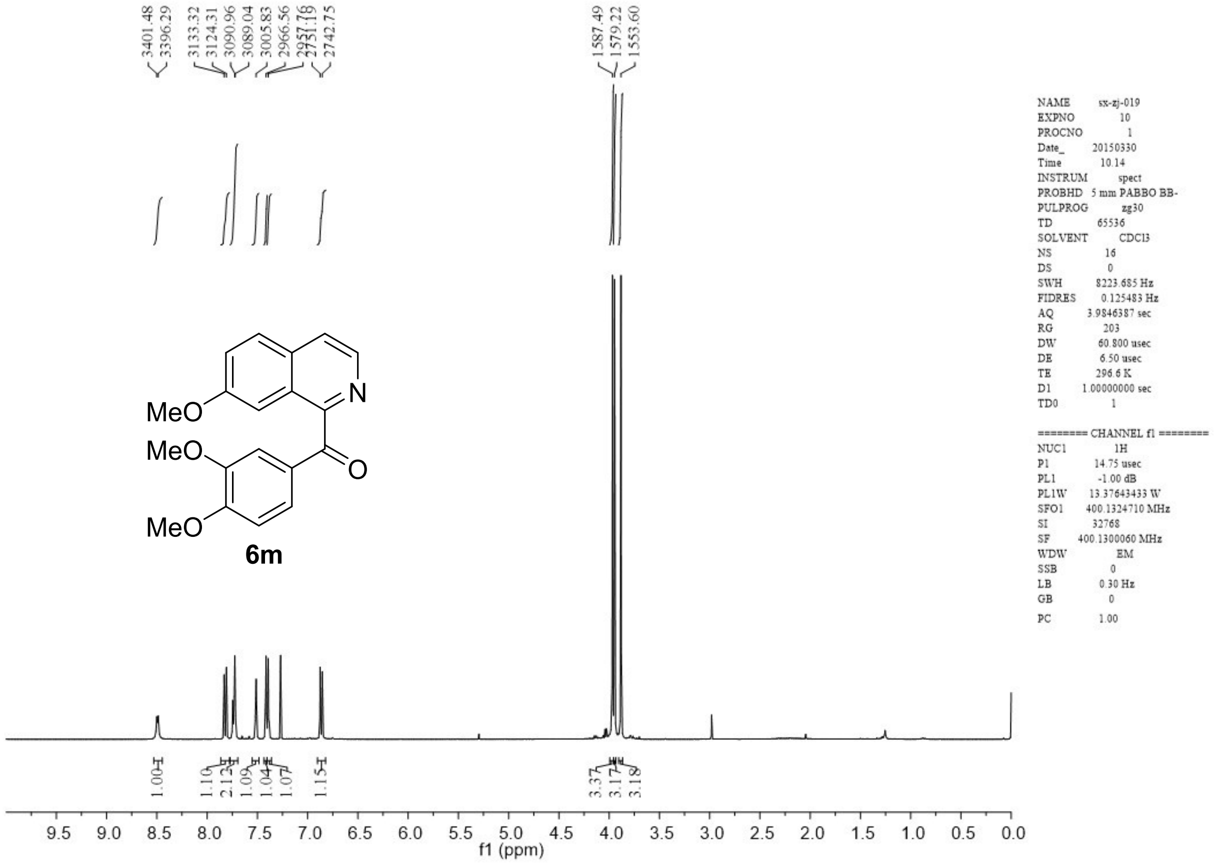

^{13}C NMR ($CDCl_3$, 100 MHz) spectrum of compound **6m**:

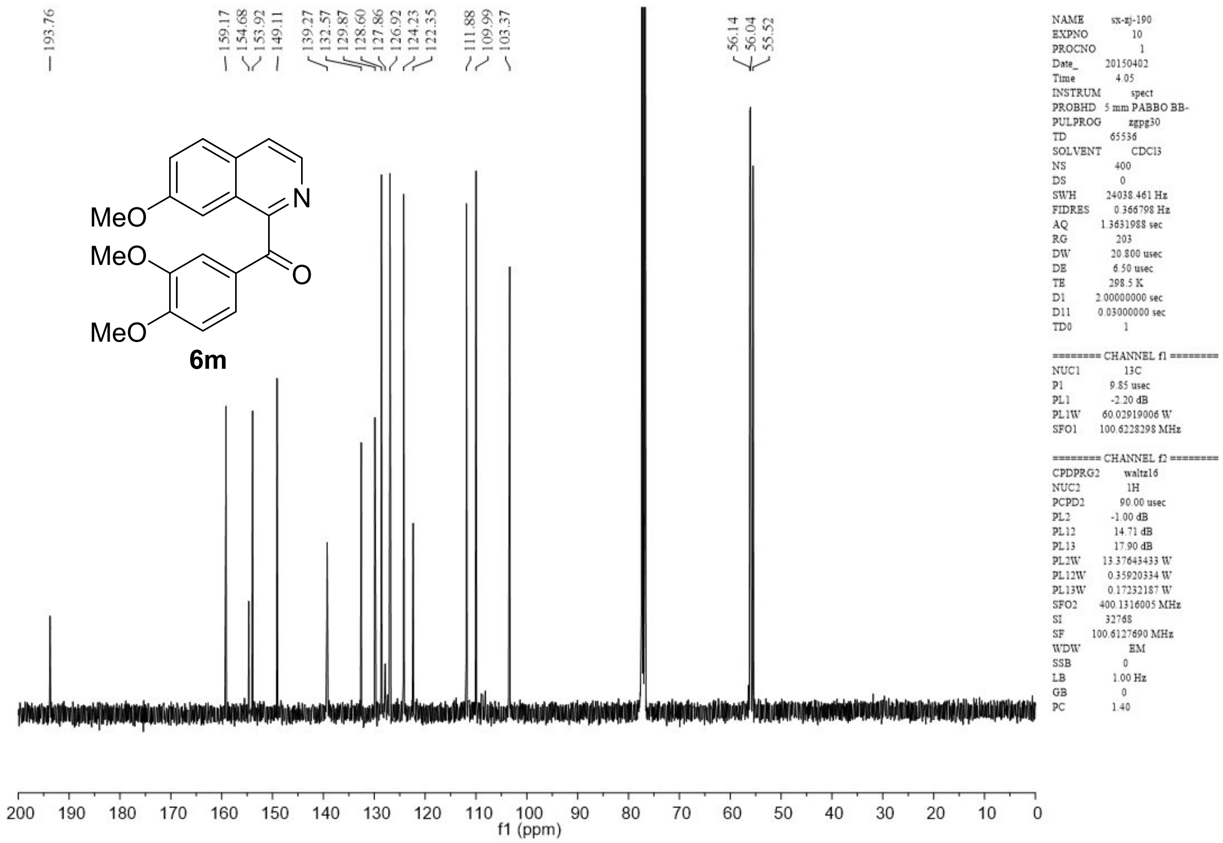

^{1}H NMR ($CDCl_3$, 400 MHz) spectrum of compound **6n**:

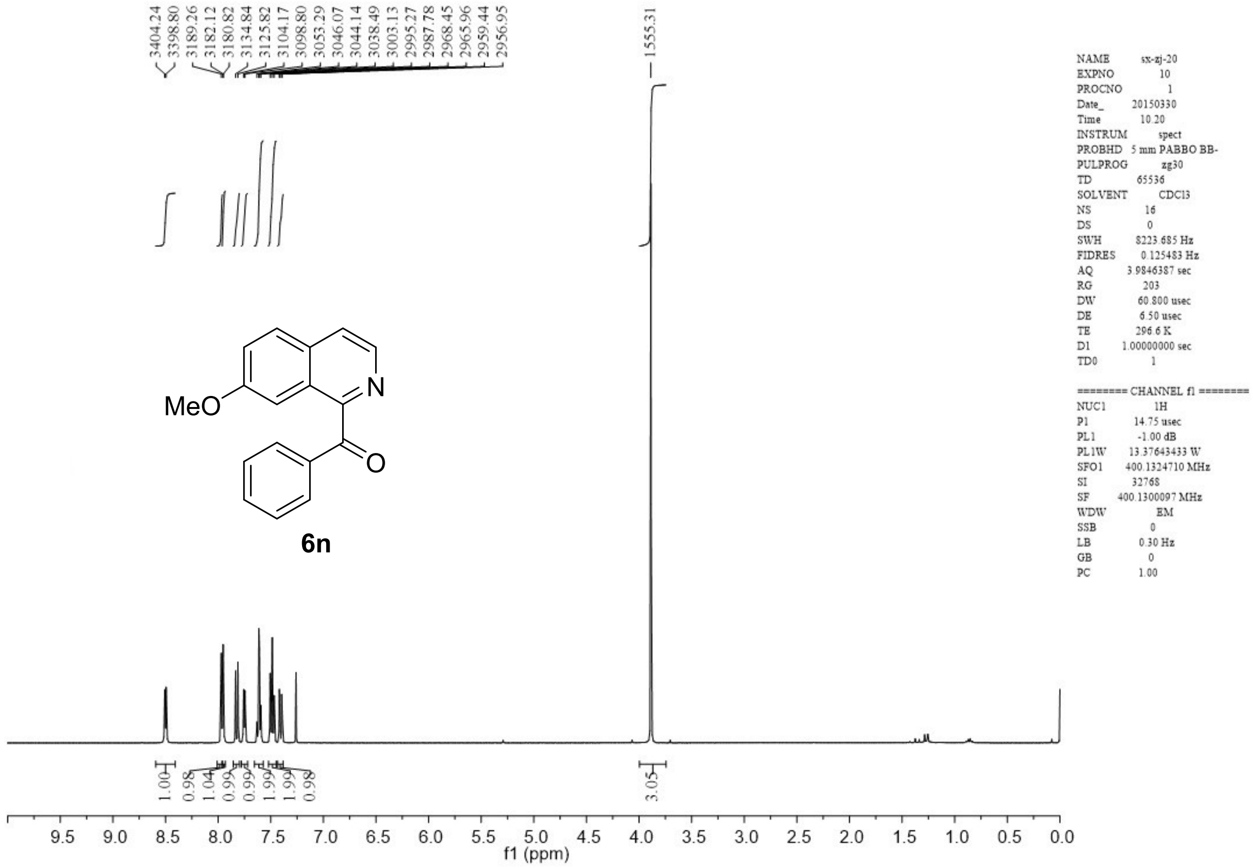

^{13}C NMR ($CDCl_3$, 100 MHz) spectrum of compound **6n**:

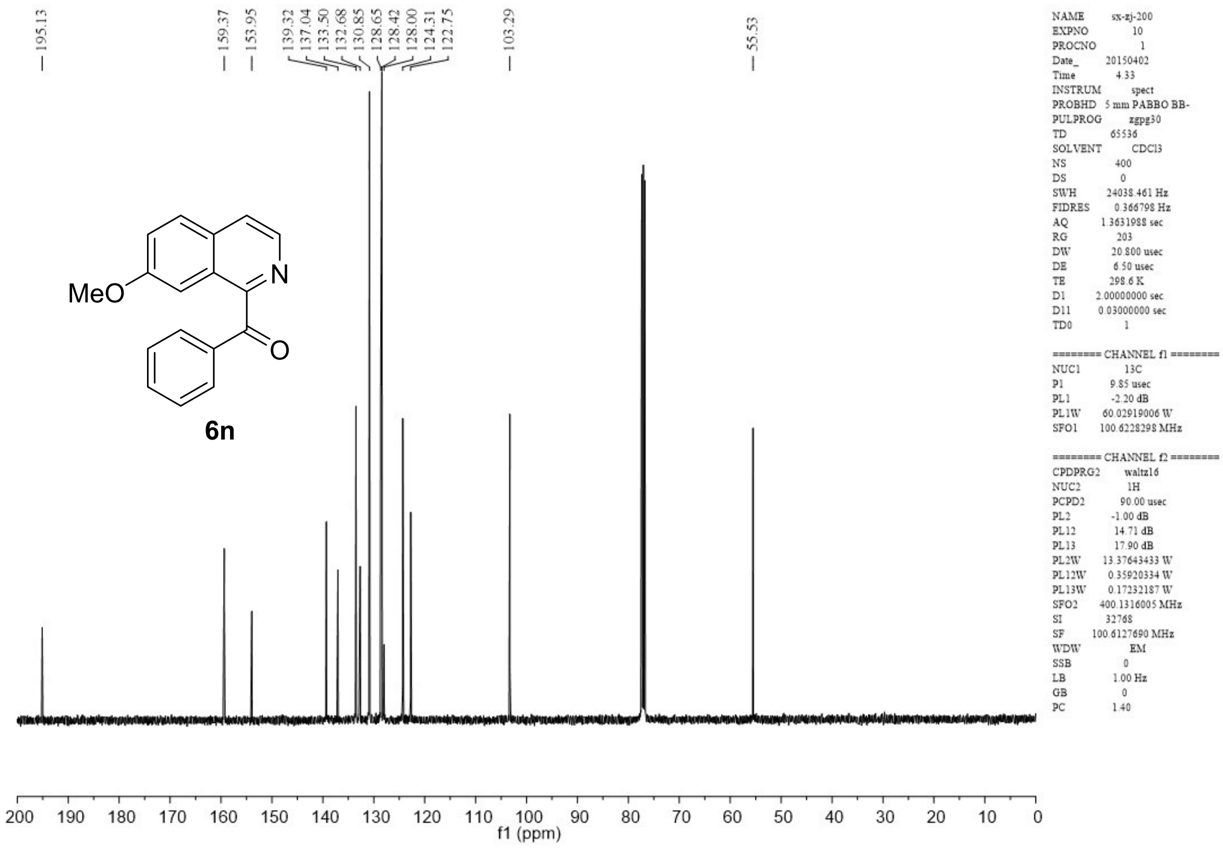

^{1}H NMR ($CDCl_3$, 400 MHz) spectrum of compound **6o**:

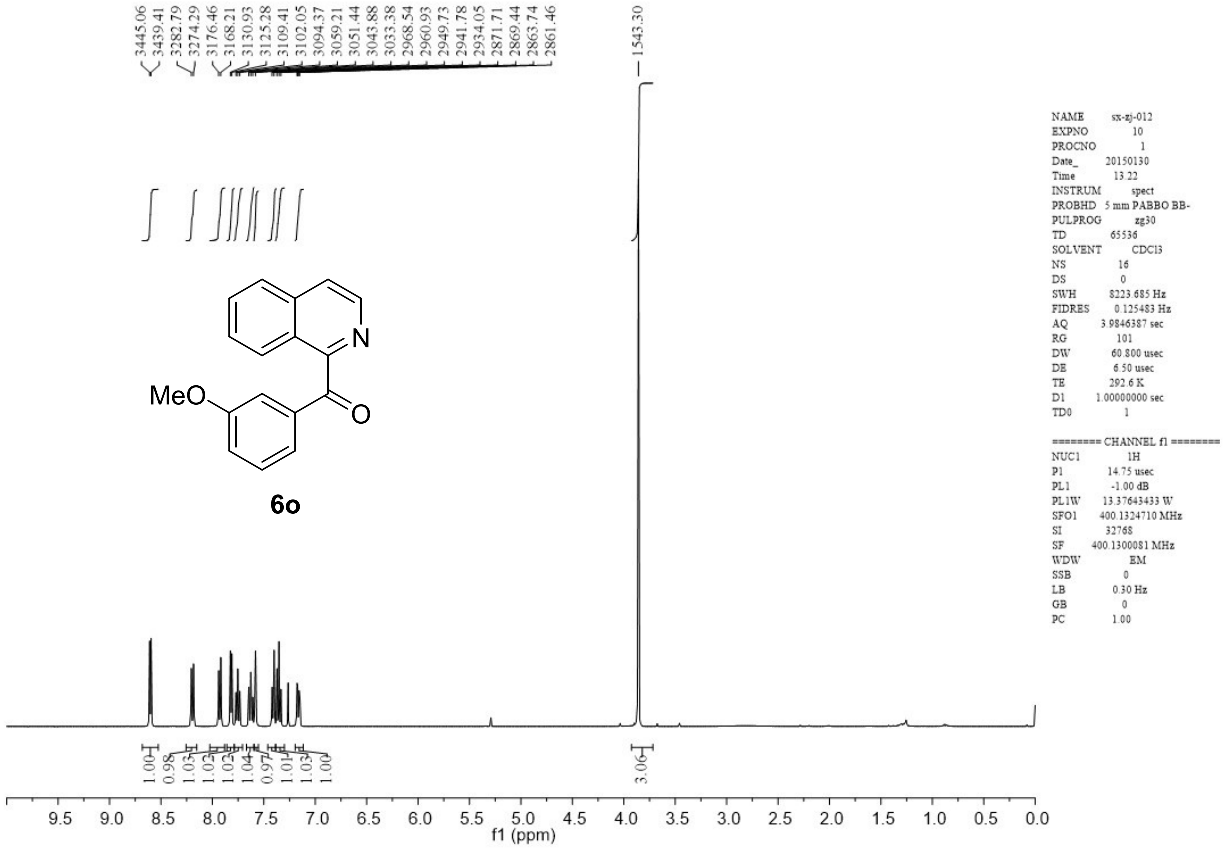

^{13}C NMR ($CDCl_3$, 100 MHz) spectrum of compound **6o**:

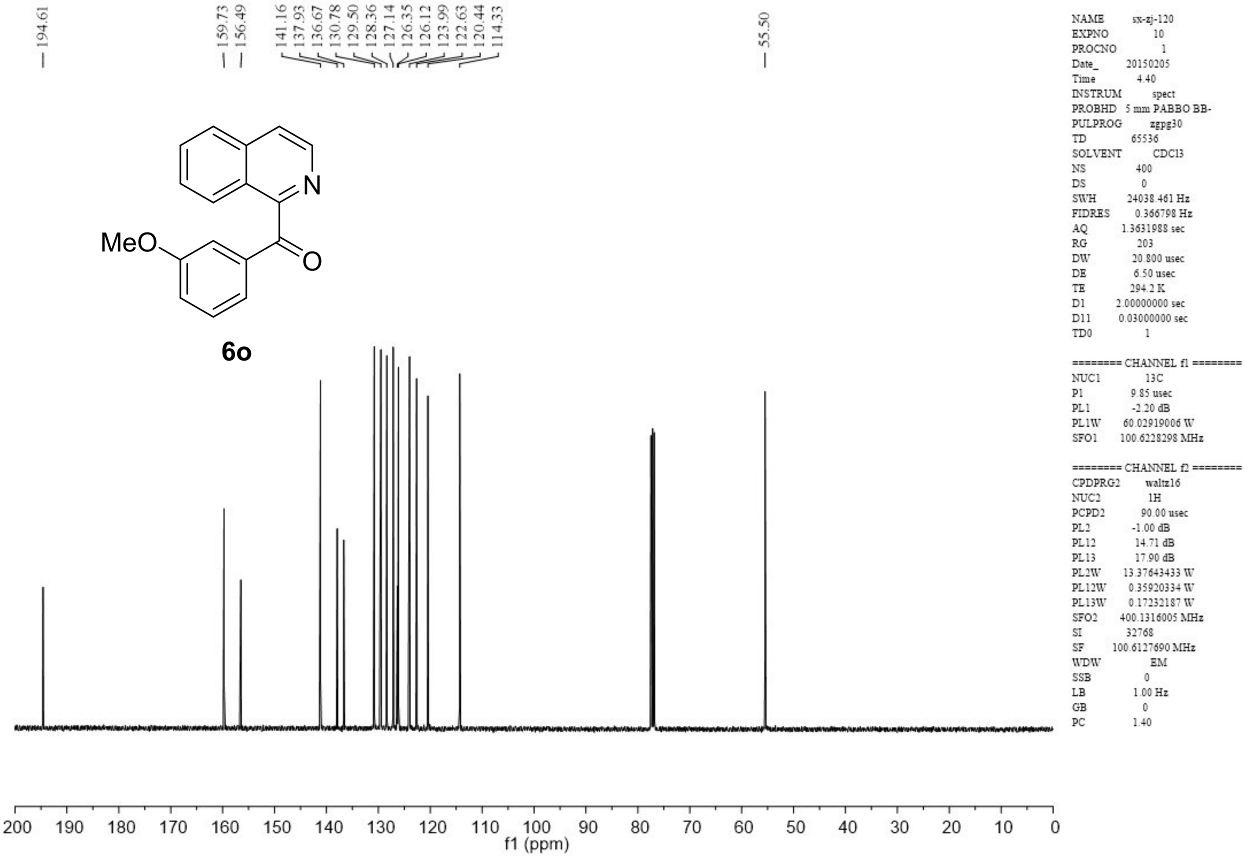

^{1}H NMR ($CDCl_3$, 400 MHz) spectrum of compound **6p**:

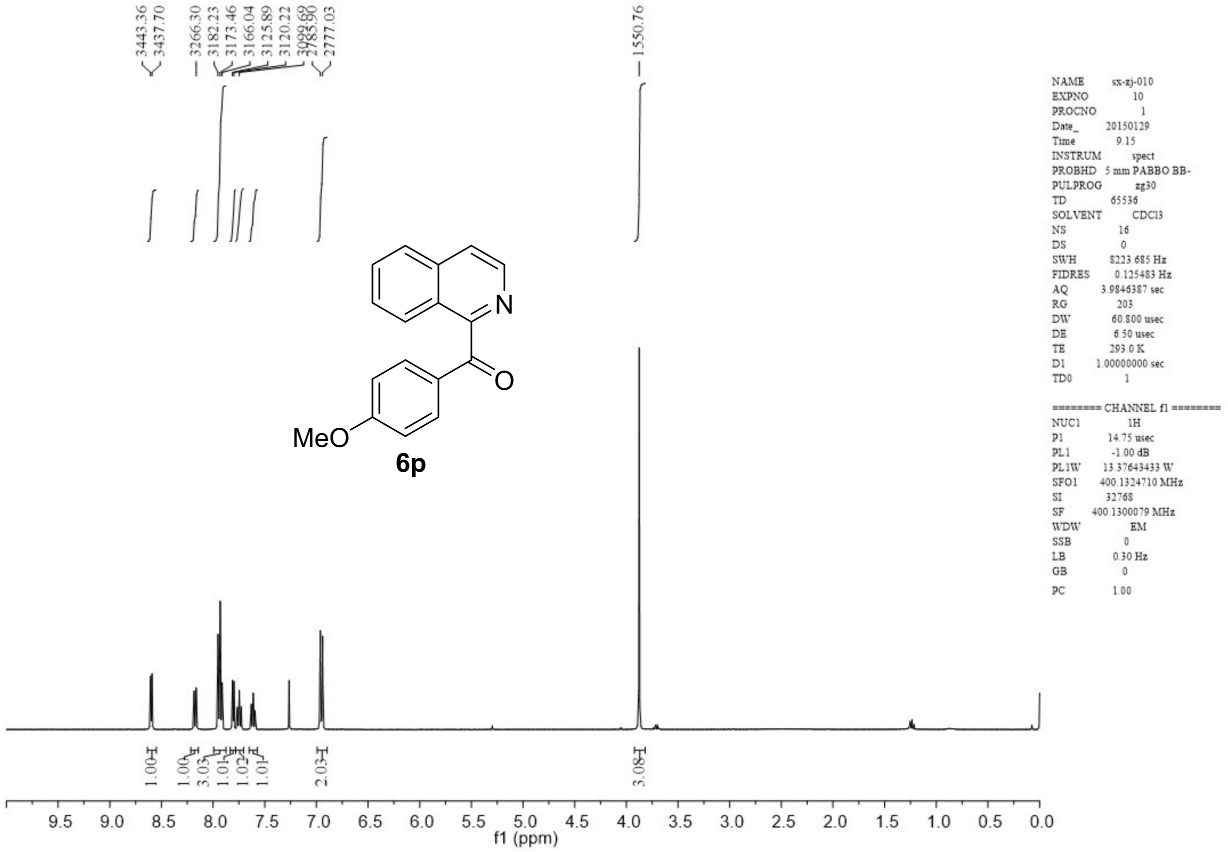

^{13}C NMR ($CDCl_3$, 100 MHz) spectrum of compound **6p**:

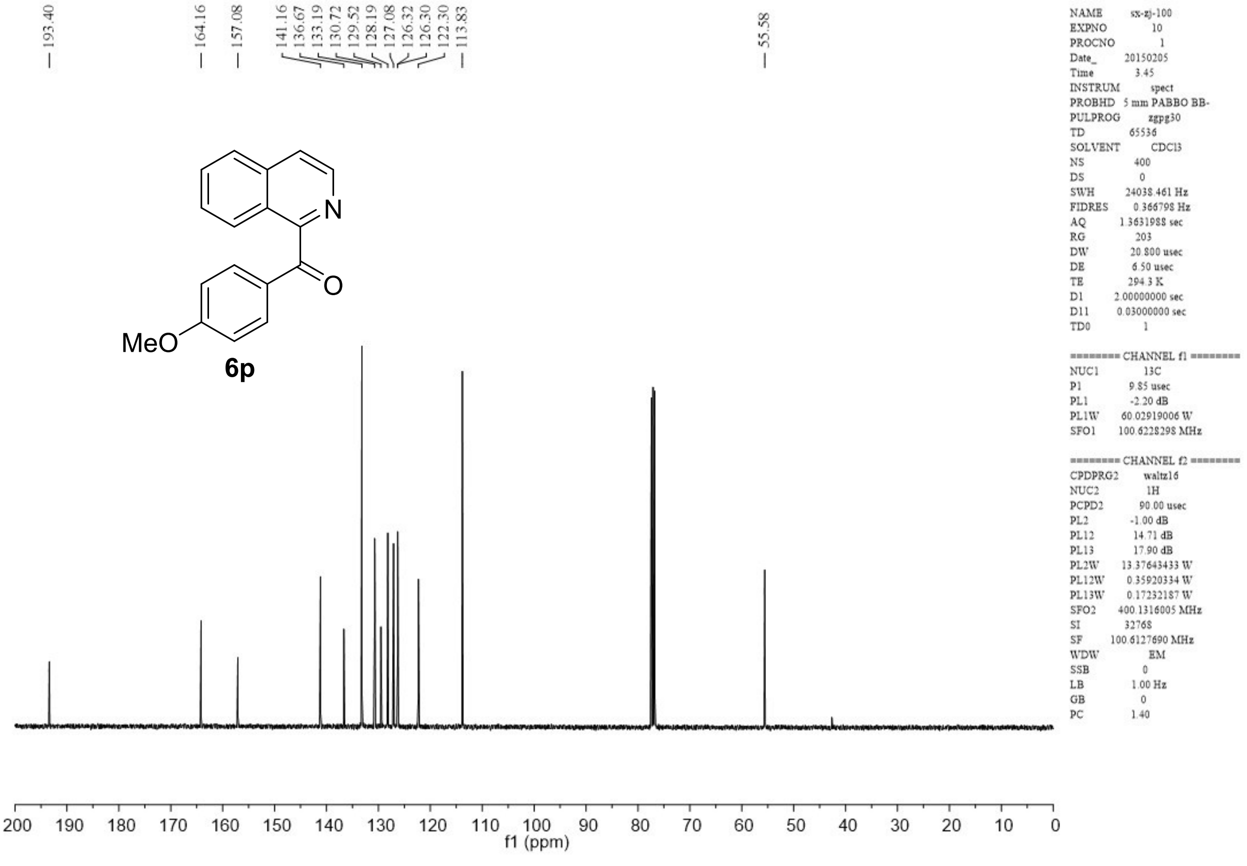

^{1}H NMR ($CDCl_3$, 400 MHz) spectrum of compound **6q**:

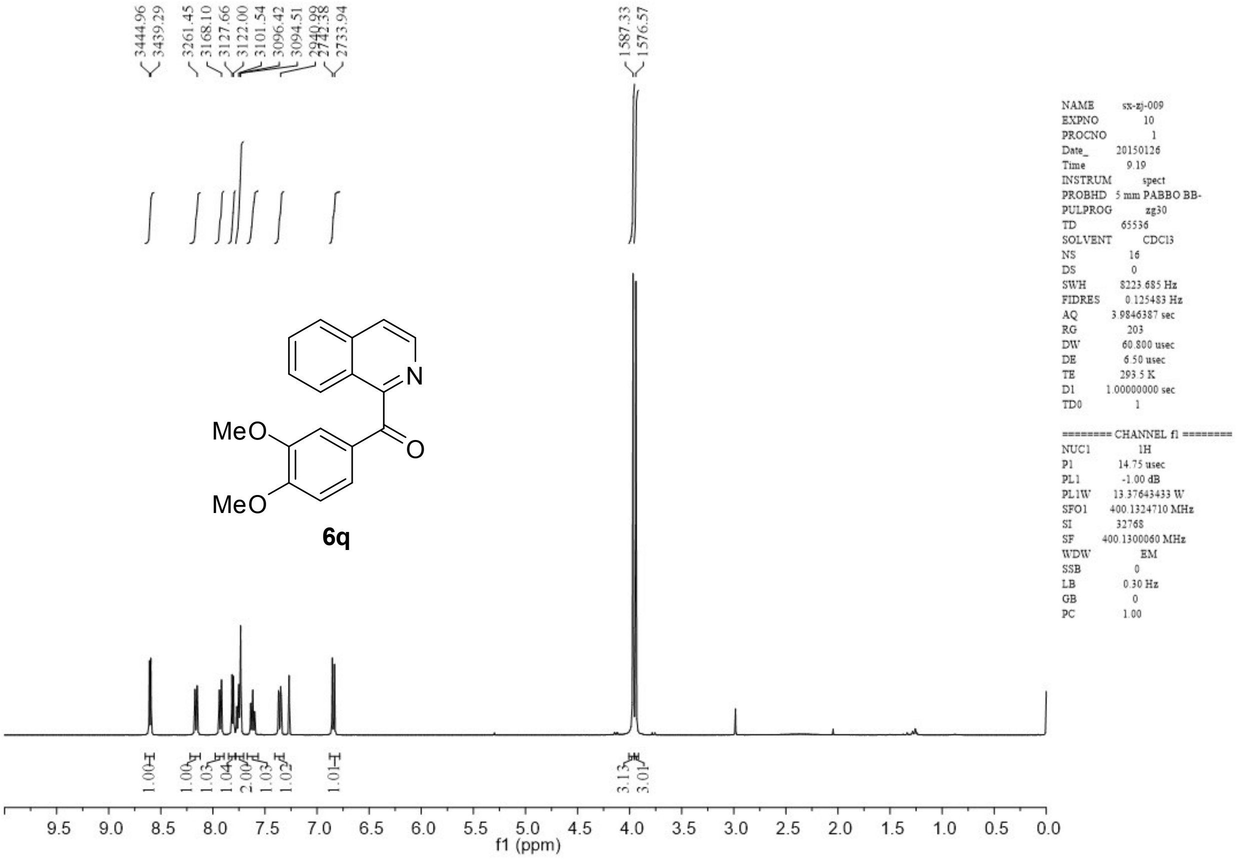

^{13}C NMR ($CDCl_3$, 100 MHz) spectrum of compound **6q**:

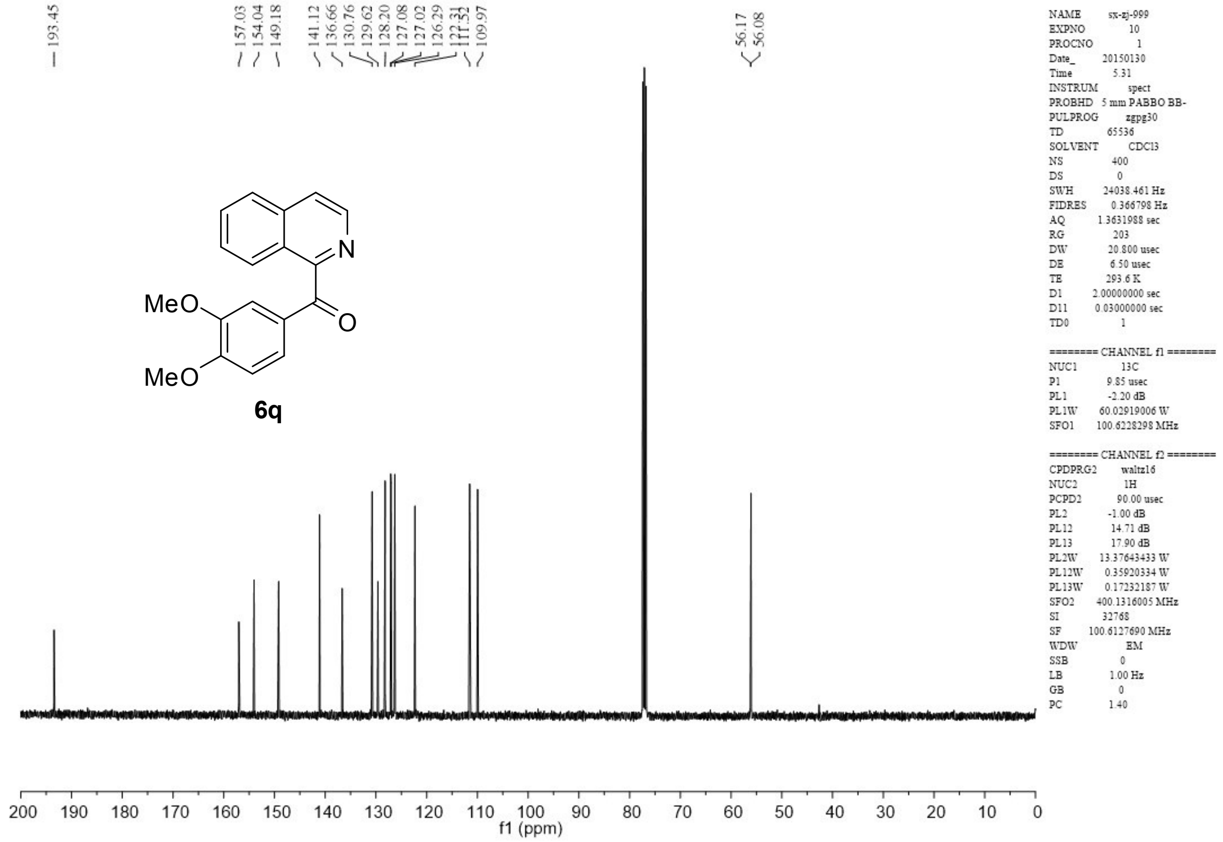

^{1}H NMR ($CDCl_3$, 400 MHz) spectrum of compound **6r**:

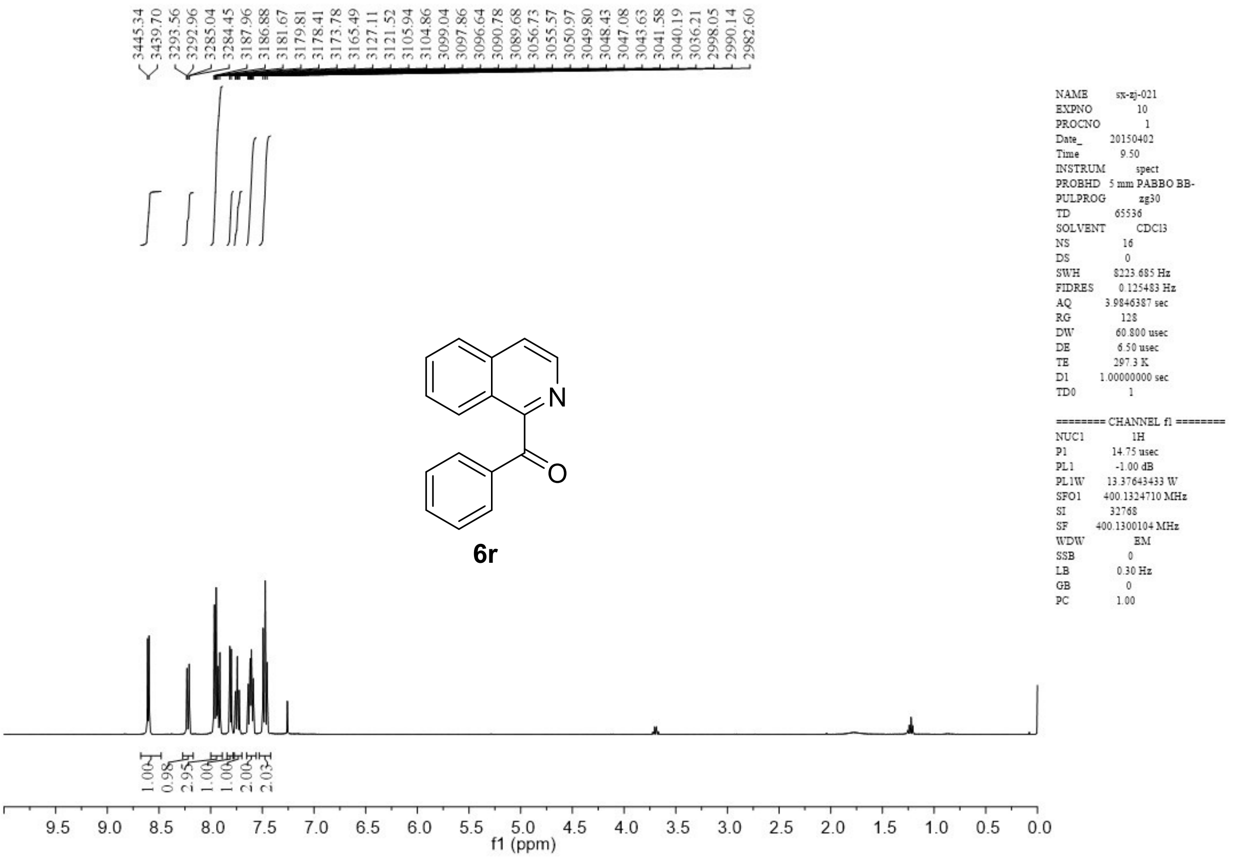

^{13}C NMR ($CDCl_3$, 100 MHz) spectrum of compound **6r**:

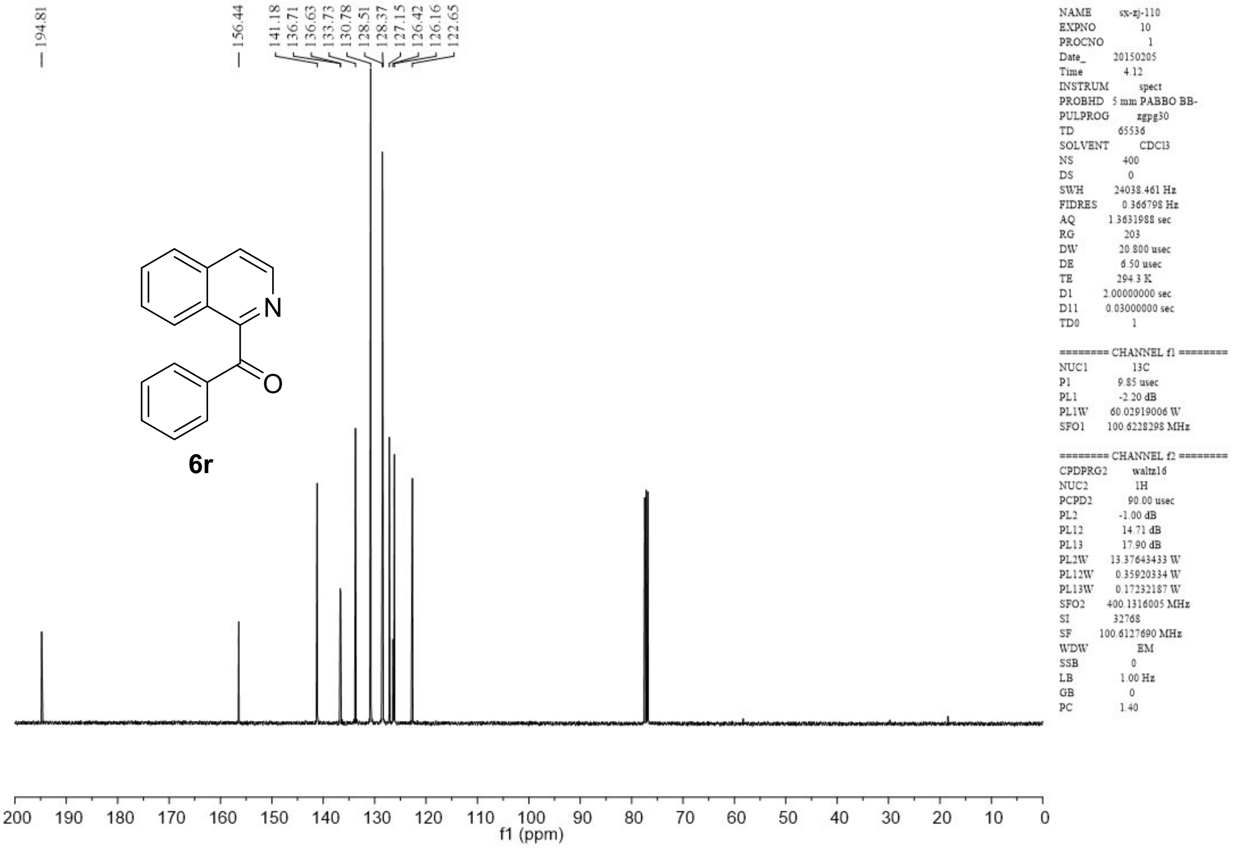

^{1}H NMR ($CDCl_3$, 400 MHz) spectrum of compound **6s**:

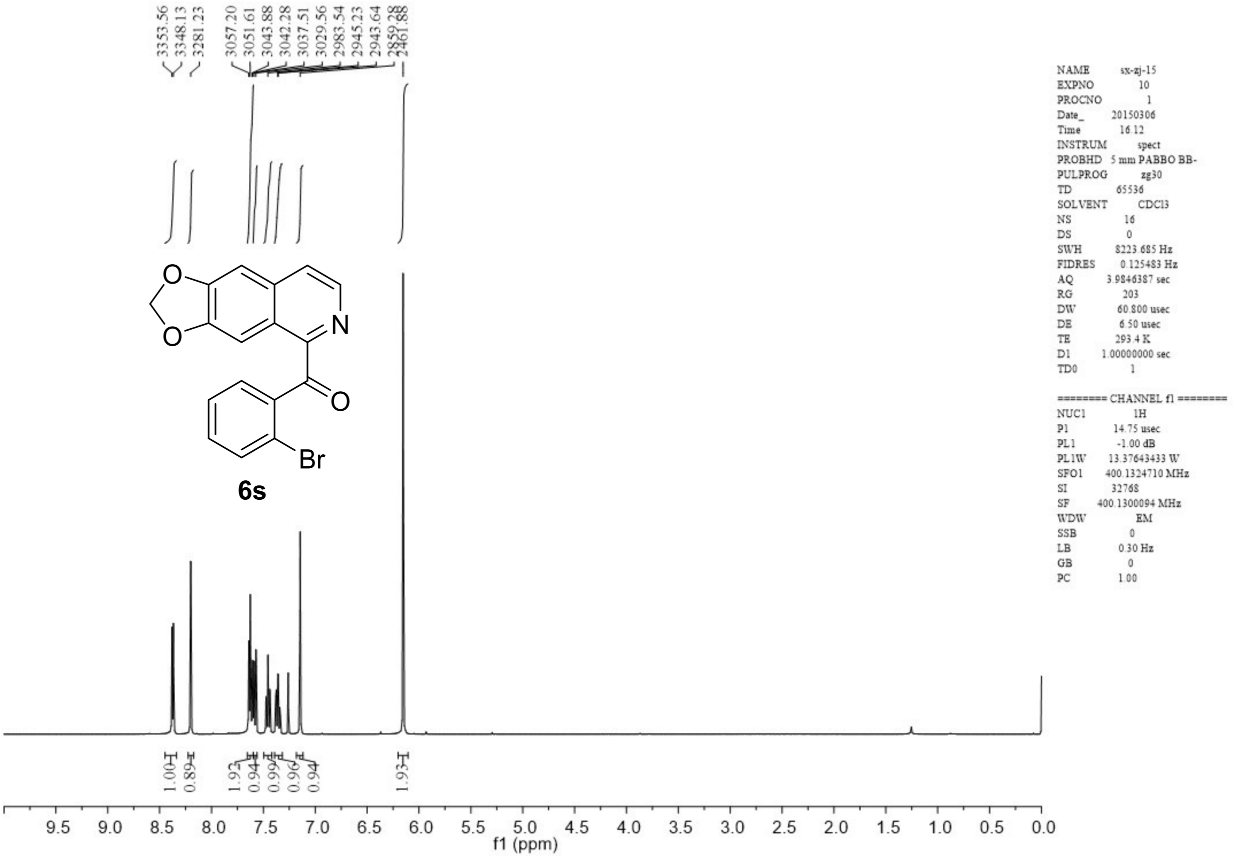

^{13}C NMR ($CDCl_3$, 100 MHz) spectrum of compound **6s**:

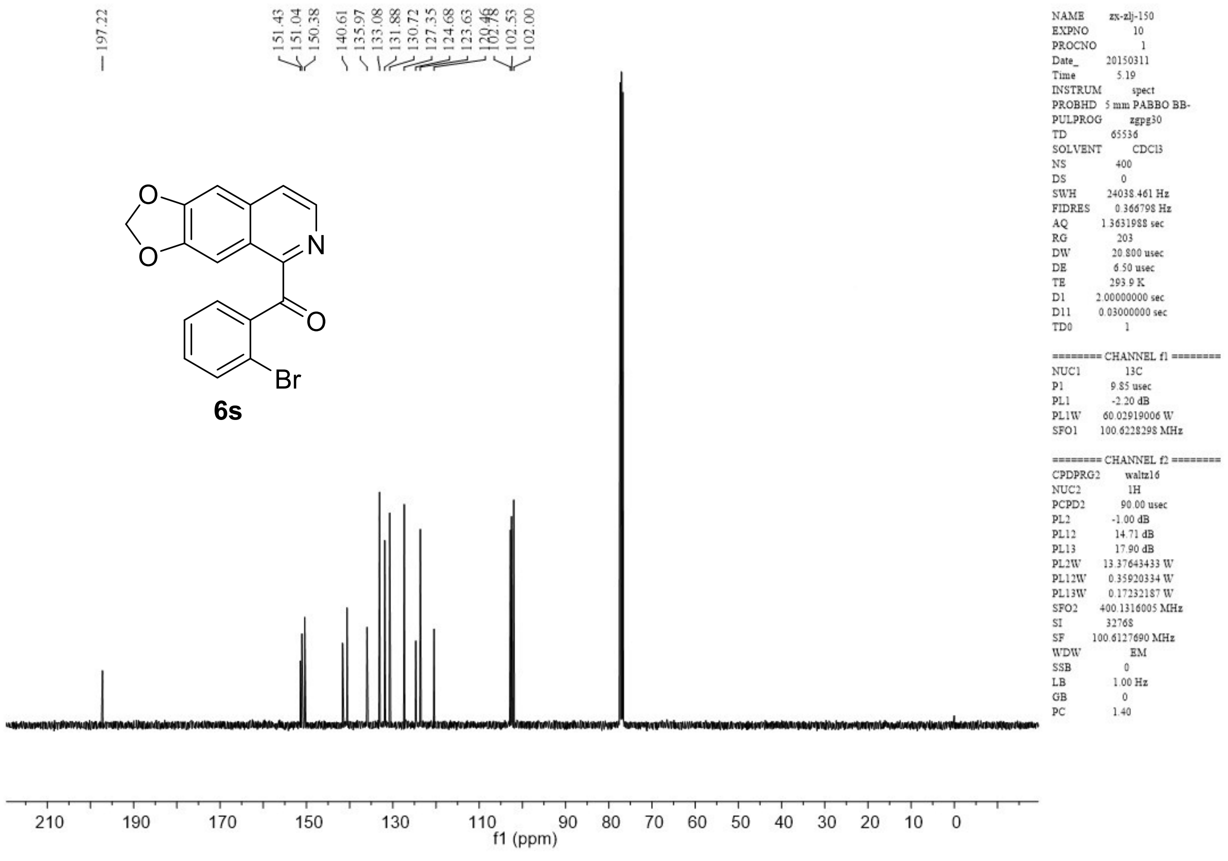

^{1}H NMR ($CDCl_3$, 400 MHz) spectrum of compound **6t**:

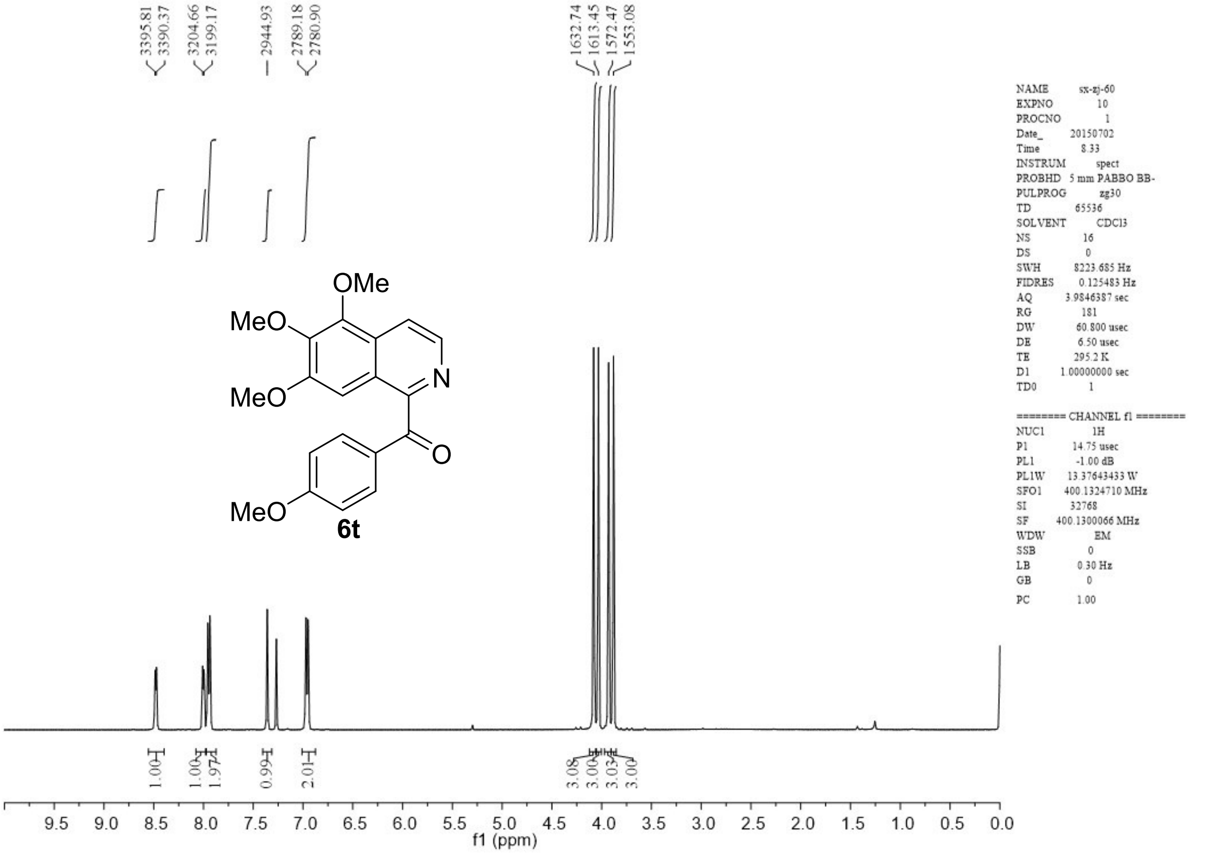

^{13}C NMR ($CDCl_3$, 100 MHz) spectrum of compound **6t**:

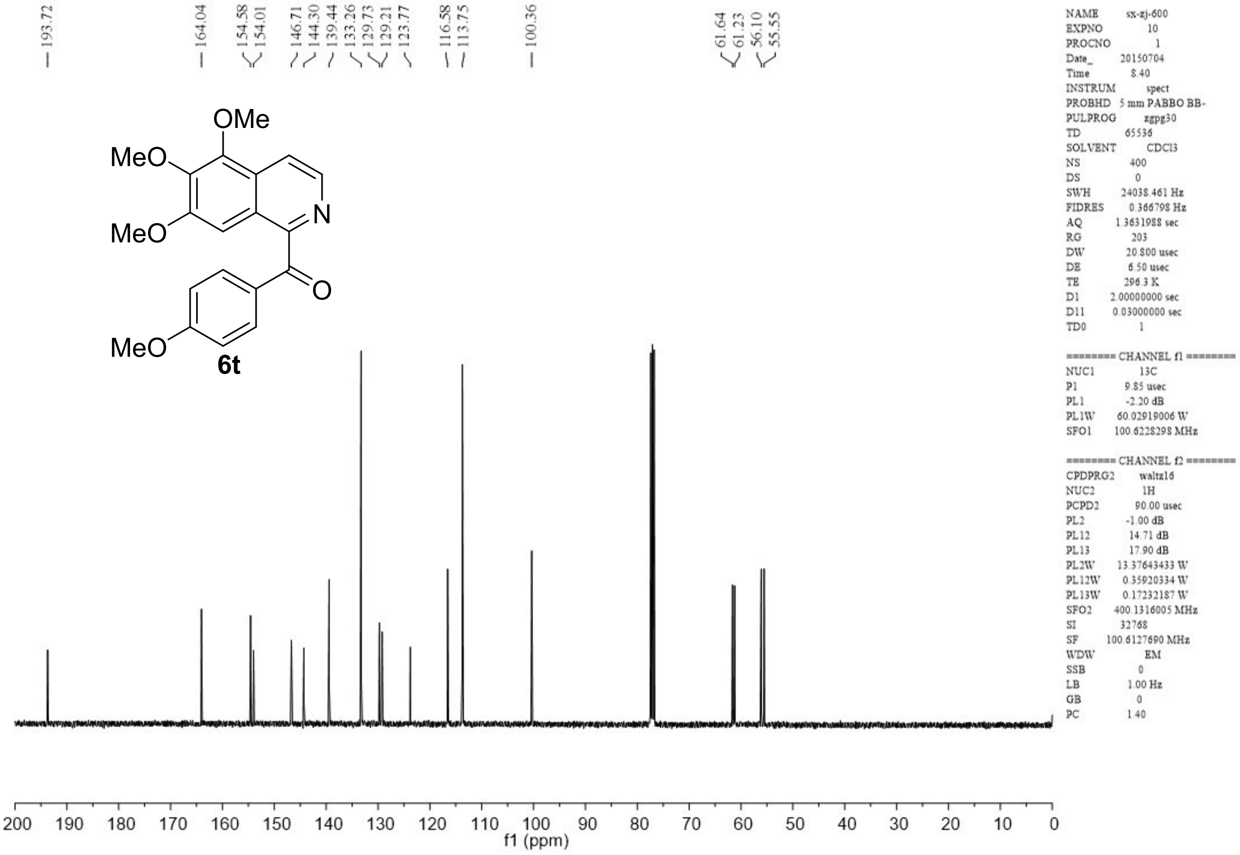

^{1}H NMR (DMSO-d_6, 400 MHz) spectrum of compound **7a**:

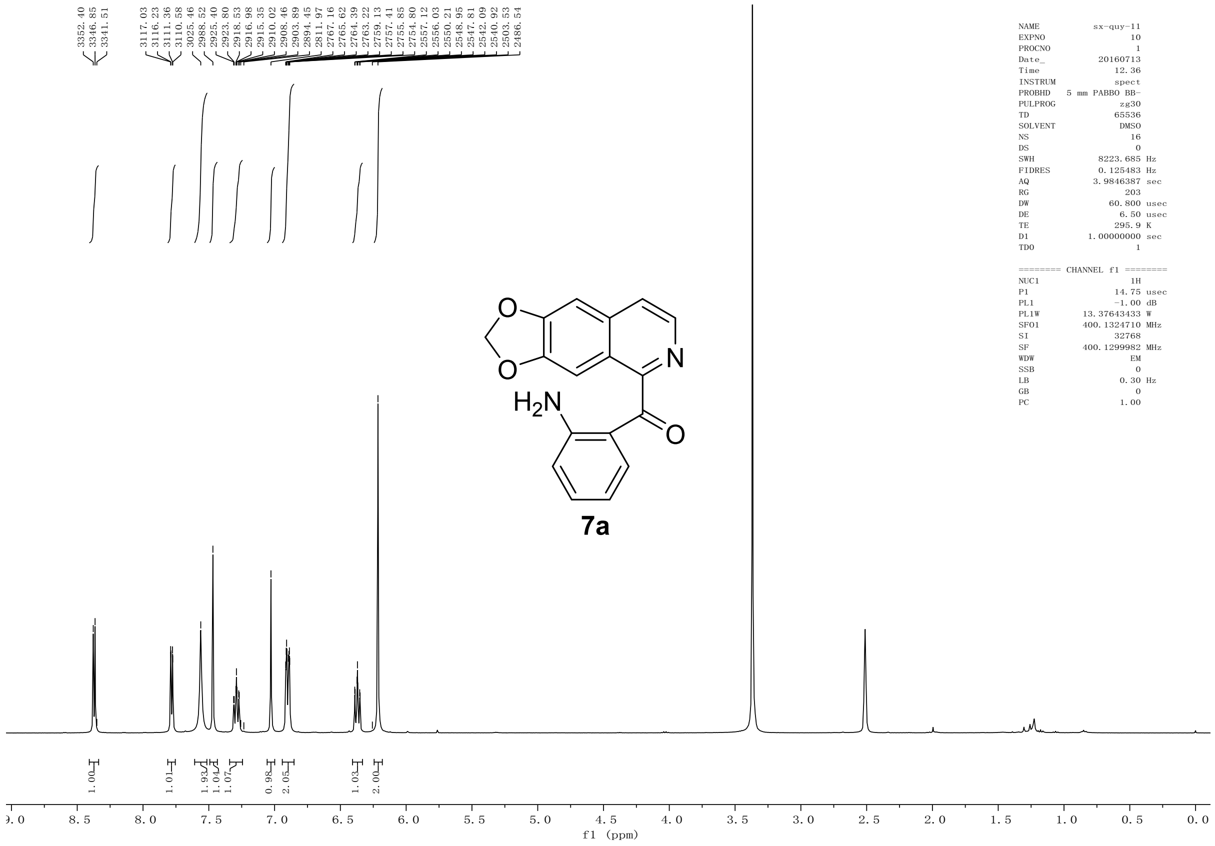

^{13}C NMR (DMSO-d_6, 100 MHz) spectrum of compound **7a**:

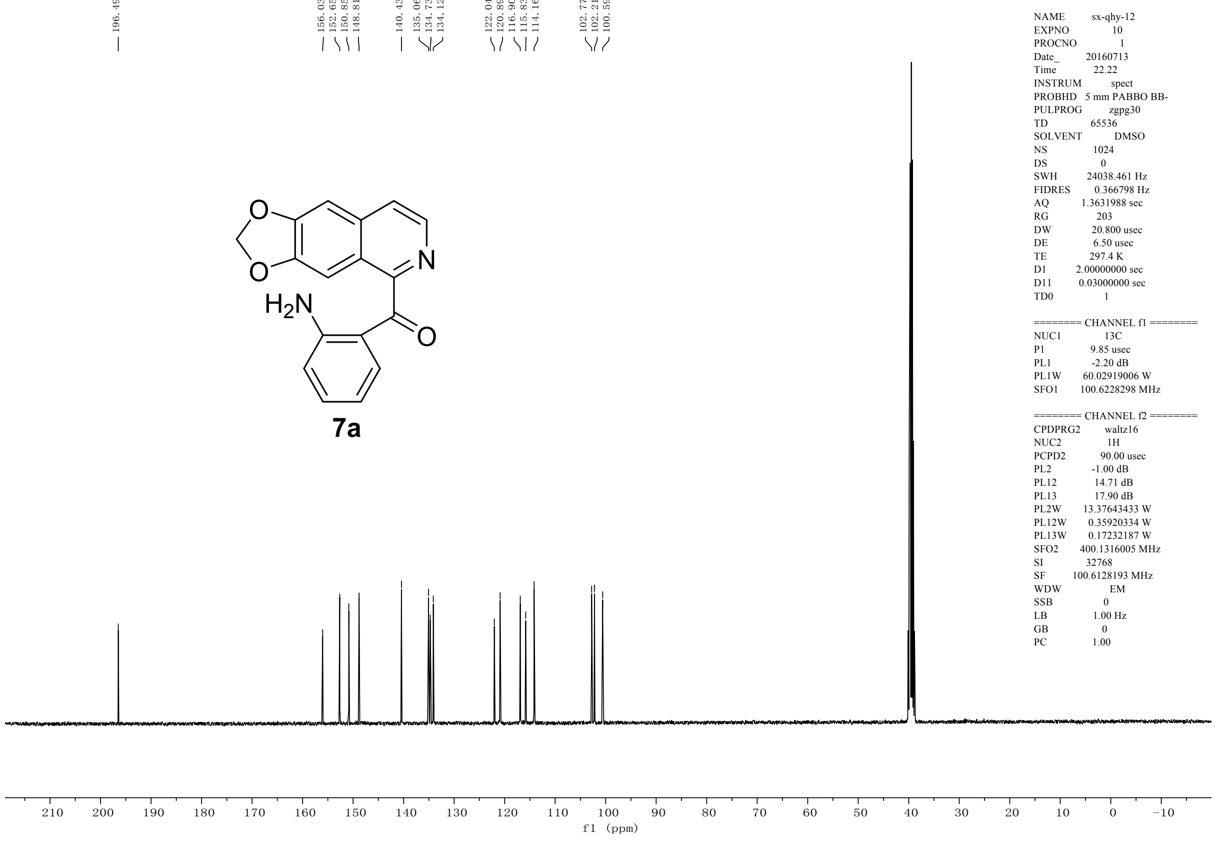

^{1}H NMR (DMSO-d_6, 400 MHz) spectrum of compound **7b**:

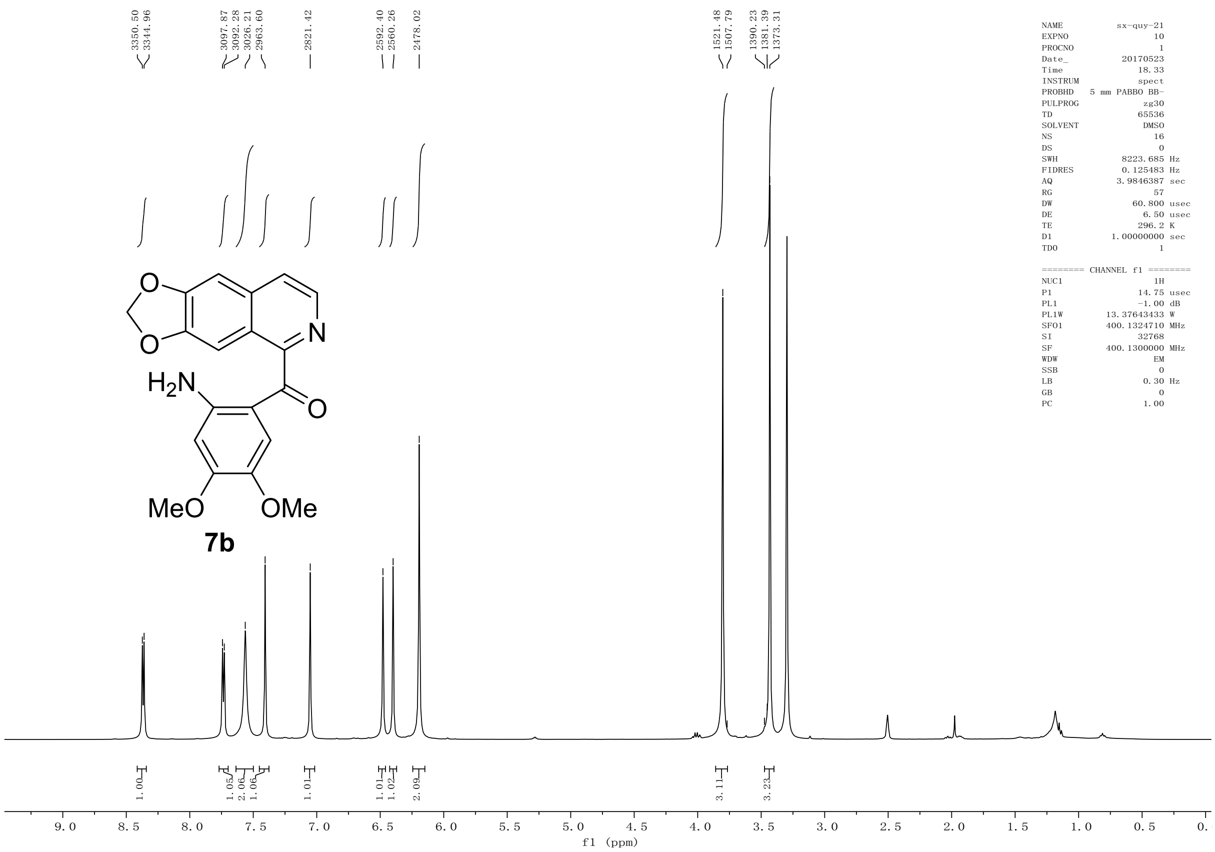

^{13}C NMR (DMSO-d_6, 100 MHz) spectrum of compound **7b**:

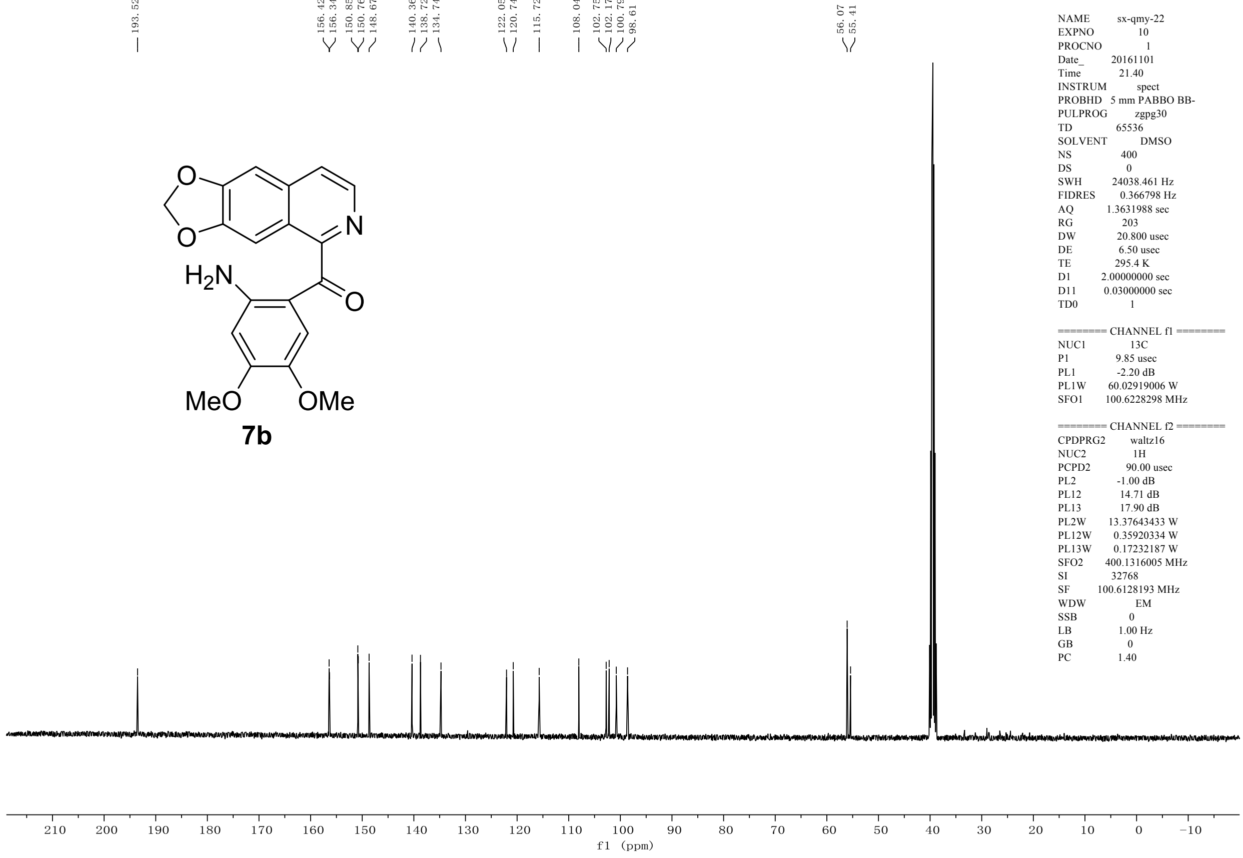

^{1}H NMR (DMSO-d6, 400 MHz) spectrum of compound **7c**:

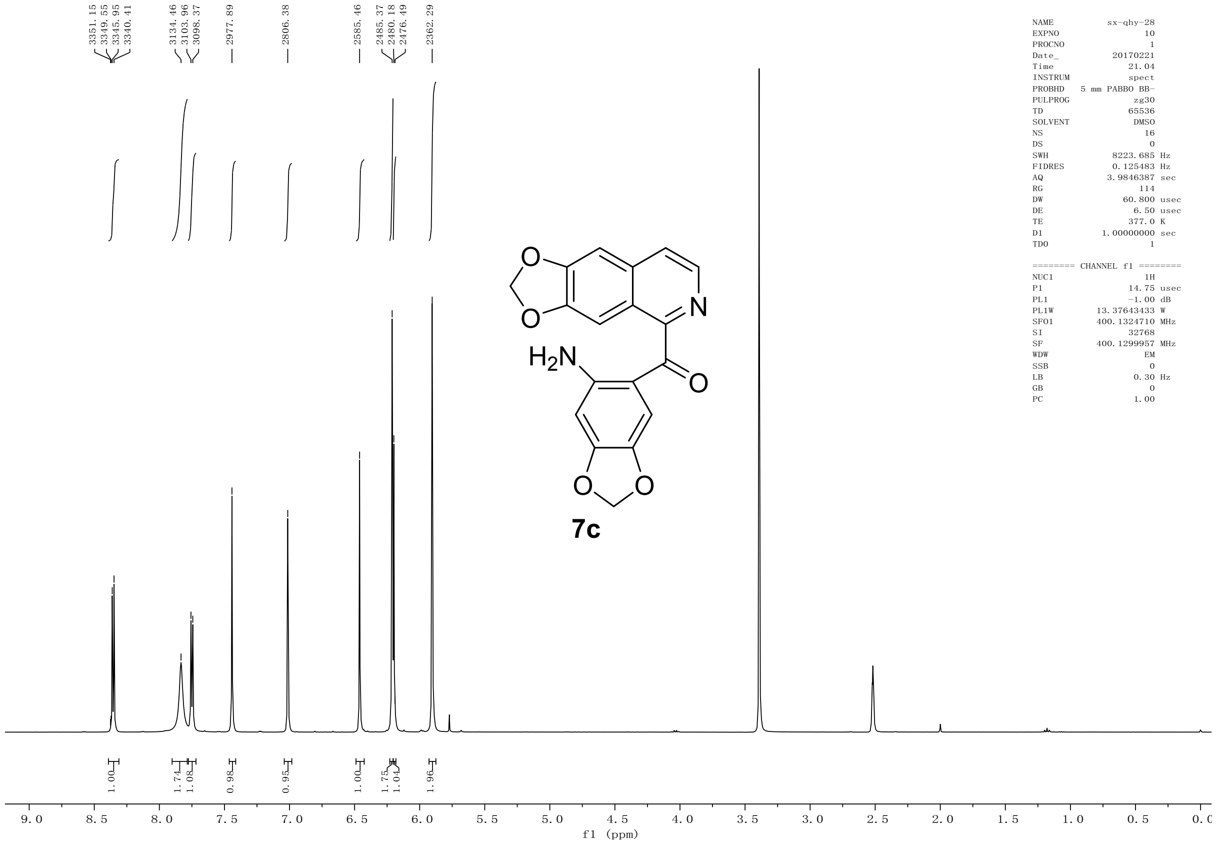

^{13}C NMR (DMSO-d6, 100 MHz) spectrum of compound **7c**:

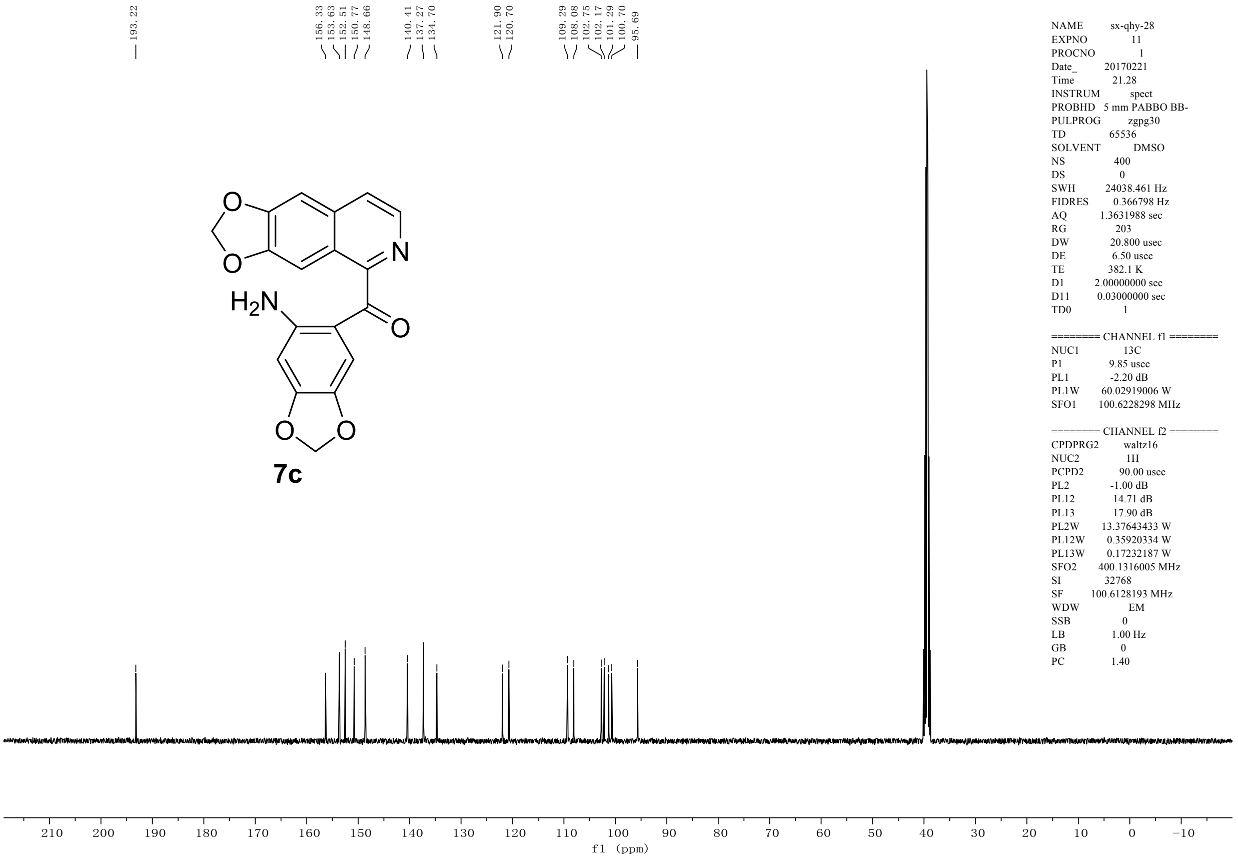

^{1}H NMR ($CDCl_3$, 400 MHz) spectrum of compound **7d**:

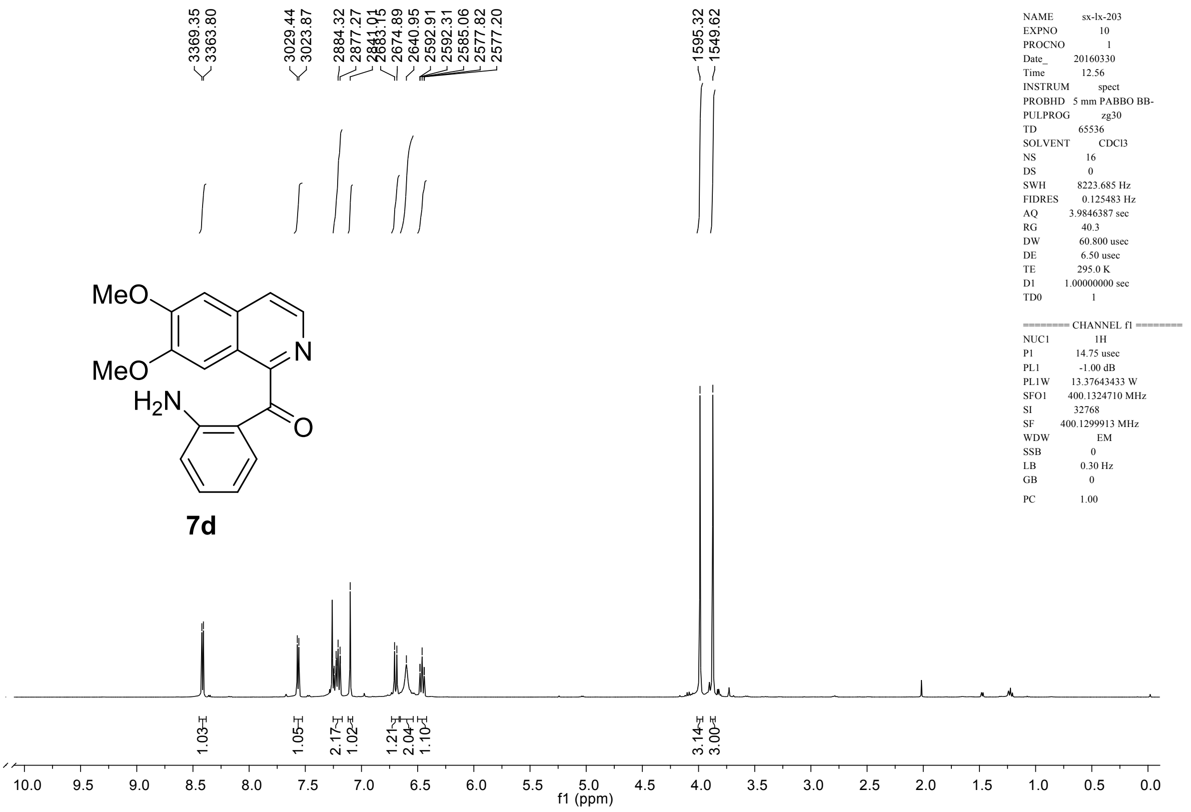

^{13}C NMR ($CDCl_3$, 100 MHz) spectrum of compound **7d**:

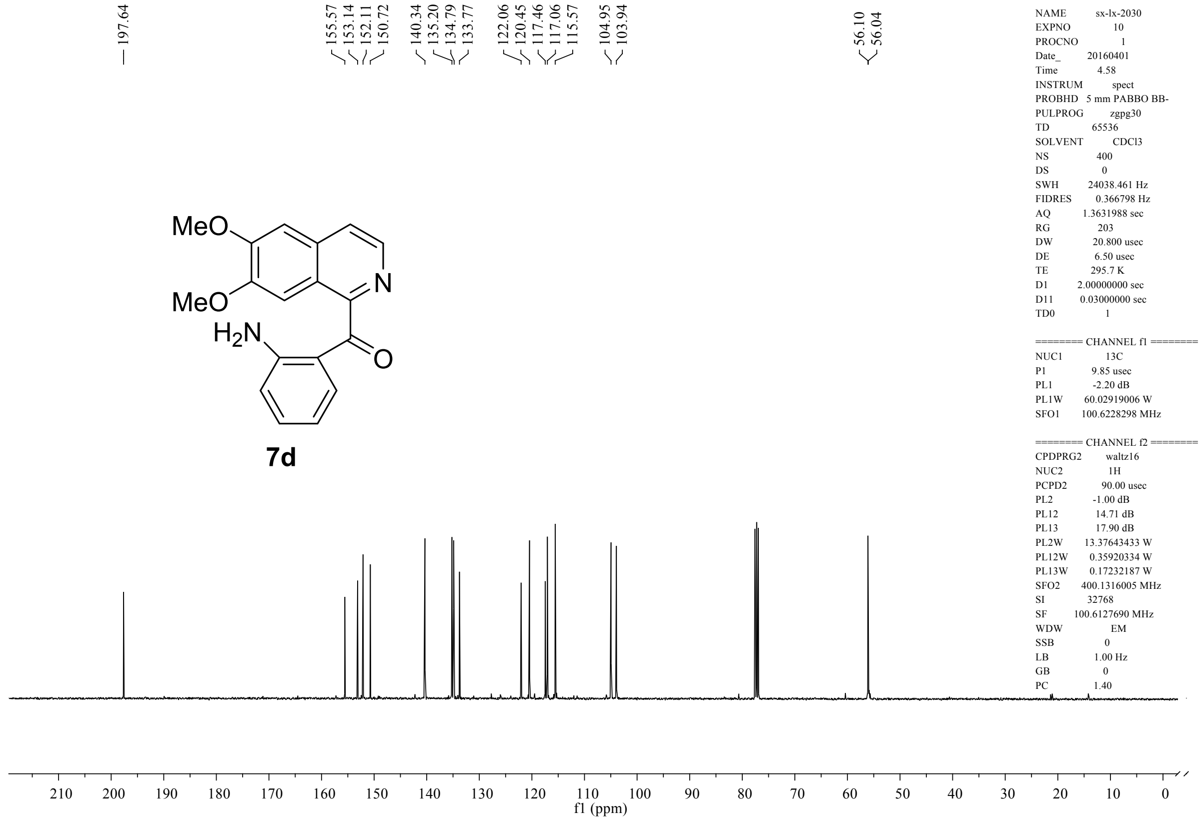

^{1}H NMR ($CDCl_3$, 400 MHz) spectrum of compound **7e**:

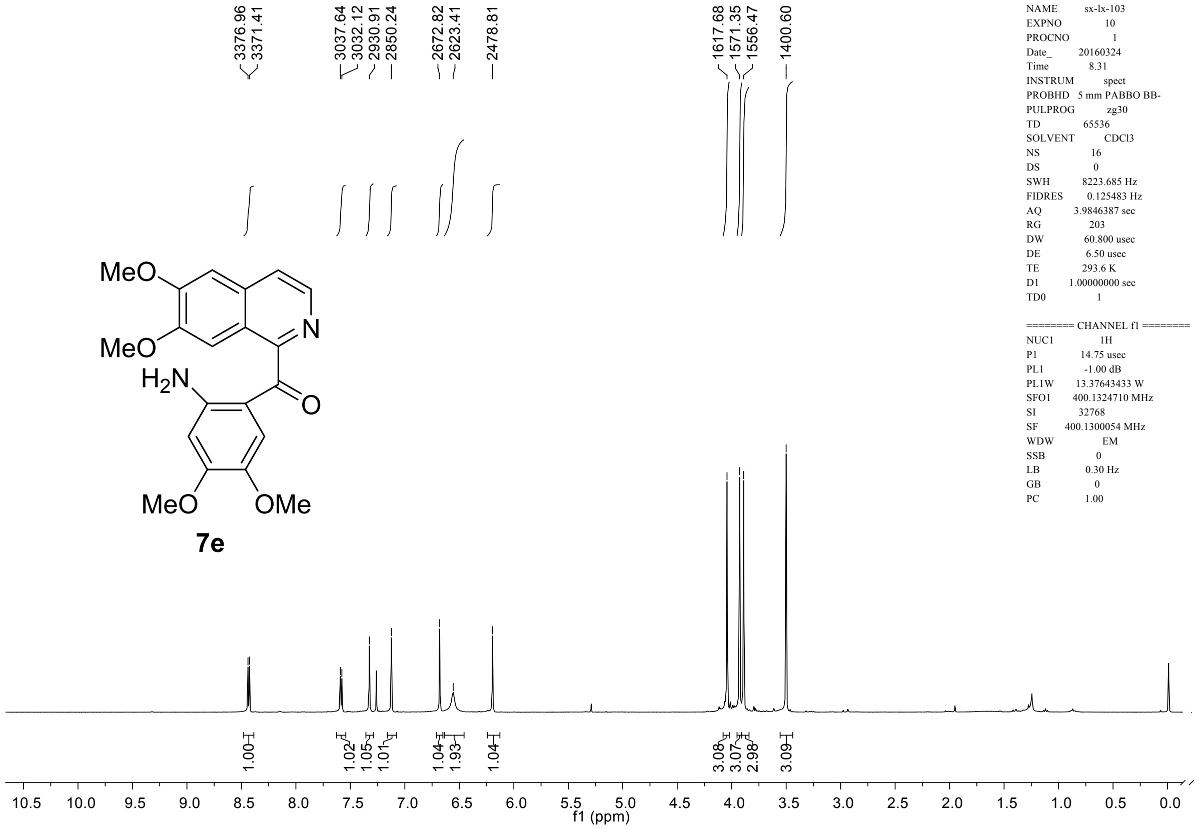

^{13}C NMR ($CDCl_3$, 100 MHz) spectrum of compound **7e**:

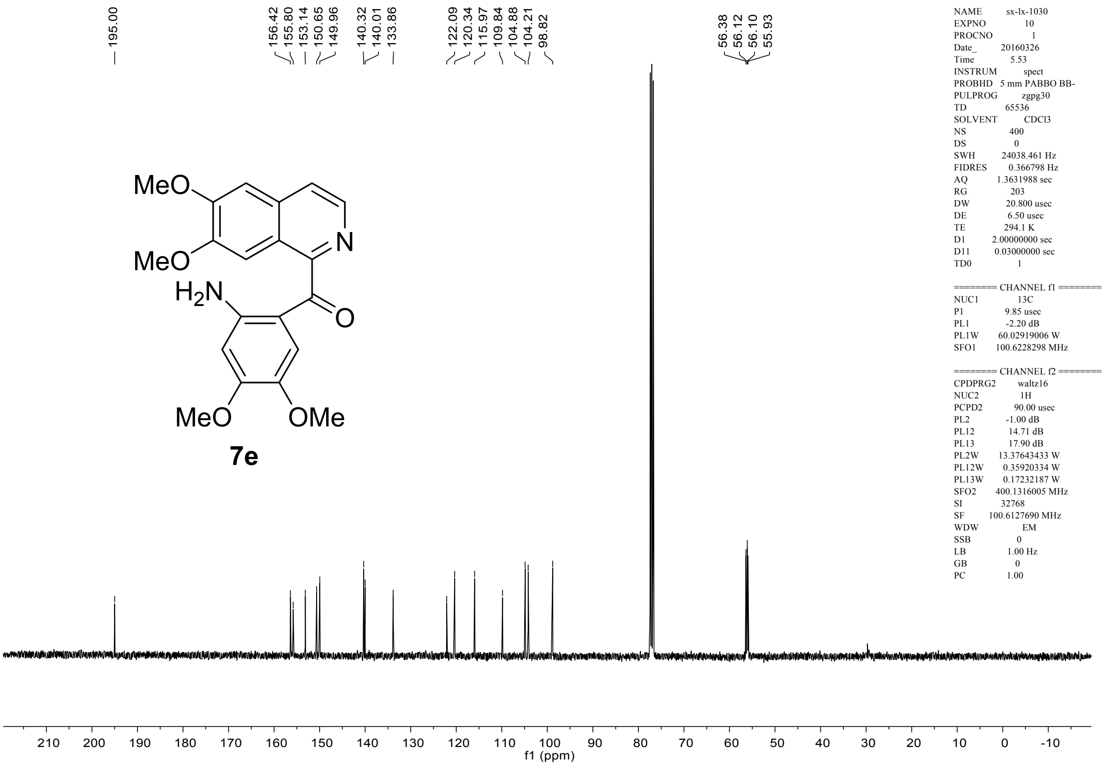

^{1}H NMR ($CDCl_3$, 400 MHz) spectrum of compound **7f**:

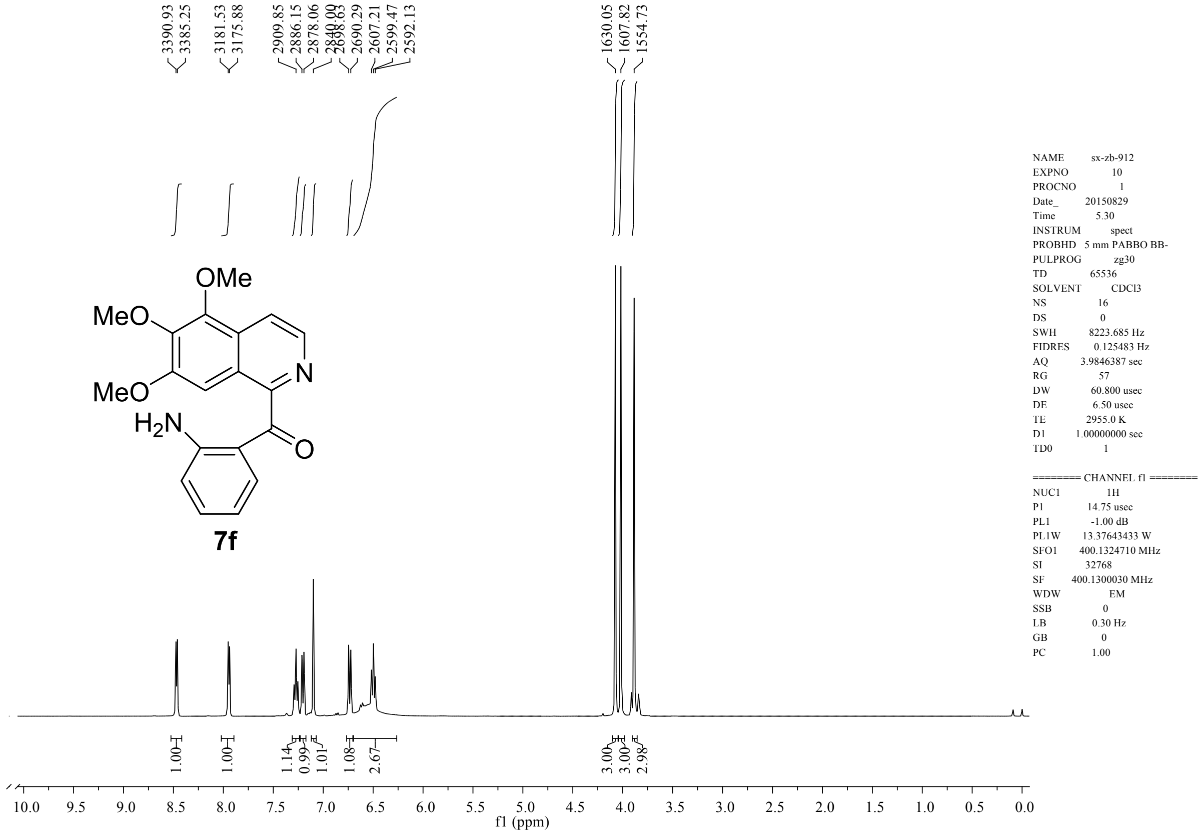

^{13}C NMR ($CDCl_3$, 100 MHz) spectrum of compound **7f**:

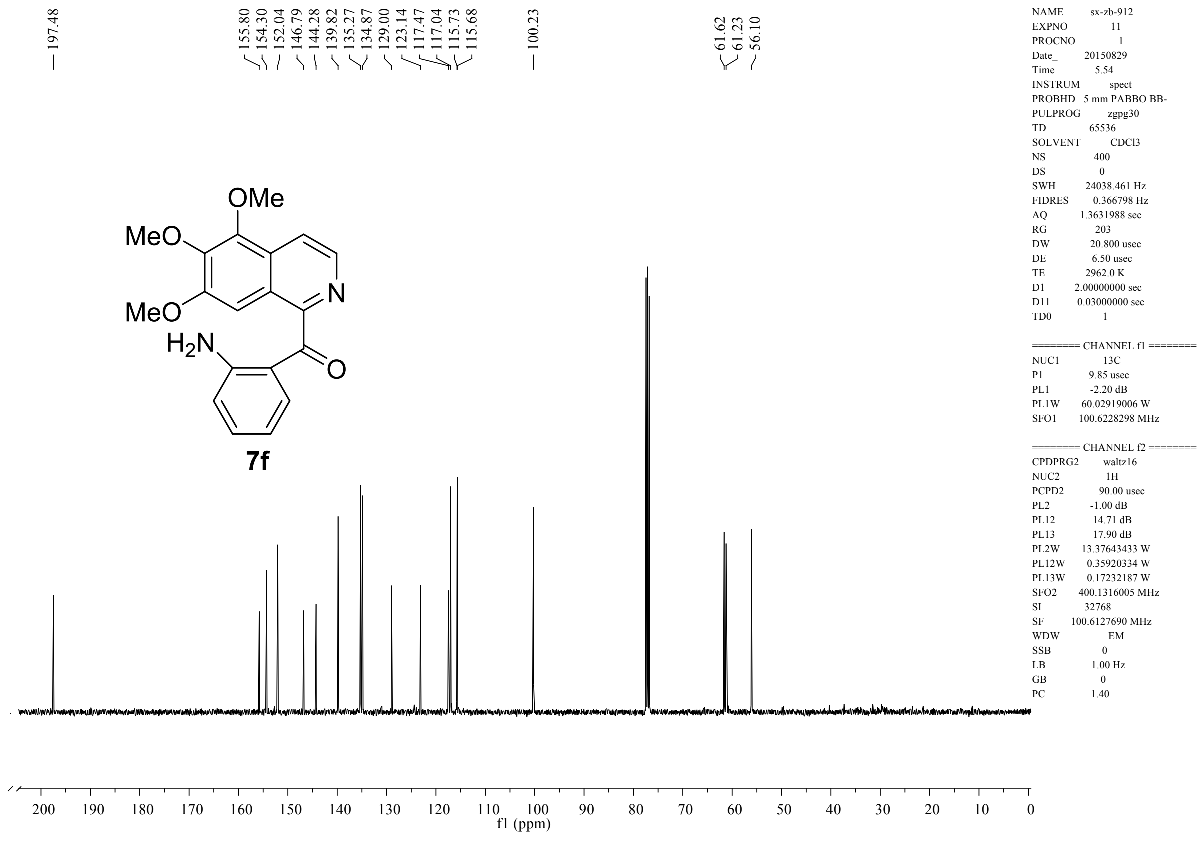

^{1}H NMR ($CDCl_3$-TFA, 400 MHz) spectrum of compound **1a**:

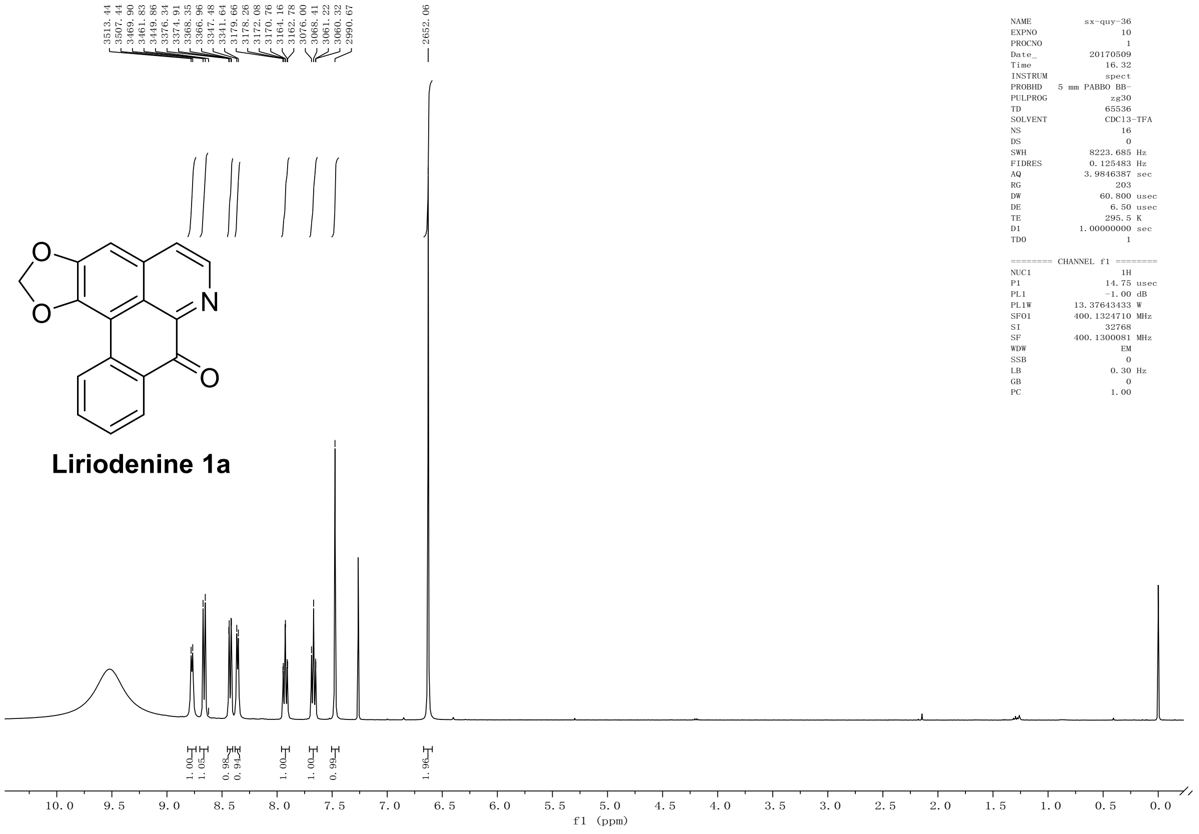

^{13}C NMR ($CDCl_3$-TFA, 100 MHz) spectrum of compound **1a**:

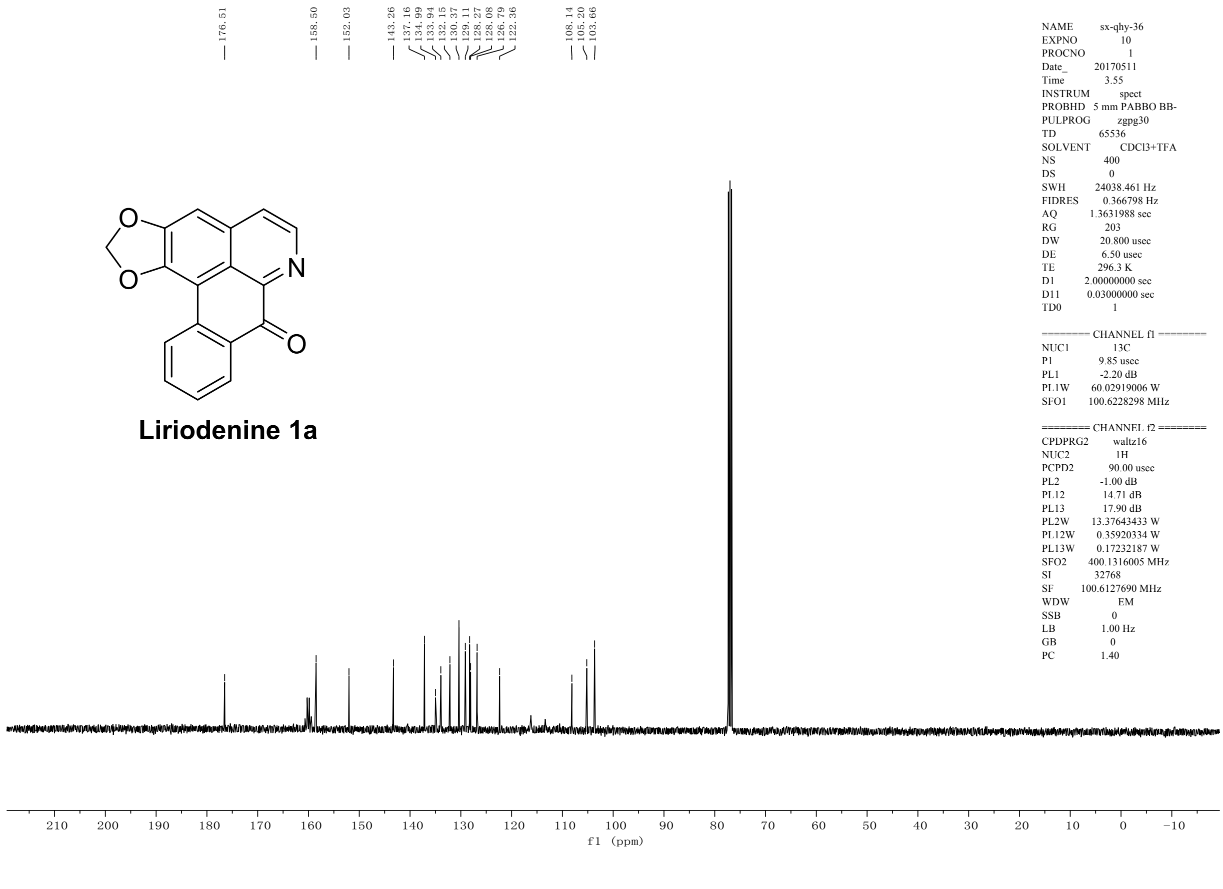

^{1}H NMR ($CDCl_3$-TFA, 400 MHz) spectrum of compound **1b**:

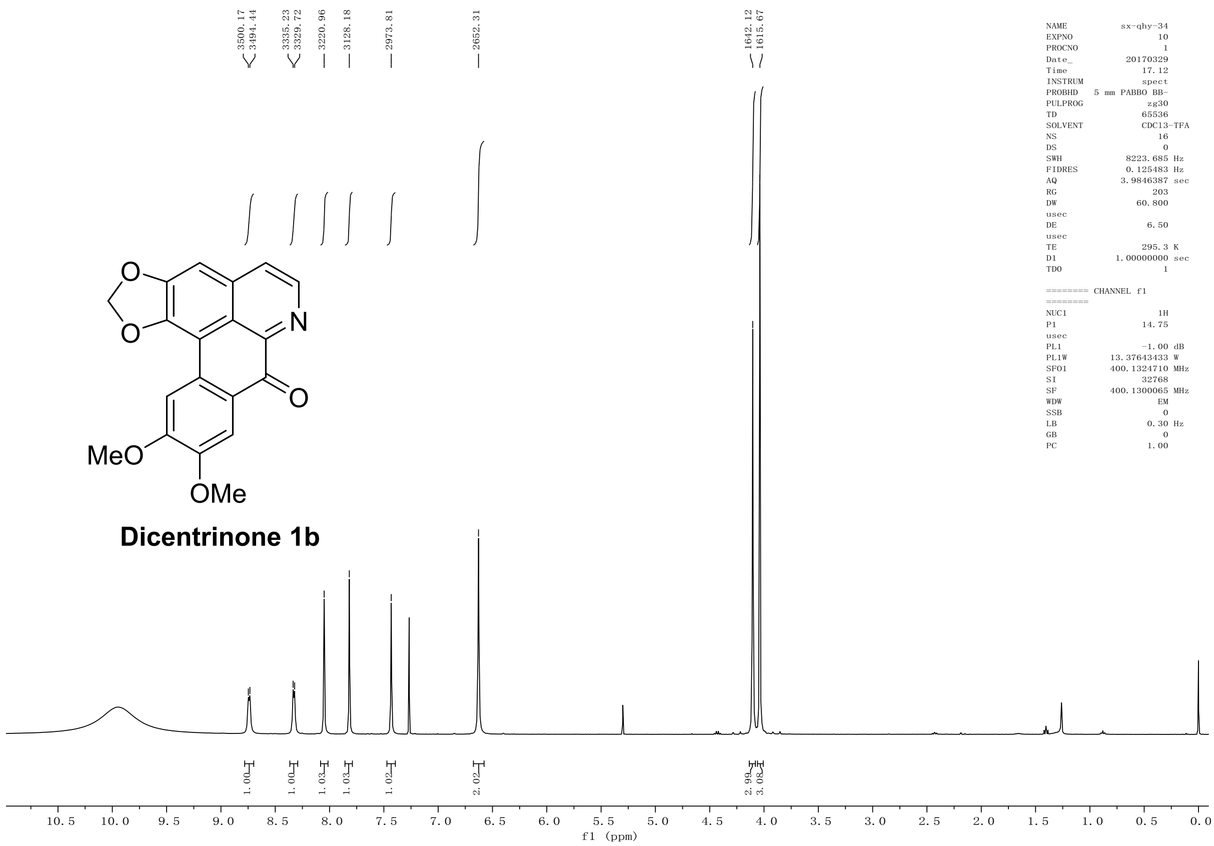

^{13}C NMR ($CDCl_3$-TFA, 100 MHz) spectrum of compound **1b**:

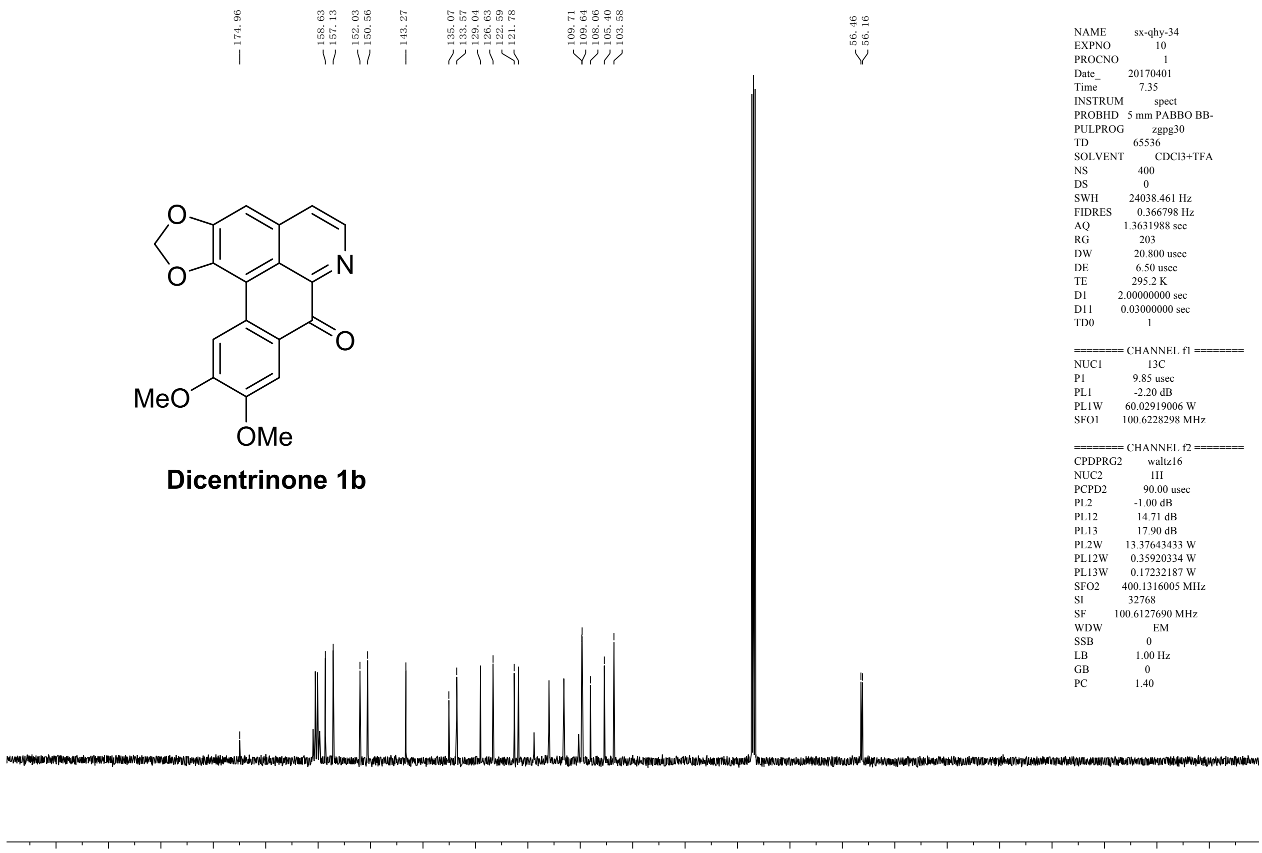

^{1}H NMR ($CDCl_3$-TFA, 400 MHz) spectrum of compound **1c**:

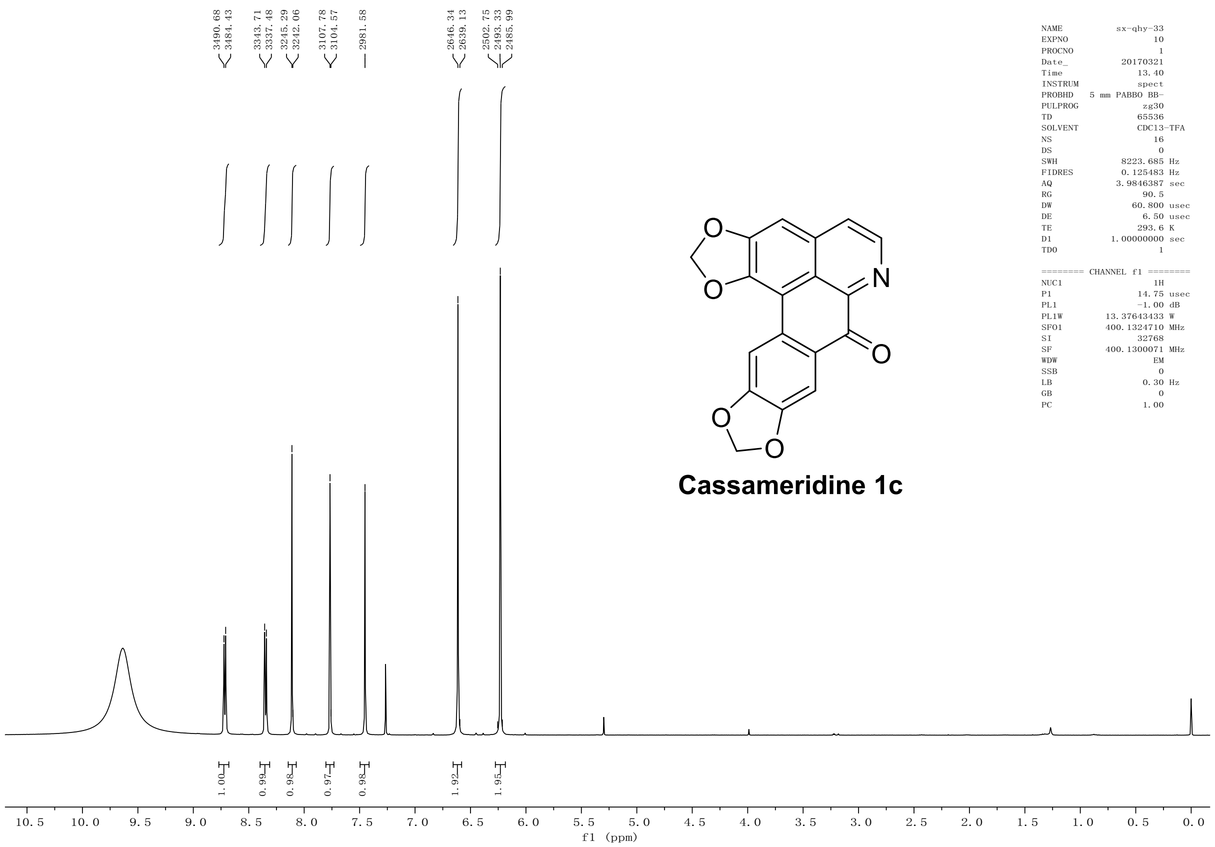

^{13}C NMR ($CDCl_3$-TFA, 100 MHz) spectrum of compound **1c**:

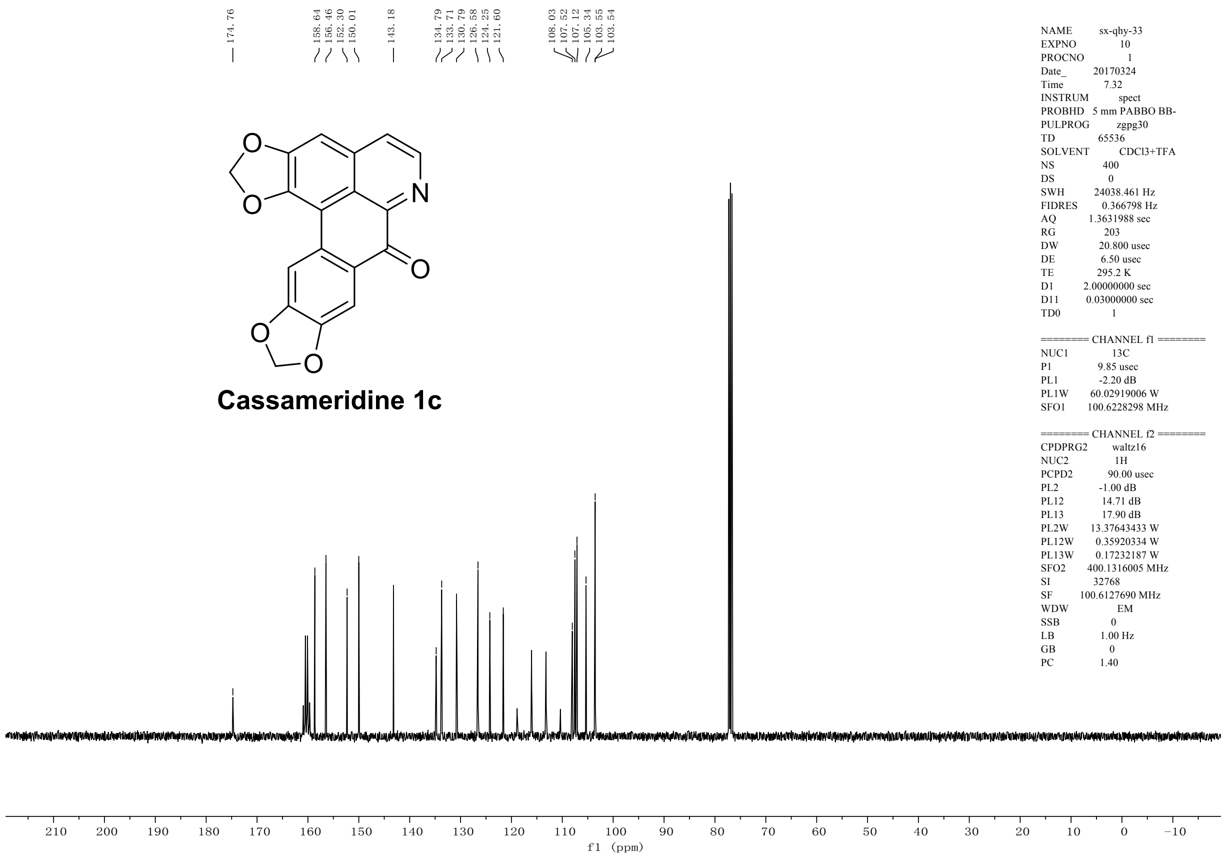

^{1}H NMR ($CDCl_3$, 400 MHz) spectrum of compound **1d**:

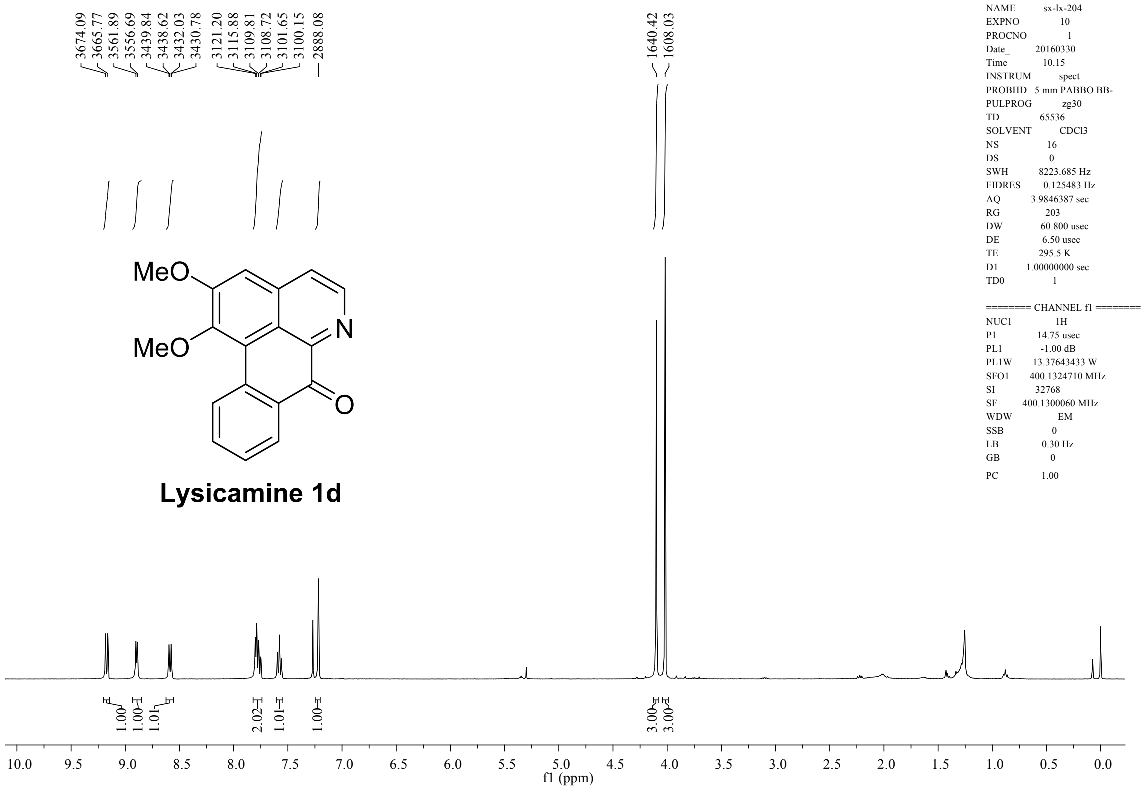

^{13}C NMR ($CDCl_3$, 100 MHz) spectrum of compound **1d**:

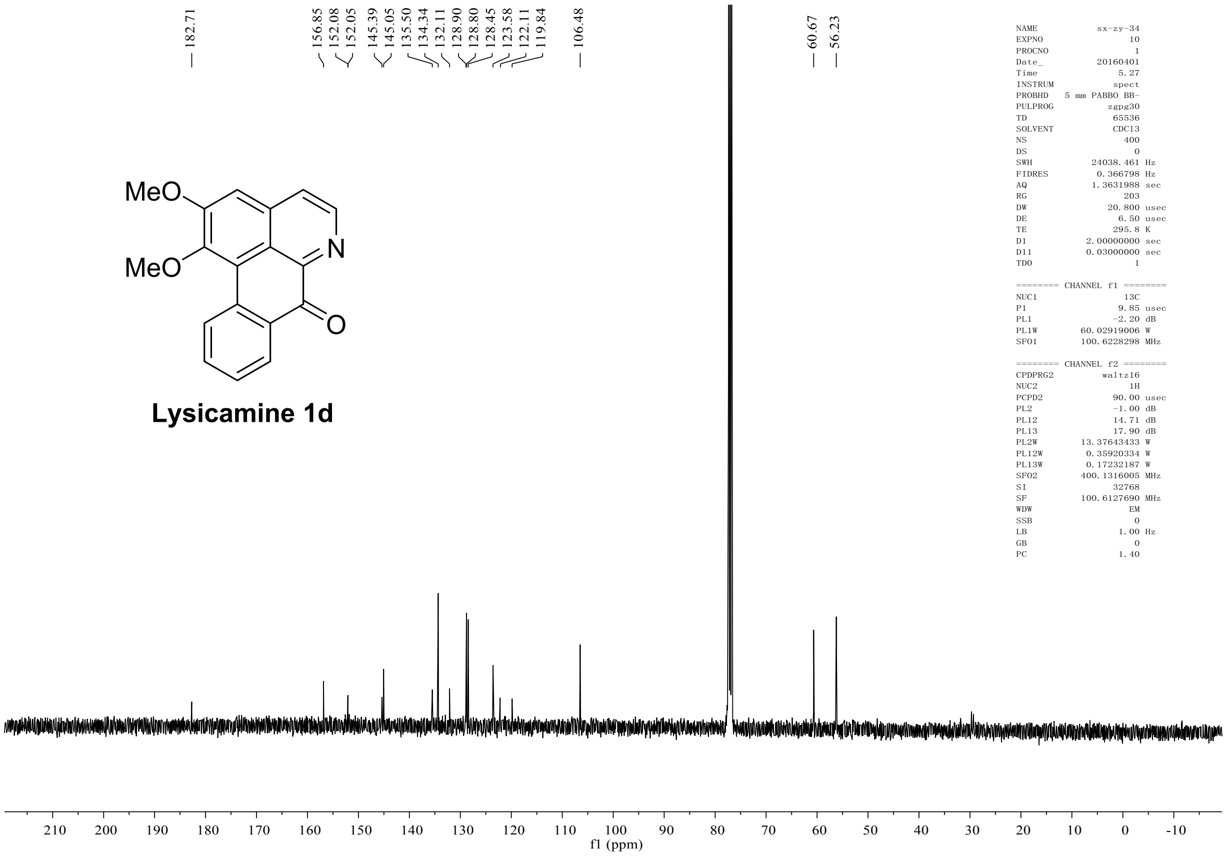

^{1}H NMR ($CDCl_3$, 400 MHz) spectrum of compound **1e**:

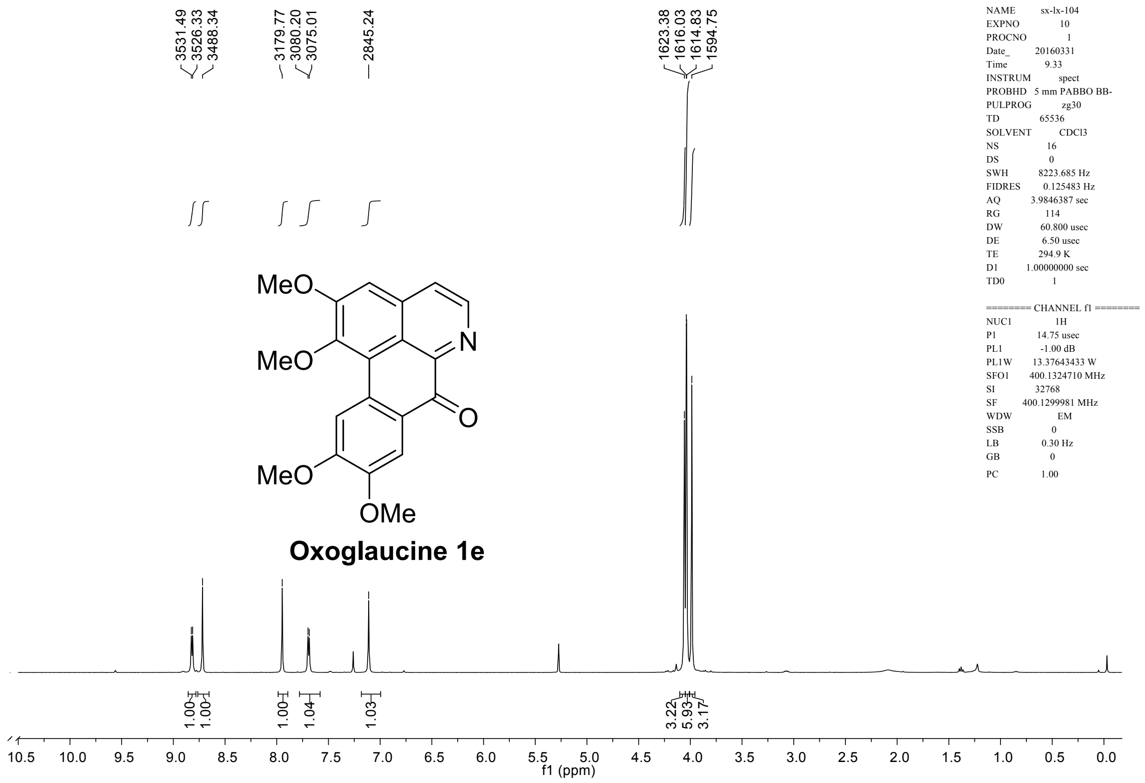

^{13}C NMR ($CDCl_3$, 100 MHz) spectrum of compound **1e**:

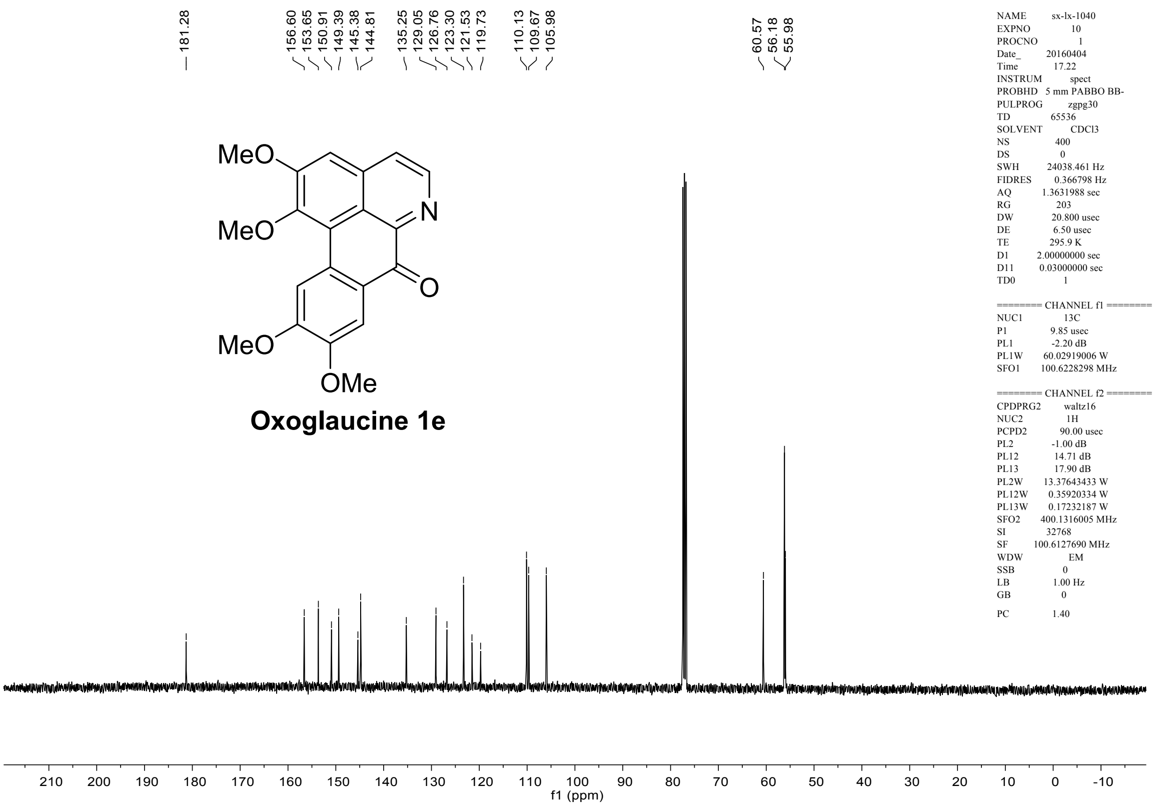

^{1}H NMR ($CDCl_3$, 400 MHz) spectrum of compound **1f**:

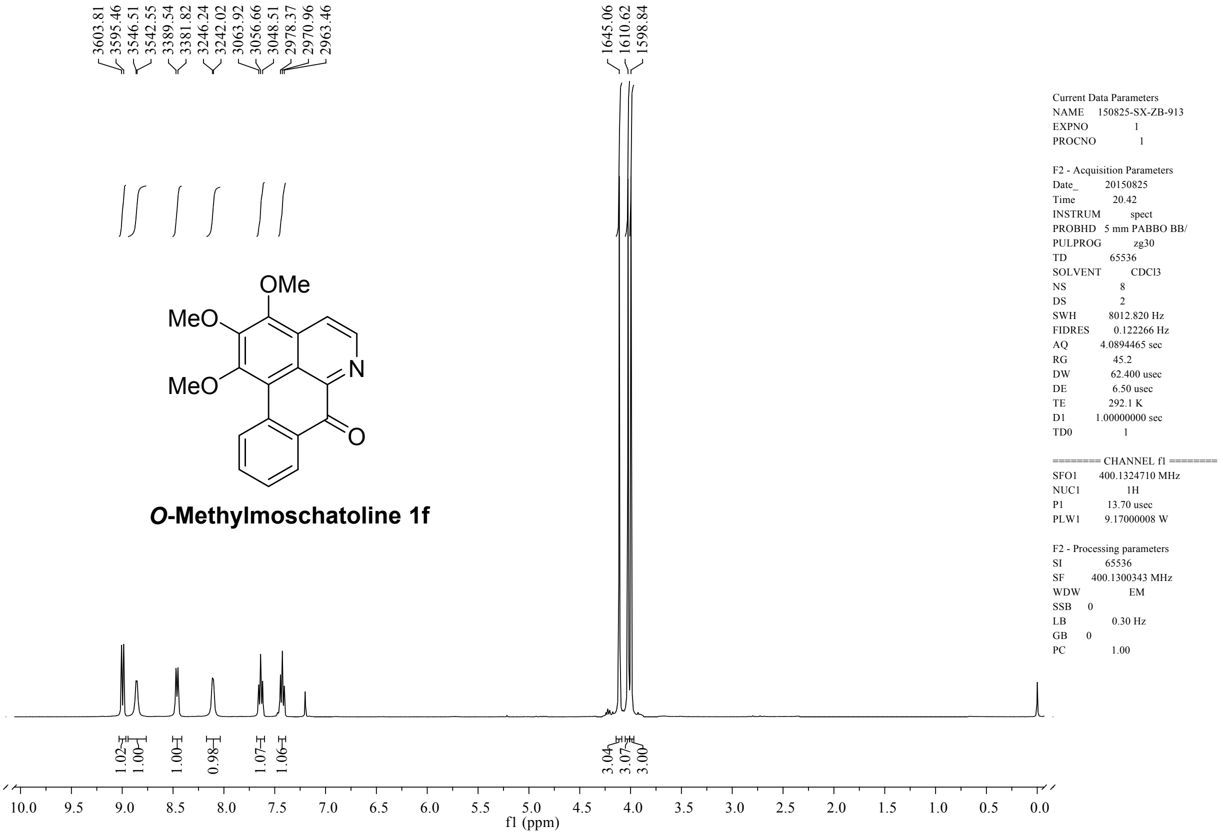

^{13}C NMR ($CDCl_3$, 100 MHz) spectrum of compound **1f**:

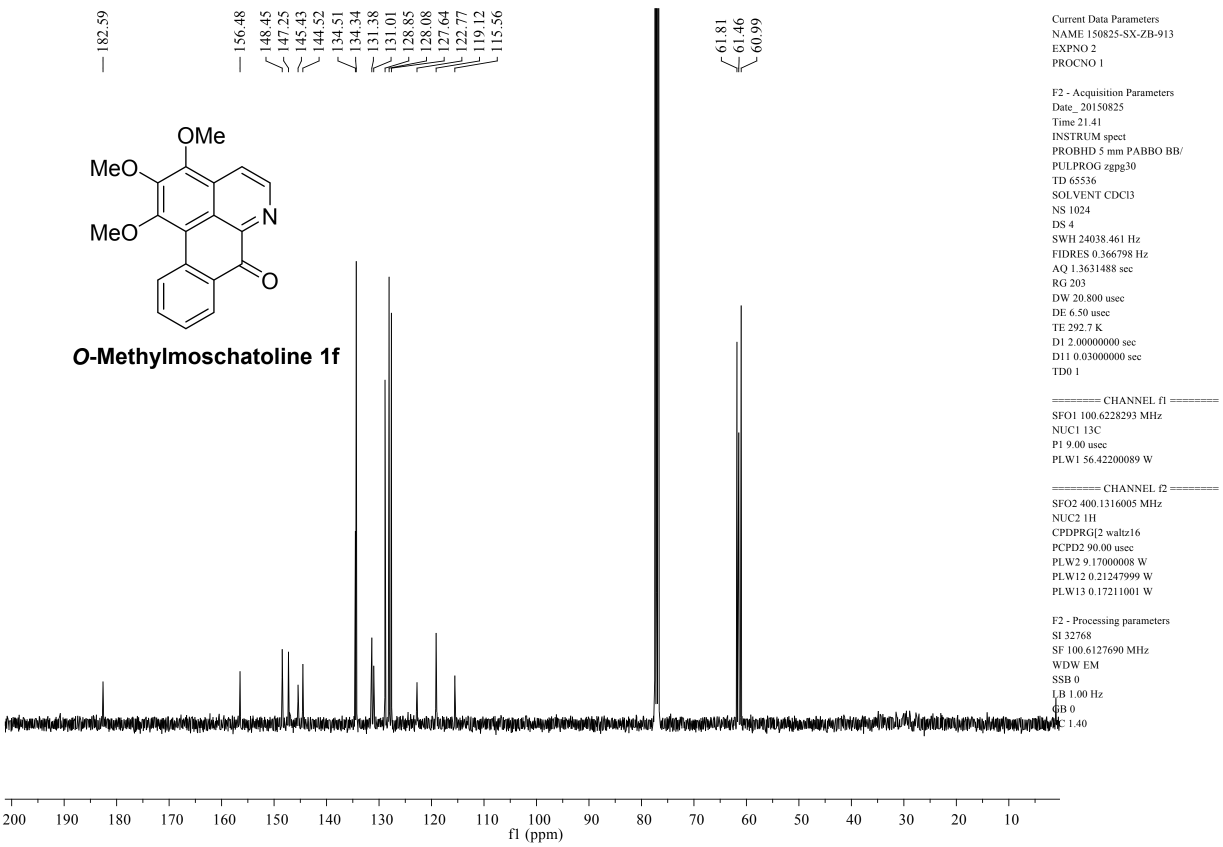

Supplement: RA-008-C8RA05338C-s001 [file RA-008-C8RA05338C-s001.pdf]
